# Molecular investigation of the radiation resistance of edible cyanobacterium *Arthrospira* sp. PCC 8005

**DOI:** 10.1002/mbo3.229

**Published:** 2015-02-12

**Authors:** Hanène Badri, Pieter Monsieurs, Ilse Coninx, Ruddy Wattiez, Natalie Leys

**Affiliations:** 1 Expert Group for Molecular and Cellular Biology, Belgian Nuclear Research Center SCK•CENMol, Belgium; 2 Proteomics and Microbiology Group, Research Institute for Biosciences, University of MonsMons, Belgium

**Keywords:** *Arthrospira*, cyanobacteria, ionizing radiation, microarray, proteomics

## Abstract

The aim of this work was to characterize in detail the response of *Arthrospira* to ionizing radiation, to better understand its radiation resistance capacity. Live cells of *Arthrospira sp*. PCC 8005 were irradiated with ^60^Co gamma rays. This study is the first, showing that *Arthrospira* is highly tolerant to gamma rays, and can survive at least 6400 Gy (dose rate of 527 Gy h^−1^), which identified *Arthrospira sp*. PCC 8005 as a radiation resistant bacterium. Biochemical, including proteomic and transcriptomic, analysis after irradiation with 3200 or 5000 Gy showed a decline in photosystem II quantum yield, reduced carbon fixation, and reduced pigment, lipid, and secondary metabolite synthesis. Transcription of photo-sensing and signaling pathways, and thiol-based antioxidant systems was induced. Transcriptomics did show significant activation of ssDNA repair systems and mobile genetic elements (MGEs) at the RNA level. Surprisingly, the cells did not induce the classical antioxidant or DNA repair systems, such superoxide dismutase (SOD) enzyme and the RecA protein. *Arthrospira* cells lack the catalase gene and the LexA repressor. Irradiated *Arthrospira* cells did induce strongly a group of conserved proteins, of which the function in radiation resistance remains to be elucidated, but which are a promising novel routes to be explored. This study revealed the radiation resistance of *Arthrospira,* and the molecular systems involved, paving the way for its further and better exploitation.

## Introduction

Why some cells are radiation sensitive and others are highly radiation resistant, is still intriguing and has been a matter of detailed investigation. The current knowledge describing the mechanisms or conditions that likely contribute to radiation resistance indicates that radiation resistance correlates not exclusively with the induced radiation damage to DNA, but rather with the susceptibility of the cellular proteins to radiation-induced oxidation (Krisko and Radman 2010). The ability of cells to protect their proteins from oxidation by scavenging the harmful reactive oxygen species (ROS) generated by ionizing radiation (IR) has been proposed as the key mechanism for survival of IR-resistant microorganisms. Due to the accumulation of small antioxidants molecules in *Deinococcus radiodurans* (Daly et al. 2010), *Halobacterium salinarum* (Robinson et al. 2011), and the bdelloid invertebrate Adineta Vaga (Gladyshev and Meselson 2008), they can protect their protein from oxidation and thereby preserve the function of enzymes needed to repair DNA and survive.

Most studies, however, have used non-photosynthetic test organism. Nevertheless, also some cyanobacteria were reported to be UV (Vass et al. 2013) and even X-ray (Billi et al. 2000) and gamma ray radiation resistant (Singh et al. 2010a, 2013), which makes them interesting study objects to further unravel the molecular principles of cellular radiation resistance of photosynthetic organisms. *Arthrospir*a is a free-floating filamentous cyanobacterium that tends to aggregate and grows vigorously in water of alkaline lakes (Vonshak et al. 1988), and in regions with strong sunshine and high temperature (Hongsthong et al. 2009). *Arthrospira* is not pathogenic in nature and has been used for human consumption since 16th century, due to its high-protein content and easy digestible property (Dillon et al. 1995). Its valuable nutritious components include essential fatty acids such as omega-3, and pigments, such as carotenes and phycocyanin (Vonshak 1990; Ramadan et al. 2008). The last decades, *Arthrospira* has gained increasing interest as health promoting food supplement, on earth and for human space flight (Hendrickx et al. 2006). In specific, its strong anti-oxidant and anti-inflammatory properties are subject of investigation and seem promising for potential application in human radiation protection (Bhat and Madyastha 2001). In fact, *Arthrospira* has been used in the nutraceutical “Spirulina” to treat radiation sickness (Belay 2002).

The aim of this work was to characterize in detail the response of *Arthrospira* to IR, to better understand its peculiar cellular protection against radiation. Therefore, the cellular and molecular response of *Arthrospira* strain PCC 8005 to high acute doses of gamma rays was investigated using transcriptomics and proteomics. Previously, the resistance and response of algea or cyanobacteria to radiation has been mainly investigated by morphological and physiological analysis (Kraus 1969; Agarwal et al. 2008; Singh et al. 2010a, 2013), but the molecular mechanisms remain to be elucidated. To our knowledge, this is the first study that investigates the tolerance of the edible cyanobacterium *Arthrospira* to IR, at molecular level. The complete genome sequencing of *Arthrospira* sp. PCC 8005 was recently defined (Janssen et al. 2010). Based on this genome sequence, a novel full-genome covering DNA-microarray, specific to *Arthrospira* sp. PCC 8005*,* was designed, and used for the first time in this study to monitoring expression genes in response to radiation. In addition, transcriptomic analyses were combined with proteomic and phenotypic analysis.

## Materials and Methods

### Strain and culture conditions

The strain *Arthrospira* sp. PCC 8005 was obtained from The Pasteur Culture Collection. Three independent cultures (*n* = 3) were used for irradiation. The three replicates were grown separately on a rotatory shaker in an incubator at 30°C (Binder KBW 400), in 600 mL Zarrouk medium (Cogne et al. 2003) to mid-exponential phase corresponding to an OD_750_ ∽1. Cultures were illuminated with a photon flux density of ∽42 *μ*E m^−2^ sec^−1^ provided by three Osram daylight tubes. Each 600 ml culture was then divided in six aliquots into different flasks, which were further divided into nonirradiated controls and samples for irradiation (CELLSTAR® Filter cap cell, Greiner Bio-One, vilvoorde Belgium, 250 ml cell culture flasks). Irradiation was carried out on active planktonic filaments suspended in 40-mL aerobic liquid Zarrouk culture medium pH 9.5, at cell concentrations of ca. OD_750_ ∽1.

### Irradiation conditions

The irradiation was performed using RITA facility at the Belgian Reactor No. 2 (BR2) (Fig. S1). The irradiation was carried out in dark, inside a closed canister, surrounded by four sources of ^60^Co gamma rays (energy of 1.33 and 1.17 Mev). Different doses of gamma rays were given at a constant dose rate of 527 Gyh^−1^. Figure S1 illustrate the 6 different tested doses and the occurred irradiation time. The cultures were in the dark, and the temperature inside the irradiation canister was automatically monitored and ranged between 26°C and 27°C. The time required for irradiation was dose-dependent and so respective controls were kept for the same time in the dark as irradiated samples. Samples were immediately put on ice after irradiation, at the irradiation facility, and before transport to the laboratory for further processing. Some aliquots were used immediately after irradiation for regrowth and measurement of chlorophyll fluorescence. While the main part of the samples was centrifuged at 4°C, and the obtained cell pellet was flash frozen in liquid nitrogen, and further conserved at −80°C, for molecular and biochemical analysis, including mRNA, protein, and pigment content.

### Postirradiation recovery and proliferation

In order to investigate the ability of *Arthrospira* filaments to recover after irradiation, inoculation of 1% (v/v) from irradiated and nonirradiated samples was carried out in fresh medium, and incubated for growth at the same conditions as cited above. The growth was followed by absorbance measurement at OD 750nm (optical density) every 5 days using the spectrophotometer AquaMate, Unicam, Cambridge, UK. The proliferation curves were made based on OD_750nm_ versus time.

### Photosynthetic potential measurement

Chlorophyll A and phycobilisome fluorescence of PSII was determined using the DUAL PAM 100 (Waltz-GmbH, Effeltrich, Germany). From three independent cultures for each test condition (*n* = 3), aliquots of two ml of control cultures and irradiated samples were tested immediately after exposure. All samples were dark adapted for 15 min. Then, the cells were exposed to a weak modulated red light (ML) (635 nm, 3 *μ*E m^−2^ sec^−1^) (which is too low to excite and induce any photosynthetic activity or fluorescence), and minimum fluorescence was determined (*F*_0_). Next, the cells were exposed to a high-red light excitation called saturating pulse (635 nm, 8000 *μ*E m^−2^ sec^−1^) with short duration (0.8 sec) and maximum fluorescence in dark adapted state (*F*_m_) was determined. From those measurements, the ratio *F*_v_/*F*_m_ was then calculated, where the variable fluorescence *F*_v_ = *F*_m_ − *F*_0_, and present the difference between maximum fluorescence from fully reduced PSII reaction center (Fm) and the intrinsic fluorescence (*F*_0_) from the fully oxidized PSII. Healthy *Arthrospira* cells normally have a yield *F*_V_/*F*_M_ of ca. 0.6 (Masojídek et al. 2010), while photosynthetically non-functional (dead) cells have *F*_v_/*F*_m_ of 0.

### Pigments analysis

From three independent cultures for each test condition (*n* = 3), aliquots of one ml of irradiated and control cultures were collected immediately after exposure to gamma rays by centrifugation (5418R; Eppendorf Robelaar, Belgium) (10,000*g*, 15 min), and cell pellets were stored at −80°C until analysis (ca. 2 days). Later, frozen cell pellets were freeze-dried overnight using a freeze-dryer (Lyovac GT 2, Sweden), and the absolute dry weight was determined. Next, the pellet was resuspended in 1 mL of 0.05 molL^−1^ Na_2_HPO_4_ at pH = 7, in order to extract the hydrosoluble fraction of phycobiliproteins containing phycocyanin and allophycocyanin pigments. To break the cells, five cycles of freezing in liquid nitrogen and thawing at 37°C were performed. And, in order to achieve total extraction, additional treatment with lysozyme at a final concentration of 100 mg mL^−1^ was carried out. Next, the lysed fraction was centrifuged (10,000*g*, 10 min), and the supernatant, was measured at wavelengths 615 and 652 nm. The concentration of phycocyanin and allophycocyanin were calculated according to (Bennett and Bogorad 1973). Then, the pellet remaining after extraction of the hydrosoluble fraction, was washed three times using 1 mL of 0.05 molL^−1^ Na_2_HPO_4_ at pH = 7 and then used for a Chlorophyll extraction with 100% methanol as organic solvent. Additional mechanic treatment by sonication (three cycles of 10 sec, amplitude 30%, 1 pulse rate [Sonics Vibra cells], Newtown, USA) was performed to allow total chlorophyll extraction. The lysed fraction was centrifuged (13,000*g*, 10 min) and the supernatant was measured via spectrophotometry at a wavelength 665 nm. Chlorophyll concentration was calculated then according to Bennett and Bogorad (1973).

### RNA extraction

RNA extractions were performed on three independent cultures for each test condition (*n* = 3). The RNA extraction procedure had to be optimized. In total, 30 mL of irradiated and control *Arthrospira* cultures were put on ice immediately after irradiation, and were centrifuged (Avanti J- 26XP; Beckman Coulter, Suarlée, Belgium) for 20 min at 10,000*g* and 4°C, to collect the cell pellets (in falcon tubes of 15 mL). Cell pellets were then flash frozen in liquid nitrogen and stored immediately at −80°C, until analysis (ca. 5 days). Before extraction, the frozen cells were mixed with 1 mL Trizol (Invitrogen, Life Technologies Europe B.V, Ghent, Belgium) and, transferred into 2 mL Eppendorf tubes, so that the cells were already in the lysis solution (also preventing enzymatic activity for RNA degradation) during defrosting. The breakage of the cells was carried out by applying a heat shock procedure, i.e., cells suspended in Trizol were incubated at 95°C for 5 min and then submerged immediately on ice for additional 5 min. Next, the released RNA was separated from the cell debris by centrifugation (5418R; Eppendorf) at 10000 g, for 10 min at 4°C. RNA purification was performed at 4 °C using the Direct-zol RNA miniprep 2050 (Zymosearch) following the manufacturer's instructions. The volume ratio of the aqueous and organic phases was 1:1. RNA samples (150 *μ*g) were then treated 30 min at 37°C using DNAse (Ambion TURBO DNA-free™, Life Technologies Europe B.V, Ghent, Belgium) following the manufacturer's instructions. The RNA was concentrated at 4°C using RNA Clean & Concentrator™-25 (Zymo Research, by S.A Laborimpex NV, Brussels, Belgium). RNA quantity and purity was assessed by spectrophotometric analysis using NanoDrop ND-1000 Spectrophotometer (Thermo Scientific, Isogent Life science, Temse Belgium). The quality and integrity of RNA was assessed using the Bioanalyzer 2100 (Agilent Technologies, Diegem, Belgium) according to manufacturer's instructions. It is worthwhile to mention, that the Bioanalyser RNA profiles for *Arthrospira* do not allow to determine “RNA integrity number” (RIN) values as the profiles are different from standard profiles obtained for most other bacteria. The rRNA profile for *Arthrospira* contains three fragments (three peaks) instead of two, representing 16S and 23S rRNA, as has also been reported for *Nostoc punctiforme* (Pinto et al. 2009).

### Microarray design

The full genome of *Arthrospira* sp. PCC8005 was sequenced by Genoscope (Team of Dr. Valerie Barbe) and Version 3 (692 contigs, ∽6.8 Mbp) of this genome (Janssen et al. 2010) was used as input for the Nimblegen, WI, USA microarray design. A tiling array “*Arthrospira* HX12” was designed, with probes ranging from 50 up to 72 nucleotides and an average length of 53 nucleotides, and an average spacing of 34 nucleotides between two different probes. The 135 367 probes – excluding random and control probes – were mapped back to the improved version 5 of the genome, currently publically available at EMBL database (accession number: CAFN01000000) and imported in the Microbial Genome Annotation & Analysis Platform (MaGe) allowing private expert annotation of genes, which could be grouped to 5854 CDS and 3141 intergenic regions. For the production of *Arthrospira* HX12 microarray chips, the 12 × 135k array format of Roche NimbleGen (Madison, wI, USA) was used.

### RNA analysis via microarrays

For two radiation doses tested (i.e., 3200 and 5000 Gy), the total RNA extracts of three irradiated cultures and their equivalent three nonirradiated cultures (*n* = 3) were collected. At Institute for Research of Biomedicines in Barcelona (IRBB) in Barcelona, Spain cDNA library preparation and amplification were performed on 25 ng of this total RNA, using the Complete Whole Transcriptome Amplification WTA2 kit (Sigma-Aldrich, Diegem, Belgium) and according to the instructions of the manufacturer with 17 cycles of amplification, resulting in microgram quantities of cDNA. Labeling and hybridization of the cDNA onto the new designed *Arthrospira* HX12 arrays (Nimblegen, Madison, wI, USA), were performed according to Roche-Nimblegen expression guide v5p1. For each sample, 1 *μ*g cDNA was labeled by Cy3 nonamers primers and Klenow polymerization. Hybridization mixture with 2 *μ*g Cy3-labeled cDNA was subsequently prepared. Samples were hybridized to *Arthrospira*_ HX12 array Roche-NimbleGen for 18 h at 42°C. The arrays were washed and scanned in a Roche-Nimblegen MS 200 scanner. Raw data files (Pair and XYS files) were obtained from images using DEVA software (Roche-Nimblegen) and provided by IRBB to SCK•CEN for data analysis.

### Microarray data analysis

Both for 3200 Gy and for 5000 Gy, three microarrays of irradiated cultures and three microarrays of their equivalent nonirradiated cultures (*n* = 3) were analyzed. Raw data were preprocessed using the “Oligo” package (version 1.24) in BioConductor (version 2.12/R version 3.0.1) as follows: (1) background correction based on the Robust Multichip Average (RMA) convolution model (Irizarry et al. 2003), (2) quantile normalization to make expression values from different arrays more comparable (Bolstad et al. 2003), and (3) summarization of multiple probe intensities for each probe set to one expression value per gene using the median polish approach (Irizarry et al. 2003). To test for differential expression between the different irradiated conditions and the reference conditions (no irradiation) the Bayesian adjusted t-statistics was used as implemented in the “LIMMA” package (version 2.18.0) (Smyth 2004). *P*-values were corrected for multiple testing using the Benjamini and Hochberg's method to control the false discovery rate (Benjamini and Hochberg 1995). Transcripts were considered significantly differentially expressed when the corresponding adjusted *P* < 0.05 and their absolute fold change (FC) was equal or larger than 2 for upregulated genes, and equal or miner than 0.5 for the downregulated ones. The FC is the parameter measuring the change in the expression level of a gene between two conditions, for example, irradiated versus non-irradiated. Gene annotation was curated manually based on the manual expert annotation available in MaGe (ARTAN consortium) and further curated manually during this work.

### Protein extraction and analysis

Protein extractions were carried out on three independent cultures for each test condition (*n* = 3). Aliquots of two ml samples were centrifuged (5418R; Eppendorf) at (10,000*g*, 10 min) and cell pellets were stored at −80°C immediately after irradiation. For protein extraction, pellets were resuspended in ∽100 *μ*L of 6 molL^−1^ guanidine chloride solution pH 8.5 (Lysis buffer of ICPL Kit [Serva, Heidelberg Germany]), and cells were lysed by sonication (U50 IKAtechnik, Boutersem, Belgium) (three cycles of 10 sec, amplitude 30%, 1 pulse rate) on ice. The samples were subsequently centrifuged (5418R; Eppendorf) at 16,000*g* at 4°C for 15 min, to separate the soluble proteins from the insoluble cell debris. The total protein concentration was determined using the Bradford method with the Bio-Rad Protein Assay kit (Bio-Rad, Hertfordshire, UK) according to the manufacturer's instructions, using bovine gamma globulin as a protein standard. Exactly 100 *μ*g of proteins were treated to reduce their disulphide bounds using 0.5 *μ*L of 5 mmolL^−1^ Tris (2-carboxyethyl) phosphine (ICPL-SERVA Kit) at 60°C for 20 min and then alkylated using 0.5 *μ*L of 0.4 mmolL^−1^ iodoacetamide (ICPL-SERVA Kit) at 25°C for 20 min. The reaction was stopped by adding 0.5 *μ*L of stop solution (ICPL-SERVA Kit). Proteins were recovered by acetone precipitation during at least 2 h, using an acetone/protein ratio of 4:1 (V/V). Next, after a 15-min centrifugation at 16,000*g* and an acetone evaporation, the resulting pellet was dissolved 800 *μ*L of 50 mmolL^−1^ ammonium bicarbonate containing 20 *μ*g of trypsinePromega v5111 (Promega Benelux, Leiden, The Netherlands). The enzymatic digestion of the proteins to peptides was performed overnight at 37°C. The digestion was stopped by adding formic acid (0.1% final v/v). Peptides were separated via reversed phase liquid chromatography and eluted with a gradient of acetonitrile from 10% to 35% during 120 min. Peptides were then ionized via Electron spray ionization source (ESI) at 150°C and then analyzed via Triple TOF 5600 (AB) SCIEX, Niewerker aan den ijssel, The Netherlands).

Protein identification was performed against a local copy of the *Arthrospira* sp. PCCC 8005 genome version V5 using ProteinPilot Software v4.1 and the Paragon algorithm (4.0.0.0, 459) AB Sciex. Search parameters included trypsin digestion and cysteine alkylation set to iodoacetamide. Processing parameters were set to “Biological modification” and a thorough ID search effort was used. Mass tolerance was set to 10 ppm in MS and 0.05 Da in MS/MS. Peptide FDR rate was set to 5% or less (*P* < 0.05) based on decoy database searching and all peptides included for analysis had a score representing ≤1% FDR (*P* ≤ 0.01) in at least one of the search engine results. In addition, all peptides were manually inspected.

For protein quantification, the protein needed to be represented by at least one unique peptide with 95% confidence (*P* < 0.05). MS1 chromatogram-based quantitation was performed in Skyline (MacLean et al. 2010) (http://proteome.gs.washington.edu/software/skyline/). Details for MS1 Filtering and MS1 ion intensity chromatogram processing in Skyline were described recently in detail by Schilling et al. (2012). Briefly, comprehensive spectral libraries were generated in Skyline using the BiblioSpec algorithm (Frewen and MacCoss 2002) from database searches of the raw data files prior to MS1 Filtering. Subsequently, raw files acquired in data-dependent mode were directly imported into Skyline v1.3 and MS1 precursor ions extracted for all peptides present in the MS/MS spectral libraries. Quantitative analysis is based on extracted ion chromatograms (XICs) and resulting precursor ion peak areas for each peptide M, M + 1, and M + 2, the first, second, and third isotope peak of the isotopic envelope. ANOVA Test was performed to analyze the data and to define if protein quantity in irradiated and nonirradiated samples was significant different. Only quantitative data exhibiting a *P* < 0.05 and a FC ≥ 1.25 or FC ≤ 0.8 were considered as biologically significant.

### Statistical analysis

For preparing data graphs and for statistical analysis, the software Graph Pad Prism (version 5.00; GraphPad Software, La Jolla, California, USA) was used, using a paired *t*-test with confidence interval 95% (*P* < 0.05).

## Results

### Recovery and proliferation

**Figure 1:**
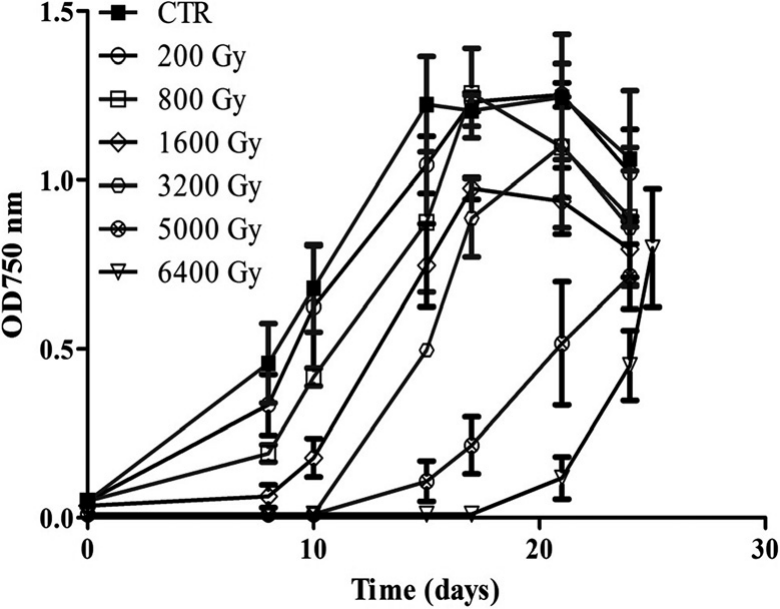
Growth curves of *Arthrospira* sp. PCC8005 following exposure to different doses of gamma rays. Data represent mean of three independent biological replicates (*n* = 3), and error bars present the standard error of the mean (SEM) (Table S1 reports the specific growth rate for each dose).

In order to assess the ability of *Arthrospira* to recover after irradiation, the cells were allowed to regrow at the optimal conditions for photosynthetic growth (Fig.1 and Table S1). The growth curves showed that *Arthrospira sp*. PCC 8005 was able to regrow normally after exposure to 200 Gy until 1600 Gy. Regrowth was also observed after 3200, 5000, and 6400 Gy of gamma radiation, although with a significant delay in time, up to 10–17 days (Table S1). With an increasing dose, the delay in post-irradiation growth increased.

### Functionality of PSII system: quantum yield

**Figure 2:**
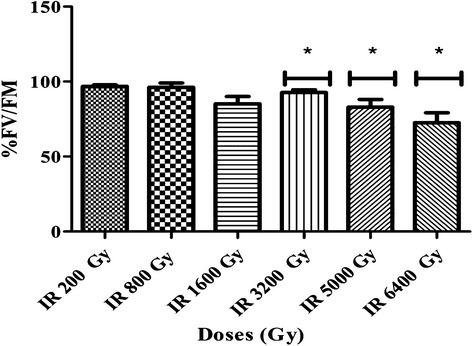
PSII quantum yield of *Arthrospira* sp. PCC8005 after gamma irradiation. The data obtained for the irradiated samples were normalized against and are shown as percentage of their representative non-irradiated control (which was put at 100%). Data represent mean of three in dependent cultures (*n* = 3) and error bars present the standard error of the mean (SEM). An asterisk indicates a value for the irradiated sample which is significant (*P* < 0.05) lower than the value of the representative non-irradiated control culture.

To allow post-irradiation cell proliferation, cyanobacteria need to harvest light via the antenna on their membranes and generate cellular energy through photosynthesis, via photosystem II (PSII). Therefore, the functionality of PSII of *Arthrospira* was assessed immediately after irradiation by measuring chlorophyll a (Chl*a*) and phycobilisome fluorescence, to determine the PSII quantum yield *F*_V_/*F*_M_. Healthy *Arthrospira* cells normally have a yield *F*_V_/*F*_M_ of ca. 0.6 (Masojídek et al. 2010), as was indeed measured for the nonirradiated control cultures, while cells without photosynthetic activity would have a yield *F*_V_/*F*_M_ of 0.0. The photosynthetic yield of the cells exposed to 200 Gy till 1600 Gy of gamma rays was not significantly affected by irradiation. Cells exposed to 3200, 5000, and 6400 Gy, showed a significant decrease of *F*_V_/*F*_M_ to 0.5 or 0.4 (Fig.2). These were also the cultures that displayed a delay in photosynthetic growth after irradiation.

### Pigment content

**Figure 3:**
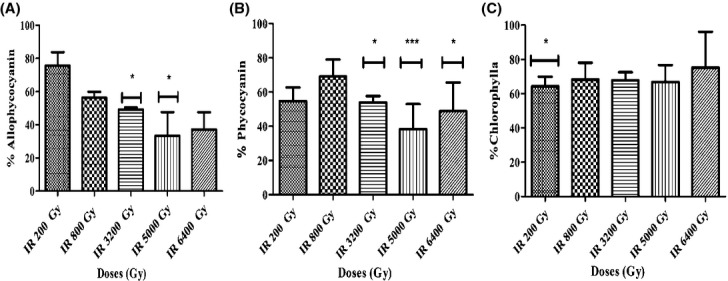
Significant reduction in light harvesting antenna pigments (allophycocyanine and phycocyanine) while stable chlorophyll a pigment content of *Arthrospira* after irradiation. (A) Allophycocyanin content, (B) phycocyanin content, and (C) chlorophyll *a* content. The data obtained for the irradiated samples were normalized against and are shown as percentage of their representative non-irradiated control (which was put at 100%). Data represent mean of three independent biological replicates, and error bars present the standard error of the mean (SEM). One asterisk indicates a value which is significant (*P* < 0.05) different from the value of the non-irradiated control. Three asterisk indicate a value which is highly significant (*P* < 0.001).

To enable photosynthesis, cyanobacteria have large light-harvesting antennas on their membranes (Mullineaux et al. 1997). These antennas, also called phycobilisomes, are protein complexes which contain the photoactive pigments allophycocyanin and phycocyanin (Johnson et al. 1988). Phycobilisomes harvest and transmit the energy to PSII reaction center containing chlorophyll (Chl*a*) (Campbell et al. 1998). Here, the pigment content of *Arthrospira* sp. PCC 8005 was analyzed immediately after irradiation. Data showed a significant decrease in allo-phycocyanin and phycocyanin after exposure to doses of 3200 Gy or higher (Fig.3A and B). No significant change occurred in the overall chlorophyll content (Fig.3C). This reduction in light harvesting pigments coordinate well with our findings of PSII quantum yield that was also reduced at doses of 3200 Gy or higher.

### Photosynthesis and energy production

RNA analysis showed a major decrease in the expression of all components of the photosynthetic apparatus, which likely resulted in the impaired photosynthesis and proliferation after 3200 and 5000 Gy irradiation discussed above. Several genes coding for phycobilisome pigment biosynthesis (*cpc* and *apc* genes) were downregulated and one gene specific for phycobilisome degradation was activated (*nblA2*) (Tables2). Similarly, transcription of chlorophyll A pigment biosynthesis (*chl* genes) and PSII biosynthesis (*psb* genes) was reduced, while transcription of proteases for degradation of PSII-D1 proteins (*ftsH*) was induced (Tables1 and 2). Likewise, the significant upregulation of the *psbI* gene was seen. The exact role of PsbI in the assembly of PSII is unclear, nevertheless it has been shown that the loss of PsbI led to a dramatic destabilization of CP43 (PsbC*),* a core antenna protein of photosystem II, and suggest that PsbI might contribute to an early assembly partner for D1 protein (PsbA) (Dobakova et al. 2007). In addition, the transcription of several genes involved in electron transfer from PSII to PSI, such as the *ndh* plastoquinone genes, *cyd* and *cox* cytochrome genes, and heme biosynthesis genes (*hem*), were reduced (Tables1 and 2). Likewise, the transcription of several genes encoding the structural subunits of photosystem I (*psa* genes) was repressed (Table1). Most of the genes involved in production and conversion of energy obtained from photosynthesis, such as the ferredoxin (FD) gene (*petF*), the ferredoxin:NADP^+^ oxidoreductase (FNR) gene (*petH*), and the ATP synthase-coding operon (*atp* genes), showed a reduced expression (Table1).

**Table 1:** Transcriptomic (microarray) results for genes known to be involved in photosynthesis

Photosynthesis	Accession number	Gene	Protein function	Fold change 3200 Gy	Fold change 5000 Gy
PSII	ARTHROv5_10312	*psbA1*	Photosystem II reaction center D1 protein Q(B)	NS	NS
	ARTHROv5_10319	*psbA2*	Photosystem II reaction center D1 protein Q(B)	NS	NS
	ARTHROv5_40241	*psbA3*	Photosystem II reaction center D1 protein Q(B)	0.31	0.39
	ARTHROv5_60197	*psbA4*	Photosystem II reaction center D1 protein Q(B)	NS	NS
	ARTHROv5_10245	*psbB*	Photosystem II P680 chlorophyll A apoprotein (CP47 protein)	0.57	0.27
	ARTHROv5_11994	*psbC*	Photosystem II CP43 protein	NS	0.58
	ARTHROv5_11993	*psbD1*	Photosystem II reaction center D2 protein Q(A)	NS	NS
	ARTHROv5_60553	*psbE*	Photosystem II reaction center subunit V (Cytochrome b559 subunit alpha)	NS	0.53
	ARTHROv5_60554	*psbF*	Photosystem II reaction center subunit VI (Cytochrome b559, subunit beta)	0.58	0.35
	ARTHROv5_60555	*psbL*	Photosystem II reaction center protein L	0.53	0.32
	ARTHROv5_40752	*psbH*	Photosystem II reaction center protein H (PSII-H)	NS	0.62
	ARTHROv5_40753	*psbN*	Photosystem II reaction center protein N (PSII-N)	NS	NS
	ARTHROv5_30303	*psbI*	Photosystem II reaction center protein I	1.63	4.40
	ARTHROv5_11112	*psb28*	Photosystem II reaction center protein W (13 kDa protein)	NS	NS
	ARTHROv5_61163	*psb28*	Photosystem II reaction center psb28-like protein	NS	1.95
	ARTHROv5_20093	*psb27*	Photosystem II 11 kD protein	NS	0.51
	ARTHROv5_40153	*psbO*	Photosystem II Mn-stabilizing polypeptide precursor (MSP)	NS	0.23
	ARTHROv5_10852	*psbP*	Photosystem II oxygen-evolving complex 23K protein	NS	NS
	ARTHROv5_40969	*psbU*	Photosystem II extrinsic protein precursor (12 kDa protein)	NS	NS
	ARTHROv5_50093		Putative cytochrome c-550-like protein precursor	0.31	0.38
	ARTHROv5_50094	*psbV*	Cytochrome c-550 precursor	0.30	0.28
	ARTHROv5_61031	*psbY*	Photosystem II protein Y	NS	0.46
	ARTHROv5_11396	*psbZ*	Photosystem II reaction center protein Z (PSII-Z)	NS	NS
	ARTHROv5_60878	*ftsH*	(ATP-dependent zinc-metallo protease)	1.27	2.77
	ARTHROv5_61180	*isiA*	Iron stress-induced chlorophyll-binding protein (CP43′)	4.98	NS
Plastoquinone (PQ)	ARTHROv5_10689	*ndhA*	NAD(P)H-quinone oxidoreductase, membrane subunit H	0.71	0.29
	ARTHROv5_10690	*ndhI*	NAD(P)H-quinone oxidoreductase, subunit I	NS	0.19
	ARTHROv5_10691	*ndhG*	NAD(P)H-quinone oxidoreductase, chain 6	NS	0.42
	ARTHROv5_10692		conserved protein of unknown function	0.37	0.30
	ARTHROv5_10693	*ndhE*	NAD(P)H-quinone oxidoreductase, membrane subunit K	NS	0.31
	ARTHROv5_40057	*ndhH*	NAD(P)H-quinone oxidoreductase, chain H	0.57	0.45
	ARTHROv5_40540	*ndhD1*	NAD(P)H-quinone oxidoreductase chain 4	NS	0.53
	ARTHROv5_40541	*ndhF1*	NAD(P)H-quinone oxidoreductase, chain 5	NS	0.42
	ARTHROv5_40542	*ndhD2*	NAD(P)H-quinone oxidoreductase, chain 4	NS	0.35
	ARTHROv5_60388	*ndhF2*	NAD(P)H-quinone oxidoreductase, chain 5	NS	0.38
	ARTHROv5_60389	*ndhD3*	NAD(P)H-quinone oxidoreductase, chain 4	NS	0.35
	ARTHROv5_60547	*ndhJ*	NAD(P)H-quinone oxidoreductase, subunit J	NS	0.41
	ARTHROv5_60548	*ndhK*	NAD(P)H-quinone oxidoreductase, subunit K	NS	0.50
	ARTHROv5_60549	*ndhC*	NAD(P)H-quinone oxidoreductase, chain A	NS	0.49
	ARTHROv5_60715	*ndhD4*	NAD(P)H-quinone oxidoreductase, chain 4	0.53	0.17
	ARTHROv5_60716	*ndhF*	NAD(P)H-quinone oxidoreductase, subunit F	NS	0.21
Cytochrome b6f	ARTHROv5_40397	*cyoE*	Protoheme IX farnesyltransferase (heme O synthase)	NS	0.32
	ARTHROv5_40398		Cytochrome oxidase assembly protein	NS	0.29
	ARTHROv5_40399	*coxB*	Cytochrome c oxidase subunit II	NS	0.54
	ARTHROv5_40400	*coxA*	Cytochrome c oxidase subunit I	NS	0.41
	ARTHROv5_40401	*coxC*	Cytochrome c oxidase subunit III	NS	0.32
	ARTHROv5_61103	*cydB*	Cytochrome bd ubiquinol oxidase, subunit II	NS	0.47
	ARTHROv5_60566	*ccsB*	Cytochrome c biogenesis protein	NS	NS
	ARTHROv5_60567		Cytochrome c biogenesis protein transmembrane region	NS	NS
	ARTHROv5_60740	*ccsA*	Cytochrome c biogenesis protein	NS	0.45
	ARTHROv5_50134		Cytochrome c, monohaem	NS	0.46
	ARTHROv5_40277	*petJ*	Cytochrome c6 (Soluble cytochrome f) (Cytochrome c553)	0.35	0.33
	ARTHROv5_40850	*petA*	Cytochrome f	NS	NS
Plastocyanin (PC)	ARTHROv5_40851	*petC*	Cytochrome b6-f complex iron-sulfur subunit 1 plastocyanin oxidoreductase	NS	NS
PSI	ARTHROv5_10984	*psaA*	Photosystem I P700 chlorophyll a apoprotein A1	0.56	0.27
	ARTHROv5_10985	*psaB*	Photosystem I P700 chlorophyll a apoprotein A2	0.59	0.28
	ARTHROv5_10235	*psaC*	Photosystem I reaction center subunit VII, iron-sulfur center	0.54	0.37
	ARTHROv5_30080	*psaD*	Photosystem I reaction center subunit II (16 kDa polypeptide)	NS	NS
	ARTHROv5_30570	*psaE*	Photosystem I reaction center subunit IV	2.12	2.21
	ARTHROv5_30656	*psaJ*	Photosystem I reaction center subunit IX	0.29	0.14
	ARTHROv5_30657	*psaF*	Photosystem I reaction center subunit III precursor	0.35	0.15
	ARTHROv5_50157	*psaL*	Photosystem I reaction center subunit V	0.50	0.24
	ARTHROv5_40172	*psaX*	Photosystem I reaction center subunit	0.31	0.27
	ARTHROv5_11973	*btpA*	Photosystem I biogenesis protein	NS	0.36
	ARTHROv5_11992	*ycf4*	Photosystem I assembly protein	NS	NS
	ARTHROv5_41247	*ycf3*	Photosystem I assembly protein	NS	NS
Ferrodoxin (FD)	ARTHROv5_60106	*petF1*	Ferredoxin (2Fe-2S)	0.59	0.29
	ARTHROv5_10430	*petF2*	Ferredoxin-2	0.27	0.18
FNR	ARTHROv5_41386	*petH*	Ferredoxin:NADPH reductase	NS	0.57
	ARTHROv5_50074	*iscA1*	FeS cluster assembly protein	0.51	0.58
	ARTHROv5_60637	*iscA2*	FeS cluster assembly protein, IscA-like	NS	NS
	ARTHROv5_11765	*sufR*	Iron-sulfur cluster biosynthesis transcriptional regulator	NS	2.12
ATP synthesis	ARTHROv5_60530	*atpI*	ATP synthase protein I	0.33	0.18
	ARTHROv5_60531	*atpB*	ATP synthase A chain (ATPase protein 6)	0.35	0.17
	ARTHROv5_60532	*atpE*	ATP synthase C chain, membrane-bound, F0 sector	0.24	0.18
	ARTHROv5_60533	*atpG2*	ATP synthase B chain (Subunit II)	0.07	0.14
	ARTHROv5_60534	*atpF*	ATP synthase B chain (Subunit I)	0.07	0.17
	ARTHROv5_60535	*atpH*	ATP synthase D chain; ATP synthase F1	0.12	0.15
	ARTHROv5_60536	*atpA*	ATP synthase, alpha subunit	0.23	0.17
	ARTHROv5_60537	*atpG1*	ATP synthase, gamma subunit	0.33	0.33
	ARTHROv5_12000	*atpC*	ATP synthase epsilon chain	NS	NS
	ARTHROv5_12001	*atpD*	ATP synthase, beta subunit	NS	NS

The fold change (FC) values listed are values for which *P* < 0.05, and are only considered biologically significant if FC > 2 or <0.5. “NS” stands for statistically not significant differentially expressed (*P* ≥ 0.05).

**Table 2:** Transcriptomic (microarray) results for genes known to be involved in pigment biosynthesis and degradation

Pigment biosynthesis	Accession number	Gene	Protein function	Fold change 3200 Gy	Fold change 5000 Gy
C-phycocyanin	ARTHROv5_11553	*cpcB*	C-phycocyanin beta subunit	NS	NS
	ARTHROv5_11554	*cpcA*	C-phycocyanin alpha subunit	NS	NS
	ARTHROv5_11555	*cpcC1*	Phycobilisome linker polypeptide, phycocyanin-associated, rod 1	NS	NS
	ARTHROv5_11556	*cpcC2*	Phycobilisome linker polypeptide, phycocyanin-associated, rod 2	NS	0.62
	ARTHROv5_11557	*cpcD*	Phycobilisome linker polypeptide, phycocyanin-associated, rod-capping	NS	0.64
	ARTHROv5_11558	*cpcE*	Phycocyanin alpha subunit phycocyanobilin lyase	NS	NS
	ARTHROv5_11559	*cpcF*	Phycocyanin alpha-subunit phycocyanobilin lyase	NS	NS
	ARTHROv5_40726	*cpcG*	Phycobilisome rod-core linker protein	0.56	0.32
	ARTHROv5_60720	*cpcT*	Chromophore lyase	NS	0.34
	ARTHROv5_11397	*nblB2*	Phycocyanin alpha phycocyanobilin lyase related protein	NS	NS
	ARTHROv5_50028	*nblB1*	Phycocyanin alpha phycocyanobilin lyase related protein	1.86	1.80
	ARTHROv5_61056	*nblA1*	Phycobilisome degradation protein	NS	NS
	ARTHROv5_61095	*nblA2*	Phycobilisome degradation protein	NS	2.17
Allo-phycocyanin	ARTHROv5_10637	*apcA*	Allophycocyanin alpha subunit	0.59	0.19
	ARTHROv5_10636	*apcB*	Allophycocyanin beta subunit	0.27	0.13
	ARTHROv5_10635	*apcC*	Phycobilisome rod-core linker protein	0.29	0.16
	ARTHROv5_60056	*apcD*	Allophycocyanin alpha subunit	NS	0.53
	ARTHROv5_61214	*apcE*	Phycobiliprotein	NS	0.51
	ARTHROv5_12132	*apcF*	Allophycocyanin beta subunit	0.38	0.16
Chlorophyll	ARTHROv5_30766	*chlG*	Chlorophyll a synthase	NS	0.28
	ARTHROv5_30670	*por*	chlorophyll synthase/NADPH-protochlorophyllide oxidoreductase	NS	0.31
	ARTHROv5_41143	*chlL*	Light-independent protochlorophyllide reductase	0.35	0.22
	ARTHROv5_40946	*acsF*	Aerobic Mg-protoporphyrin IX monomethyl ester	NS	0.32
	ARTHROv5_11499	*chlH*	Mg chelatase H subunit	NS	0.22
	ARTHROv5_40768	*bchD*	Mg-protoporphyrin IX chelatase, subunit D	NS	0.14
	ARTHROv5_61176	*bchI*	(Mg-protoporphyrin IX chelatase (38 kDa subunit)	NS	NS
	ARTHROv5_60718		GUN4 domain protein	NS	0.36
	ARTHROv5_11688		putative GUN4-like regulator	0.28	0.39
Carotenoid	ARTHROv5_10189		putative Acyl-CoA dehydrogenase	0.40	0.30
	ARTHROv5_10200		conserved hypothetical protein	0.41	0.26
	ARTHROv5_10201		conserved hypothetical protein	NS	0.31
	ARTHROv5_10202		putative Beta-carotene ketolase	0.36	0.18
Isoprenoid	ARTHROv5_40094	*ispD1*	4-diphosphocytidyl-2-C-methyl-d-erythritol synthase	NS	0.46
	ARTHROv5_11117	*ispE*	4-diphosphocytidyl-2-C-methyl- d-erythritol kinase	NS	0.48
	ARTHROv5_20267	*ispF*	2-C-methyl- d-erythritol 2,4-cyclodiphosphate synthase	NS	0.46
	ARTHROv5_30478	*ispD*	2-C-methyl- d-erythritol 4-phosphate cytidylyl transferase	NS	0.22
	ARTHROv5_30479		hypothetical protein	0.11	0.13
	ARTHROv5_60585	*ispG*	1-hydroxy-2-methyl-2-(E)-butenyl 4-diphosphate synthase	0.22	0.17
Heme	ARTHROv5_50123	*hemC*	Porphobilinogen deaminase	0.41	0.38
	ARTHROv5_60626	*hemE*	Uroporphyrinogen decarboxylase	0.26	0.29
	ARTHROv5_11660	*hemF*	Coproporphyrinogen III oxidase	0.45	0.35
	ARTHROv5_10139	*hemG*	Protoporphyrinogen oxidase	NS	0.27
	ARTHROv5_50161	*hemH*	Ferrochelatase	1.65	1.5
	ARTHROv5_30029	*hemL*	Glutamate-1-semialdehyde aminotransferase (aminomutase)	0.29	0.12

The fold change (FC) values listed are only values for which *P* < 0.05, and are only considered biologically significant if FC > 2 or < 0.5. “NS” stands for statistically not significant differentially expressed (*P* ≥ 0.05).

### Photosensing and cell motility

High doses of gamma radiation induced strongly the transcription of several genes coding for “chromophore” proteins, that is, photoreceptor pigments involved in photosensing and signaling for the regulation of cyanobacterial phototaxis (Table S2). First, the biosynthesis of tetrahydrobiopterin (BH4)-containing proteins, that is, pterin-like chromophores, was induced. Tetrahydrobiopterinis biosynthesized from guanosine triphosphate (GTP) by three chemical reactions mediated by the enzymes GTP cyclohydrolase I (GTPCH) (*folE1* gene), 6-pyruvoyltetrahydropterin synthase (PTPS) (*ygcM*), and sepiapterin reductase (SR) (Woo et al. 2002). Second, also the gene coding for the Cryptochrome-DASH protein (*cry* gene), which is a flavoprotein-type chromophore, was upregulated. In addition, also several other genes coding for response regulatory proteins with photosensor domains (GAF, PAS), as well as the associated signal transduction and regulatory systems (i.e., histidine kinases and transcriptional regulators), and the genes for synthesis of the secondary messenger cyclic diguanylate (c-di-GMP) were induced. These are all systems typically involved in cell motility regulation. Based on COG enrichment analysis, the category of cell motility (N) proteins indeed showed a significant altered expression after irradiation. However, the expression of genes involved in swimming motility (pilin genes) and floating motility (gvp gas vacuole genes) was reduced by radiation.

### Carbon fixation and secondary metabolite biosynthesis

The transcription analysis of genes related to carbon fixation showed a general repression after irradiation. The genes encoding the carbon dioxide fixation mechanism, that is, the carboxysome (*ccm* and *cch* genes), and the genes related to (Calvin–Benson–Bassham (CBB) cycle) (*cbb, gpm, gap* and *glp* genes) were repressed (Table3).

**Table 3:** Transcriptomic (microarray) results for genes known to be involved in Carbon metabolism

CO_2_ fixation	Accession number	*Gene*	Function	Fold Change 3200 Gy	Fold change 5000 Gy
Carboxysome	ARTHROv5_60383		Putative carbon dioxide concentrating mechanism protein	NS	0.57
	ARTHROv5_60384	*ccmM*	Carbon dioxide concentrating mechanism protein	0.21	0.08
	ARTHROv5_60385	*cchB*	Putative carboxysome-like ethanolaminosome structural protein, ethanolamine utilization protein	0.32	0.12
	ARTHROv5_60386	*ccmK1*	Carbon dioxide-concentrating mechanism protein	0.18	0.08
	ARTHROv5_60387	*cchA*	Putative carboxysome-like ethanolaminosome structural protein, ethanolamine utilization protein	0.34	0.15
	ARTHROv5_60714		CO_2_ hydration protein	0.44	0.16
	ARTHROv5_61007	*ccmK1*	Carboxysome shell protein	NS	0.21
	ARTHROv5_61008	*ccmK2*	Carboxysome shell protein	NS	0.19
RuBisCO	ARTHROv5_50349	*cbbS*	Ribulose bisphosphate carboxylase (RuBisCO), small subunit	0.19	0.08
	ARTHROv5_50350	*rbcX*	Chaperonin family protein	0.32	0.17
	ARTHROv5_50351	*cbbL*	Ribulose bisphosphate carboxylase (RuBisCO), large subunit	0.15	0.07
	ARTHROv5_50352	*cbbR1*	Ribulose bisphosphate carboxylase (RuBisCO), operon transcriptional regulator	0.42	0.35
	ARTHROv5_50129	*rca*	Ribulose bisphosphate carboxylase/oxygenase activase	NS	NS
	ARTHROv5_10999	*cbbR2*	Putative RuBisCO transcriptional regulator, RbcR-like	0.45	0.43
	ARTHROv5_10998	*cbbR3*	Putative RuBisCO transcriptional regulator, RbcR-like	0.39	0.41
Calvin cycle	ARTHROv5_10997	*spkF*	Ser/Thr protein kinase	NS	0.36
	ARTHROv5_20037	*pgk*	Phosphoglycerate kinase	NS	0.47
	ARTHROv5_60907	*gpmB2*	Phosphoglycerate mutase	NS	0.34
	ARTHROv5_30667	*gpmB1*	Phosphoglycerate mutase	0.46	0.21
	ARTHROv5_30574	*gpmI*	2,3-bisphosphoglycerate-independent phosphoglycerate mutase	NS	0.38
	ARTHROv5_11456	*gap1*	Glyceraldehyde-3-phosphate dehydrogenase 1	NS	0.31
	ARTHROv5_30613	*gap2*	Glyceraldehyde-3-phosphate dehydrogenase 2	0.53	0.25
	ARTHROv5_41419	*xfp*	d-xylulose 5-phosphate/d-fructose 6-phosphate phosphoketolase	0.46	0.35
	ARTHROv5_20113	*gnd*	Gluconate-6-phosphate dehydrogenase, decarboxylating	0.44	0.36
	ARTHROv5_10443	*pgi*	Glucose-6-phosphate isomerase	0.45	0.31
	ARTHROv5_30212	*pfkB1*	Fructokinase	0.58	0.31
	ARTHROv5_10198	*pfkB2*	Putative pfkB family carbohydrate kinase; Adenosine kinase	0.67	0.50
	ARTHROv5_40143		Putative ribulose-5-phosphate 4-epimerase and related epimerase and aldolases	0.40	0.25
Glycogen biosynthesis	ARTHROv5_41216	*glgA1*	Glycogen synthase	0.31	0.23
	ARTHROv5_60979	*glgA2*	Glycogen synthase	0.59	0.59
	ARTHROv5_61087	*glgX2*	Glycogen debranching enzyme	NS	0.24

The fold change (FC) values listed are values for which *P* < 0.05, and are only considered biologically significant if FC > 2 or <0.5. “NS” stands for not statistically significant differentially expressed (*P* ≥ 0.05).

In line with reduced carbon capture, also genes involved in biosynthesis of intracellular carbon storage compounds such as glycogen (*glg* genes), and lipids such as fatty acids – including gamma-linolenic acid (GLA) synthesis – (*fab* and *des* genes), or intracellular solutes with a role in salt tolerance (*stpA* and *ggpS* genes) was transcriptionally reduced (Table S3). Also genes involved in extracellular metabolite production, such as biosynthesis of the extracellular cyclic peptide pattelamide A (*pat* genes) were reduced in expression (Table S3).

### Stress response and antioxidants

It is well-documented that cyanobacteria developed various antioxidant defenses mechanism to cope with ROS damage, involving enzymatic and nonenzymatic ways (Latifi et al. 2009). Remarkably, the gene coding for the antioxidant enzyme catalase is absent in the *Arthrospira* sp. PCC8005 genome, and the expression level of the gene coding for the antioxidant enzyme superoxide dismutase (*sodB*) was not significantly induced (even slightly reduced) (Table S4). Other known nonenzymatic antioxidants, are the pigments C-phycocyanin and *β*-carotene, but the genes involved in the synthesis of those compounds was downregulated, as explained above (Table2). In contrast, transcriptome data showed in *Arthrospira* sp. PCC8005 mainly the upregulation of the thiol-based antioxidant systems after irradiation, including glutathione (GSH), thioredoxin, and peroxiredoxin systems (Table S4). Several genes coding for GSH synthesis and regeneration (i.e., reduction) were upregulated (*gshB* GSH synthase, lactoylglutathione lyase, and glutathionylspermidine synthase). Meanwhile the expression level of the glutaredoxin gene (*gor*), which is involved in GSH oxidation, was reduced. Similarly, expression of genes coding for thioredoxin was reduced, while the thioredoxin-reductase gene (*trxB*) for thioredoxin-regeneration (i.e., reduction) was induced. Moreover, our findings showed a significant elevation in the transcription level of gene coding for peroxiredoxin. It is reported that transcription of several key genes coding for proteins involved in redox homoeostasis, such as glutaredoxin and a number of thioredoxins, is regulated by the Fur transcriptional regulator (Fernandes et al. 2005) whose expression was also found induced in *Arthrospira* sp. PCC8005 (*fur*) after irradiation. Also other Fur regulated genes involved in iron homeostasis, such as the iron stress inducible *isiA* gene and the bacterioferritin genes, were differentially expressed.

### Protein damage and recycling

Irradiation-induced expression of genes encoding heat shock proteins (HSP), known as molecular chaperones. This group is a class of functionally related proteins involved in the folding and unfolding of other proteins (Table S5). *Arthrospira* sp. PCC 8005 contains five copies of the HSP70-type *dnaK* gene and the transcription level of three of them (*dnaK*1, *dnaK*4 and *dnaK*5) was significantly induced. Likewise, also the upregulation of *dnaJ* and *cbp*A (a *dnaJ* homologue) genes, which act synergistically with *dnaK*, was observed. The g*roL1* and *groL2* genes*,* coding for the large subunit of the HSP60-type GroESL, were also found upregulated. However, *groS*, which generally act as a co-chaperone of GroEL, did not show a significant expression change. In addition, a set of protease and peptidase genes (e.g., HSP100-type *ClpS2*) involved in the proteolytic degradation and removal of proteins that are damaged beyond repair, were upregulated after IR (Table S5). And as mentioned before, also the transcription of some very target-specific proteases such as *nblA* and *ftsH*, involved in controlled phycobilisome or PSII-reaction center D1 protein degradation, were increased.

### DNA repair and genetic modifications

Many of the differentially expressed genes in irradiated cells were involved in repair DNA damage (Table S6). A few genes belonging to the dsDNA damage repair pathways such *recJ*, *recQ*, *recG*, *holB*, and *gyrA* were differentially expressed. However, the *recA* gene, which a key protein for DNA repair in bacteria, such as *D. radiodurans* (Daly 2009)*,* was not differential expressed. Surprisingly, the gene for the related LexA repressor that works together with RecA and activates the transcription of the SOS response system, is absent in *Arthrospira* sp. PCC8005 genome. Microarray data did reveal differential expression of many genes involved in ssDNA-damage repair, either via nucleotide excision repair (NER), such as *uvr* genes, or either via the ssDNA mismatch repair system (MMR), such as the *mut* genes. And nonrepaired nucleotides were potentially removed by nudix hydrolase, which also displayed an upregulation pattern.

Additionally, an interesting group of genes coding for Type I site specific deoxyribonucleases was highly upregulated. Some of those genes showed high homology with the R, S, and M subunit of the Type I restriction modification system (RM) of *Arthrospira platensis* NIES39. Restriction-Modification enzymes are used by many organisms to protect themselves against foreign DNA (Wilson and Murray 1991), and the role of RM systems in *A. platensis* has been discussed (Fujisawa et al. 2010). Type I restriction modification systems are composed of three subunits, encoded by three *hsd* genes: the *hsdR* gene required for restriction activity; the *hsdS* gene responsible for DNA specificity, and the *hsdM* gene which is required for methylation activity. These enzymes add a methyl group to a DNA molecule at a specific site to protect the site from restriction endonuclease cleavage, and thus from DNA damage.

Irradiation induced also a set of genes potentially involved in a toxin/antitoxin system and a number of mobile genetic elements (MGEs) (Tables S7-S8-S9). In total, 37 genes coding for transposases (jumping genes) displayed an upregulation pattern (Table S7). Moreover, *Arthrospira* sp. PCC8005 contains 14 regions of phage immunity (CRISPR) genes (*cas* genes) of which 1 crisper region showed clear increase and 7 phage-like genomic islands (*fax* genes) (Table S8), of which 1 copy and 7 copies respectively exhibited a clear increase in their transcripts level after IR. This is the first observation that this genomic element is responding to an exogenous stress in the cells environment, and thus might still be functional.

### Differentially expressed conserved hypothetical proteins

The genes coding for conserved hypothetical proteins (Table S10) with unassigned function (COG: N/A) were the most abundant among all differentially expressed genes. A cluster of seven genes named *arh* (ARTHROv5_10466 to ARTHROv5_10472), showed a very high expression in a dose-dependent response to IR (Table4). A number of these genes were in fact not only found to be highly upregulated at RNA level but also at protein level (Tables4, S11, and S12). Three of the Arh proteins displayed high abundance after exposure to 3200 and 5000 Gy. It is well known that the overlap between mRNA transcript and proteomics is minor, nevertheless proteomic results showed a clear correlation with respect to this new set of proteins. This is peculiar, as overall the proteomics analysis revealed only few differentially expressed proteins, that is, 31 and 36 respectively after 3200 and 5000 Gy, and with little similarity between 3200 and 5000 Gy (Table S10 and S11).

**Table 4:** Transcriptomic and proteomic results for conserved hypothetical proteins, specifically expressed in response to ionizing radiation

Accession number	Gene	Protein function	COG	RNA fold change 3200 Gy	RNA fold change 5000 Gy	Protein fold change 3200 Gy	Protein fold change 5000 Gy
ARTHROv5_10472	*arhA*	Putative ABC-type phosphate transport system, periplasmic component, PstS-like	**P**	14.58	9.24	ND	ND
ARTHROv5_10471	***arhB***	**Conserved protein of unknown function**	**D**	**NS**	**22**.**35**	**1**.**75**	**NS**
ARTHROv5_10470	***arhC***	**Conserved protein of unknown function** (conserved domain involved in chromosome segregation)	**L**	**3**.**13**	**14**.**62**	**1.40**	**NS**
ARTHROv5_10469	*arhD*	Conserved hypothetical protein	**–**	5.43	9.46	ND	ND
ARTHROv5_10468	***arhE***	**Conserved protein of unknown function**	**–**	**9**.**58**	**11**.**98**	**7**.**12**	**4.30**
ARTHROv5_10467	*arhF*	Conserved hypothetical protein	**–**	5.10	5.75	ND	ND
ARTHROv5_10466	*arhG*	Transcriptional regulator, XRE family	**K**	2.55	2.78	ND	ND

The fold change (FC) values listed are values for which *P* < 0.05, and are only considered biologically significant if FC > 2 or <0.5 for microarray data, FC > 1.25 or <0.8 for proteomics data. “NS” stands for statistically not significant differentially expressed (*P* ≥ 0.05) as RNA or as protein. “ND” stands for not detected as protein. Bold are genes of which the proteins were also detected in Proteomics.

Advanced bio-informatics analysis was carried out to assess the conservation and potential function of these proteins. Two proteins, ArhB and ArhC, were conserved within the cyanobacteria phylum and showed similarities in amino acids sequences between species. *Arthrospira* species showed the highest amino acids sequence similarity ranging from 95.12%, 94.82%, and 94.21% respectively for *A. platensis* C1, *Arthrospira maxima* CS328, and *A. platensis* NIES-39. Less similarity was seen with members of the *Nostocacea* family such as *N. punctiforme* PCC 73102 (49.38%), *Anabeana variabilis* ATCC 29413 (48.46%), and *Nostoc* sp. PCC 7120 (47.84%). Interestingly, the comparison of the two proteins with the *Deinococcacae* family, demonstrate sequence homology with *D. radiodurans* R1 species. In addition, the analysis of conserved functional domain, showed only a conserved domain involved in chromosome segregation for the ArhC protein.

## Discussion

*Arthrospira sp*. PCC 8005 cells showed photosynthetic recovery and proliferation after all doses of ^60^Co gamma radiation (dose rate of 527 Gy h^−1^) tested, up to a total absorbed dose of 6400 Gy. This classifies this bacterium as “radiation resistant” (Bauermeister et al. 2011; Luan et al. 2014).

In general, for cyanobacteria, many studies focused on the tolerance to photosynthetically active radiation (PAR) and ultra violet (UV) radiation (photons in the wavelength range of 700 to 400 nm, corresponding to photon energies from 1.5 to 3 eV; and photons of 400 nm to 1 nm with 3 to 1000 eV, repectively), to understand its impact on the growth and biomass yield when they are cultivated, for example, on spirulina farms for diverse biotechnological applications (Wu et al., 2005, Singh et al. 2010b; Rai et al. 2013). *Arthrospira* was indeed also found to be tolerant to high fluxes of VIS and UV (Wu et al. 2005).

Most studies looked at the impact of UV-A (315–400 nm) or UV-B (280–315 nm). Few studies reports the effect of UV-C (100–280 nm) or shorter wavelength (<100 nm) on cyanobacteria, since these wavelengths does not reach the earth surface at present owing to the absorption by ozone and losses through atmospheric scattering. One team investigated the effect of a Martian UV-flux (>200 nm) (e.g., UV-C) on the cyanobacterium *Chrococcidiopsis* (Baque et al. 2013). Only very few studies have investigated the tolerance of cyanobacteria to even more energetic radiation, such as X-rays (photons of 1 to 0.01 nm and 1000–100,000 eV) (Singh et al. 2010a) or gamma-rays (photons < 0.01 nm and >100,000 eV) (Billi et al. 2000; Hasnain 2006). This study is the first, showing that *Arthrospira* is highly tolerant to gamma rays, and can survive at least 6400 Gy (dose rate of 527 Gy h^−1^). A trait similar as the rock-dwelling coccoidal cyanobacterium *Chroococcidiopsis* able to survive X-ray as high as 15,000 Gy (Billi et al. 2000). Likewise, the planktonic filamentous cyanobacterium *Anabaena*, was tolerant up to 5000 Gy of acute ^60^Co gamma radiation (dose rate of 6250 Gy h^−1^) without adverse effect on diazotrophic growth and metabolism (Singh et al. 2010a). In addition, the study of Dartnell et al. (2011) reports the resistance of coccoidal cyanobacterium *Synechocystis sp*. PCC 6803 up to 30,000 Gy ^60^Co gamma radiation (dose rate 10,000 Gy h^−1^).

From an ecological point of view, one could wander why *Arthrospira* is gamma radiation resistant, as in its current natural habitat (soda lakes), it is not exposed to such types or such doses of IR. It is assumed that cyanobacteria have acquired an advanced defense mechanism against radiation since they were exposed to high levels of IR on earth during the Precambrian era (Castenholz and Garcia-Pichel 2002). On an early earth, without complete atmosphere, cyanobacteria were exposed to high intensities of photosynthetic active light PAR or VIS, ultraviolet light UV and other types of electromagnetic waves such as X-rays and gamma-rays. Such high-energetic electromagnetic waves (photons) are strongly penetrating and can damage cells by interacting directly with cellular components (DNA, proteins, and lipids) or indirectly with water molecules producing free radicals leading to cell damage (Le Caer 2011). As such, it is assumed that early on cyanobacteria have developed high-effective mechanism to protect themselves and deal with detrimental effects of radiation (Bebout and Garcia-Pichel 1995), by combining multiple strategies such as avoidance (e.g., moving away), protection (e.g., shielding), detoxification (e.g., antioxidants), and repair (Singh et al. 2010b).

**Figure 4:**
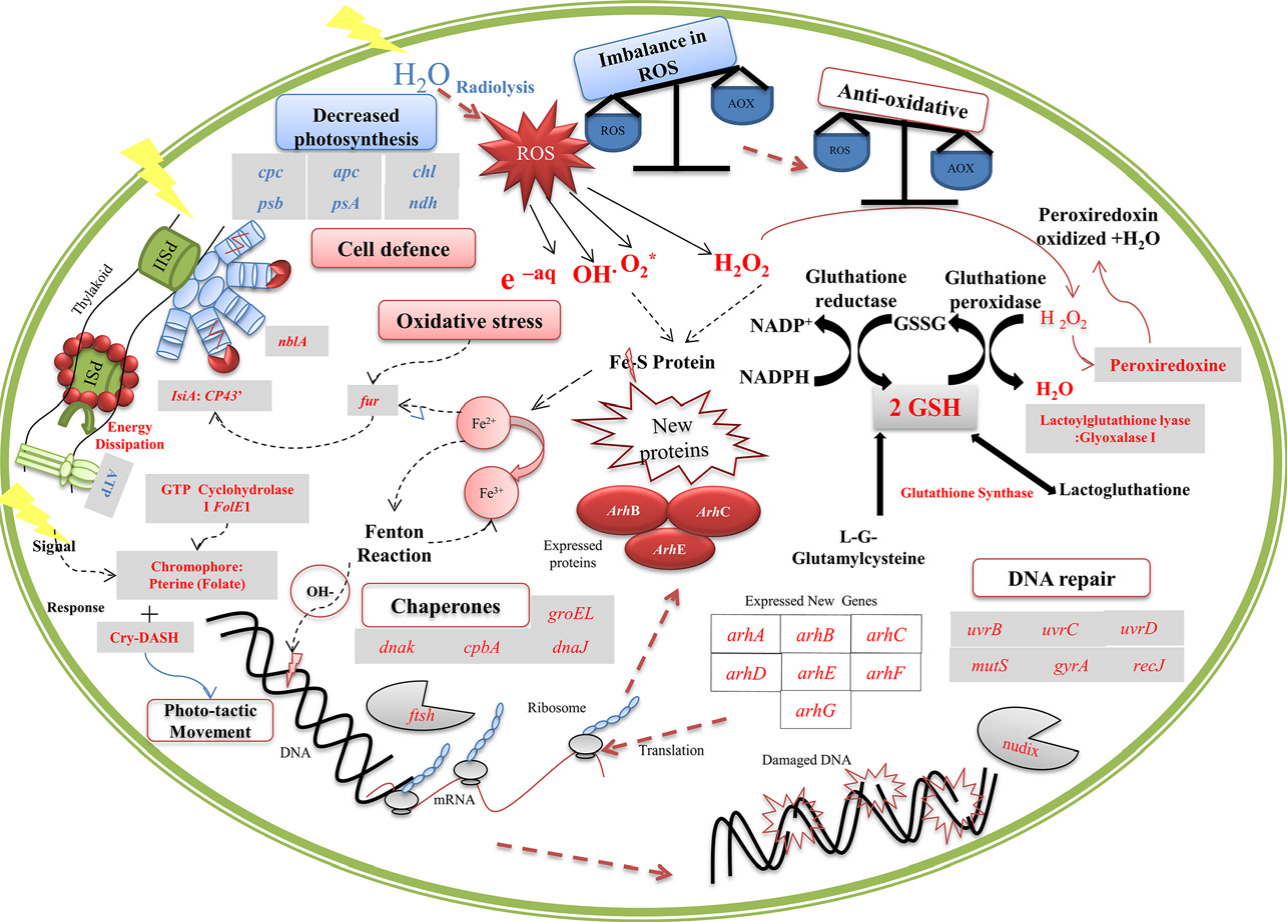
A conceptual model describing the response of *Arthrospira sp*. PCC 8005 to gamma irradiation. The circular diagram represents a cell with the key genes and proteins up regulated after irradiation in Red and the down regulated ones in Bleu. Gamma irradiation leads to ROS production and Redox imbalance. Glutathione molecules are used as key antioxidant molecules for ROS scavenging (the glutathione cycle and the related enzymes involved in glutathione synthesis were highlighted). Also enzymatic antioxidants such as peroxiredoxin are used. ROS causes DNA damage, which activates DNA reparation, involving a number of different genes (*uvr, rec, gyrA, mut, nudix*). Oxidative stress activates the Fur regulon which controls iron homeostasis and enhance *isiA* gene expression for protection of the photosystems PSI and PSII. In parallel, there is shutdown of all genes related to the synthesis of photo-pigments (*cpc, apc, chl*), the degradation of phycobilisomes by protein NblA is activated, and there is a coordinated repression of all the genes required for photosynthesis (*psa, psb, atp*). There is an induction of the photosensor Cry-DASH gene and the biosynthesis pathway of pterine which are involved in signalling and phototactic movement. And there is an activation of several new genes, coding for conserved hypothetical proteins (Arh) in response to gamma rays. ROS, reactive oxygen species.

Nevertheless, so far, no molecular investigations were performed, to understand this extraordinary radiation resistance property of cyanobacteria to such IRs. In this study, a new full genome tilling-array chip, specific for the filamentous *Arthrospira sp*. strain PCC 8005 was designed and constructed, in order to investigate the transcriptomic response of this cyanobacterium to high doses of gamma irradiation (Fig. S2). Overall, a general reduction in the expression of genes, coding for the structural units of the light harvesting system (phycobilisomes), the photosynthesis systems (PSII and PSI), electron transfer systems (plastoquinones, cytochromes, and ferredoxin), reduced carbon fixation, and energy production systems (ATP synthase), was observed. Similar observations were reported in other studies (Huang et al., 2002, Gao et al. 2009), showing the impact of UV-B radiation on the cyanobacterium *Synechosystis*. This likely caused the reduction in photosynthetic activity and as such also the delayed photosynthetic growth after exposure to IR. The growth curves after exposure to 3200, 5000, and 6400 Gy, did show a significant delay of 10 till 17 days, likely due to radiation-induced damage in the cells, which required longer repair and recovery. Similar delay in growth after irradiation has been reported for the fast growing *D. radiodurans* irradiated with 15,000 Gy in which cell growth was restored only after 9 h (Liu et al. 2003). The photosynthesis measurements confirmed that the PSII system of *Arthrospira sp*. PCC 8005 was still partially functional even after irradiation with 6400 Gy, but doses of 3200 Gy or more clearly had a significant negative effect on photosynthetic quantum yield. Photosynthesis and electron transport chains are the main source of ROS under physiological conditions (Fig.4). Thus shutting down photosynthesis seems a logic response in an effort to reduce production of oxidants, which is already enhanced by IR. Photosynthesis and carbon fixation shutdown was also reported for *Arthrospira* in response to other stress factors such as nitrogen depletion stress (Deschoenmaeker et al. 2014), but was never before demonstrated in such detail, at RNA level. Only physiological examinations were reported and there are no transcriptomic data available for *Arthrospira* in response to UV stress (Wu et al. 2005). Hence, it was not possible to further explore such comparison.

It is generally assumed that the photosynthetic pigments are potent antioxidants, and thus high intracellular concentrations of such pigments would be beneficial in ROS and radiation defense. Therefore, it was surprising to find reduced level of these pigments in irradiated *Arthrospira* cells. Indeed, analysis of the pigment cells contents showed a significant decrease in allophycocyanin and phycocyanin concentration exposed to 3200, 5000, and 6400 Gy. Moreover, molecular data indicated specific downregulation of the enzymes involved in the biosynthesis of phycobilin and chlorophyll (Fig.4). The reduced pigment level is likely also the result of a controlled degradation guided by the enzyme NblA (Baier et al. 2001). The observed increased transcription level of *nblA* gene would indeed suggest active phycobilisome degradation (Aguirre von Wobeser et al. 2011). Such response has been explained as an effort of the cell to lower the proportion of light-harvesting phycobilisomes, to minimize the effective exposure to harsh environment. For example, the *nblA* gene is also induced in expression during the desiccation of the filamentous cyanobacterium *Microcoleus vaginatus* (Rajeev et al. 2013).

A wide variety of enzymatic and nonenzymatic antioxidant systems has been reported to be involved in cellular radiation resistance. The primary scavenging defense system to neutralize the ROS is mediated by antioxidants enzymes such as (1) superoxide dismutase (Sod) to neutralize superoxide radicals (formed in the presence of dissolved oxygen), and (2) catalase (Cat), GSH peroxidase (Gpx), peroxiredoxin and other peroxidases to neutralize hydroperoxides (produced by the radiolysis of water) (Mittler et al. 2004). Similar enzymatic antioxidant systems have been reported in cyanobacteria. It has been shown that filamentous cyanobacterium *N. punctiforme* ATCC 29133, induced mainly *sod* and *cat* to cope with oxidative stress generated by UVA exposure (Soule et al. 2013). The unicellular *Synechosystis sp*. PCC 6803 increased the transcription level of *sod* and *gpx* upon UVB exposure (Huang et al. 2002). Filamentous *Anabeana sp*. PCC7120 was able to cope with oxidative stress induced by salt and UVB by increasing the transcription levels of peroxiredoxin (Rai et al. 2013). Regarding *Anabeana* sp PCC 7120, the Ahp alkylhydroperoxide reductase protein was reported to play an important role in combating multitude stresses: heat, copper, salt, and IRs such as UVB (Mishra et al. 2009). While in *N. punctiforme* challenged by UVA radiations the *ahp* gene was downregulated (Soule et al. 2013). Also the highly gamma radiation resistant bacterium *D. radiodurans* relies on the enzymatic antioxidants, such as catalase, SOD, glutaredoxines, thioredoxin reductase, and alkylhydroperoxide reductase (White et al. 1999). *Arthrospira sp*. PCC 8005, however, is catalase-negative, which is an exceptional trait for all *Arthrospira* species (Fujisawa et al. 2010). *Arthrospira sp* PCC 8005 does contain a number of genes for peroxiredoxin and other putative peroxidases but for most of these genes, the expression was not changed or even reduced after irradiation. Either superoxide dismuatse (*sod*) in *Arthrospira* cells is not needed for radiation resistance, or either the unchanged level of expression could perhaps be due to a permanent high abundance, hence not needing additional induction. Either one of these hypothesizes would need to be investigated more in detail and confirmed. Regarding nonenzymatic antioxidants molecules recent studies revealed that protecting protein from oxidation may allow the cells to survive high number of double-strand breaks (DSBs) caused by IR. In several studies (Daly et al. 2010), it was shown that small antioxidant molecules such as the manganese and orthophosphate may play a key role in preventing protein oxidation, thereby protecting DNA-repair enzymes and increasing the efficiency in DNA repair. Our data indicate that *Arthrospira sp*. PCC 8005 seems to mainly rely on nonenzymatic thiol-based antioxidant systems such as glutathione (GSH) to cope with oxidative damage from IR comparing to other bacteria (Fig.4). Following exposure to gamma rays *Arthrospira sp*. PCC 8005 expressed several genes involved in the synthesis and recycling of GSH. GSH is a potent scavenger of singlet oxygen O_2_*, hydrogen peroxide H_2_O_2_, and the most harmful ROS hydroxyl radical OH^-^ (Larson 1988; Briviba et al. 1997). The thiol group is very reactive, and quickly neutralizes radicals. Interesting to know, is that *D. radiodurans* in fact lacks the classical GSH system, including gluthatione reductase (White et al. 1999). A recent study (Luan et al. 2014), did however, reported the presence of thiol-based antioxidant in *D. radiodurans*, called Bacithiol and considered as a substitute for GSH, with a role in its extreme resistance to gamma rays. In plants, GSH is one of the most crucial metabolites and is also considered as the most important intracellular defense system against ROS-induced oxidative damage (Gill and Tuteja 2010).

The transcription of the *fur* regulator was induced after gamma irradiation. Increased transcription of *fur* in response to redox stress has been shown for many bacteria and in general suggests a cellular reorganization to (1) increase the Fe^2+-^binding capacity, (2) repress iron uptake, and (3) promote iron storage system via bacterioferritine, DPS and ferritine (Castruita et al. 2006). It has been documented that in cyanobacteria, *fur* also contributes in the regulation of *isiA* which is an iron stress inducible gene coding for CP43′ proteins, presenting a pigment storage, a light harvesting ring structure surrounding PSI and energy dissipation system for PSII (Havaux et al. 2005; Ihalainen et al. 2005). The correlation between CP43′ and the core of PSII system, composed by CP34 and CP47, were reported (Singh and Sherman 2007). It has been shown that hydrogen peroxide could enhance *isiA* expression as well. The transcriptome analysis of *Synechocystis* PCC6803 challenged by iron deficiency or hydrogen peroxide revealed a high overlap in the induction of *isiA* gene after both treatments (Li et al. 2004).

Another strategy for cyanobacteria to cope with high doses of energetic photons (high light intensities, UV, IR) is to escape from stressing situation, by actively “moving away” from the stress source towards a less stressful (more shielded) environment (Castenholz and Garcia-Pichel 2002). Thus as defense against high light, the cyanobacteria evolved sensory photoreceptors in the cell envelope, to monitor photon flux and to activate different cell motility systems when needed (Song et al. 2011). A significant transcriptional increase of Cry-DASH photoreceptor gene was observed in *Arthrospira sp*. PCC8005 after irradiation. In *Synechosystis sp*. PCC 6803, Cry-DASH binds a pterine (Folate) derivate, which acts as light harvesting antenna sensor that absorbs photon energy generated by UVA/Bleu light and transfers the energy to flavin molecule, to activate a negative phototaxis away from the light stress (Mullineaux 2001; Moon et al. 2010).

Despite the activation of antioxidant systems, the data suggest a significant protein, lipids and DNA damage response. It is known that IR can cause oxidation or defolding of proteins that are needed for DNA repair (Shuryak and Brenner 2010). However, *Arthrospira* cells seem to have several molecular chaperones and folding catalysts in place to prevent or deal with this damage (Fig.4). The increased transcription of HSP, protease and peptidase coding genes by *Arthrospira*, was likely a response deal with the protein damage caused by the high doses of irradiation. HSP proteins such as the HSP70-type DnaK and GroEL/GroE are present in highly conserved forms in all bacteria, including cyanobacteria, and play crucial role in folding of newly synthesized proteins, preventing protein misfolding or aggregation and promoting protein degradation (Mary et al. 2004). Similar observation was reported for the cyanobacterium *Synechosystis sp*. PCC 6803 which exhibit an increase in chaperonin GroEL and GrpE after exposure to UVB (Huang et al. 2002). *Arthrospira* sp. PCC 8005 contains also five copies of HSP70-type *dnaK* gene, which is a lot comparing to *Deinococcus* which has only one copy, and the transcription level of three of them was significantly induced. Proteins that are oxidized beyond repair are dysfunctional and need to be removed and rapidly resynthesized (Karlin and Mrazek 2001). This requires efficient proteolytic degradation of the damaged proteins (Daly et al., 2010). Indeed, it has been shown for some bacteria, including *Deinococcus* that the level of intracellular proteolytic activity increased following radiation exposure (Servant et al. 2007; Daly et al., 2010). *Arthrospira sp*. PCC8005 induced the expression of a set of protease genes, including the *nblA* and *ftsH* genes which allow specific proteolysis of key components of the photosynthesis system (Tomoyasu et al. 1993). The FtsH protein has been shown to be involved in repair of the PSII system in *Synechocystis sp*. PCC 6803 (Li et al. 2004). Therefore it seems that the role of FtsH in PSII repair and D1 turnover might be conserved in both cyanobacteria and higher plants (Nixon et al. 2005).

For DNA, as the dose of electromagnetic IR increases, the linear density of bases damages and single strand breaks (SSBs) increases on both strands, which gives rise to DSBs. A specific dose of IR typically causes 40 times more SSBs than DSBs (Daly 2009). Our data showed, mainly, an activation of genes related to SSB, including the nucleotide excision repair (NER) and mismatch repair (MMR) systems in *Arthrospira* after irradiation (Fig.4). The NER multi-enzyme complex UvrABC, is an exonuclease that recognize the structural changes in DNA, and is involved in the removal of many types of DNA lesions (Petit and Sancar 1999). (MMR) system, involves proteins such as MutS1, MutL, and UvrD, and was also activated in *D. radiodurans* after exposure to gamma rays (Mennecier et al. 2006). Transcriptome data also revealed the induction of a NUDIX gene in *Arthrospira* exposed to gamma rays. Proteins of the NUDIX family are abundantly present in the highly radiation resistant bacterium *D. radiodurans* and are involved in the housecleaning and fast recovery of the cells (Bessman et al. 1996). The major role of these enzymes is the degradation and the export of damaged DNA to purify the cells (White et al. 1999). In addition to DNA repair genes, irradiated *Arthrospira sp*. PCC 8005 also overexpressed a large set of genes involved in the restriction modification mechanism, phage-immunity, and MGEs, possibly indicating radiation-induced genetic rearrangements. MGEs have been shown to be important components of genomic rearrangements (Yurchenko et al. 2011).

In most bacteria, the expression of the DNA repair genes is under control of the SOS response system, which is usually silent and only activated in the case of DNA damage (Zgur-Bertok 2013). The induction of the DNA repair system is regulated by two key SOS proteins, RecA and LexA. The coprotease RecA activates auto-cleavage of the transcriptional repressor LexA, which in turn then allows transcription of several genes involved in DNA damage repair, including RecA, which is also an essential DNA repair protein. This basic mechanism of LexA-dependent induction of DNA repair in response to radiation seems to be conserved in *E. coli* and *B. subtilis* (Lenhart et al. 2012). But LexA is not always required for the induction of RecA and DNA repair in general, as in *D. radiodurans,* for example, it was demonstrated that an abundance in RecA protein following gamma radiation was detected in a *lexA* knock-out mutant (Narumi et al. 2001). Also in cyanobacteria LexA does not seem to play a key role in radiation resistance. Whereas a homolog of LexA exists in most cyanobacteria (Jones 2014), it does not appear to be linked to DNA repair at least in *Synechocystis sp*. PCC 6803 (Li et al. 2010). Microarray analysis with a *lexA* mutant from *Synechocystis* revealed that LexA does not regulate typical DNA repair genes in this organism but, rather, might be important for genes involved in carbon metabolism (Domain et al. 2004). Our findings actually show that the *lexA* repressor gene is absent in the genome of *Arthrospira sp*. PCC8005. This has been also observed in other bacteria, such as *Helicobacter pylori* for example (Orillard et al. 2011). Hence, the DNA repair process seems to be independent of LexA in several organisms, including several radiation resistant organisms. Regarding the *recA* gene, our results showed no induction of this gene in *Arthrospira* after irradiation, neither at RNA level nor in proteins abundance. In *D. radiodurans recA* gene was induced after irradiation(Luan et al. 2014), but absence of *recA* induction after irradiation was also reported in *Synechocystis* (Domain et al. (2004) and *H. pylori* following UV and gamma radiation (Orillard et al. 2011). RecA is crucial for DNA repair and essential photosynthetic prokaryotes (Owttrim and Coleman 1989), thus probably also active in *Arthrospira* sp PCC8005 after irradiation. But it might be that *Arthrospira* cells constitutively produce large amounts of RecA protein at all times, even in the absence of DNA damage. For *H. pylori* this has indeed been shown and suggested as explanation for the absence of *recA* induction (Orillard et al. 2011). Thus, this could also be a hypothesis to test for.

The molecular analysis revealed also a new set of proteins that were induced seemingly in a dose-dependent manner following exposure to high doses of gamma rays. This set of genes was clustered in one genomic region, and annotated to code for “conserved hypothetical proteins.” Although it is well known that mRNA expression profiles are not always causative but can be merely correlative and are not always easily correlated with proteomic abundance (Gygi et al. 1999), these proteins were confirmed to be overexpressed both on RNA and protein level. Currently little can be said regarding the function of this interesting series of genes but it does appear that they exhibit a specific response to high acute doses of gamma irradiation (Fig.4). As far as we are aware, these proteins were never been reported as significantly expressed in *Arthrospira* in response to any other stress condition tested such as light stress (Matallana-Surget et al. 2014) or nitrogen deficiency) (Deschoenmaeker et al. 2014), which makes their response to IR rather unique and peculiar. It is possible that this set of genes may play an important role in the high radiation resistance of *Arthrospira sp*. PCC 8005. In general, a way to demonstrate the function of such proteins and their role in radiation resistance should involve the knockout of the genes in *Arthrospira* and to investigate such mutants in details. Unfortunately, unlike other unicellular cyanobacteria (e.g., *Synechococcus* and *Synechocystis*) and some filamentous cyanobacteria (e.g., *Anabaena*), genetic transformation of *Arthrospira* had limited success to date, which is a drawback. Nevertheless, these results opened new horizons of research that involve deeper investigation of cellular radiation sensitivity or resistance and the role of these proteins therein, which is currently on-going in our team.

## Conclusion

In summary, this study demonstrated for the first time the resistance of the free-floating filamentous and edible cyanobacteria *Arthrospira* to high doses of gamma rays. Thanks to the newly designed DNA microarray specific to *Arthrospira sp*. PCC 8005, molecular analysis of the response to irradiation stress could be done in depth. *Arthrospira* cells exposed to IR shut down photosynthesis and carbon fixation while protein and DNA damage gets repaired. Moreover there was no significant induction of classical bacterial enzymatic antioxidant system such as SOD and peroxide reductase, and RecA a key protein for DNA repair. In contrast, a clear activation of thiol-based antioxidant systems, such the GSH, was seen in *Arthrospira*, a system which is well known for plants but absent in many other radiation resistant bacteria such as *Deinococcus*. Beyond the response linked to genes with known functions, a novel set of seven conserved proteins of unknown function was identified. They were overexpressed in response to radiation exposure in a dose-dependent manner, providing new interesting targets for the future research. This first study was primarily observational in nature to screen for general cellular responses, but our basis for further detailed research.

## Supporting Information

**Figure S1.** RITA Facility of the Belgian reactor (BR2) at SCK•CEN. The cultures were irradiated in the dark inside the canister, submerged in the water, surrounded by four sources of ^60^Co providing gamma rays with energy of ca. 1.33, 1.17 Mev, with a dose rate 527 Gyh^−1^. The time required for irradiation was dependent of the total received dose indicated in the table added to the figure. For each irradiation dose, an equivalent culture was kept for the same time in dark as representative nonirradiated control. **Figure S2.** Transcriptomic expression profile for the 5854 coding DNA sequences (CDS, or “genes”) of *Arthrospira sp*. PCC 8005 after exposure to 3200 and 5000 Gy of gamma rays A: Represent all the differentially expressed genes (*P* < 0.05), (B) represent only the upregulated genes (*P* < 0.05), and FC > 2, and (C) represent only the downregulated genes (*P* < 0.05), and FC < 0.05 **Table S1.** Table presenting the specific growth rate for the cultures grown after irradiation for each time interval between two measurements. The last rows present respectively: the specific growth rate for each radiation dose established between two time points, was calculated with following formula . Data represent mean of three independent cultures (*n* = 3). An asterisk indicates a value for the irradiated sample which is significant (*P* < 0.05) different from the value of the nonirradiated control. Three asterisks indicate a value which is highly significant (*P* < 0.001). **Table S2.** Transcriptomic (microarray) results for genes known to be involved in photosensing, signaling, and motility. The fold change (FC) values listed are values for which p-value is *P* < 0.05, and are only considered biologically significant if FC > 2 or < 0.5. “NS” stands for not significant differentially expressed (*P* > 0.05). **Table S3.** Transcriptomic (microarray) results for genes known to be involved in secondary metabolite production. The fold change (FC) values listed are values for which p-value is *P* < 0.05, and are only considered biologically significant if FC > 2 or <0.5. “NS” stands for not significant differentially expressed (*P* > 0.05). **Table S4.** Transcriptomic (microarray) results for genes known to be involved in stress response and antioxidant defense. The fold change (FC) values listed are values for which p-value is *P* < 0.05, and are only considered biologically significant if FC > 2 or <0.5. “NS” stands for not significant differentially expressed (*P* > 0.05). “ND” stands for not detected. **Table S5.** Transcriptomic (microarray) results for genes known to be involved in protein repair and recycling. The fold change (FC) values listed are values for which *P* < 0.05, and are only considered biologically significant if FC > 2 or <0.5. “NS” stands for not significant differentially expressed. **Table S6.** Transcriptomic (microarray) results for genes known to be involved in DNA repair. The fold change (FC) values listed are only values for which *P* < 0.05, and are only considered biologically significant if FC > 2 or <0.5. “NS” stands for not significant differentially expressed, “ND” stands for not detected; NER for nucleotide excision repair, MMR for MisMatch Repair, and RM for restriction modification system. **Table S7.** Transcriptomic (microarray) results for genetic rearrangement “Transposases” The fold change (FC) values listed are values for which *P* < 0.05, and are only considered biologically significant if FC > 2 or <0.5. “NS” stands for not significant differentially expressed. **Table S8**. Transcriptomic (microarray) results for genetic rearrangement “Fax Elements” (in total there are seven clusters in the genome). The fold change (FC) values listed are values for which *P* < 0.05, and are only considered biologically significant if FC > 2 or <0.5. “NS” stands for not significant differentially expressed. **Table S9**. Transcriptomic (microarray) results for genetic rearrangement “CRISPRs” The fold change (FC) values listed are values for which *P* < 0.05, and are only considered biologically significant if FC > 2 or <0.5. “NS” stands for not significant differentially expressed. **Table S10**. Transcriptomic (microarray) results for genes coding for conserved hypothetical proteins “unknown function” The fold change (FC) values listed are values for which *P* < 0.05, and are only considered biologically significant if FC > 2 or <0.5. “NS” stands for not significant differentially expressed. **Table S11.** Proteomics results for 3200 Gy The fold change (FC) values listed are values for which *P* < 0.05, and are only considered biologically significant if FC > 1.25 or <0.8. “NS” stands for not significant differentially expressed. **Table S12.** Proteomics results for 5000 Gy The fold change (FC) values listed are values for which *P* < 0.05, and are only considered biologically significant if FC > 1.25 or <0.8. “NS” stands for not significant differentially expressed.

## References

[b1] Agarwal R, Rane SS, Sainis JK (2008). Effects of ^60^Co *γ* radiation on thylakoid membrane functions in *Anacystis nidulans*. J. Photochem. Photobiol., B.

[b2] Aguirre von Wobeser E, Ibelings BW, Bok J, Krasikov V, Huisman J, Matthijs HC (2011). Concerted changes in gene expression and cell physiology of the cyanobacterium *Synechocystis* sp. strain PCC 6803 during transitions between nitrogen and light-limited growth. Plant Physiol.

[b3] Baier K, Nicklisch S, Grundner C, Reinecke J, Lockau W (2001). Expression of two nblA-homologous genes is required for phycobilisome degradation in nitrogen-starved *Synechocystis* sp. PCC6803. FEMS Microbiol. Lett.

[b4] Baque M, Viaggiu E, Scalzi G, Billi D (2013). Endurance of the endolithic desert cyanobacterium Chroococcidiopsis under UVC radiation. Extremophiles.

[b5] Bauermeister A, Moeller R, Reitz G, Sommer S, Rettberg P (2011). Effect of relative humidity on *Deinococcus radiodurans*' resistance to prolonged desiccation, heat, ionizing, germicidal, and environmentally relevant UV radiation. Microb. Ecol.

[b6] Bebout BM, Garcia-Pichel F (1995). UV B-induced vertical migrations of Cyanobacteria in a microbial mat. Appl. Environ. Microbiol.

[b7] Belay A (2002). The potential application of Spirulina (*Arthrospira*) as a nutritional and therapeutic supplement in health management. J. Am. Nutraceutical Assoc.

[b8] Benjamini Y, Hochberg Y (1995). Controlling the false discovery rate – a practical and powerful approach to multiple testing. J Roy. Stat. Soc. B Methodol.

[b9] Bennett A, Bogorad L (1973). Complementary chromatic adaptation in a filamentous blue-green-alga. J. Cell Biol.

[b10] Bessman MJ, Frick DN, O'Handley SF (1996). The MutT proteins or “Nudix” hydrolases, a family of versatile, widely distributed, “housecleaning” enzymes. J. Biol. Chem.

[b11] Bhat VB, Madyastha KM (2001). Scavenging of peroxynitrite by phycocyanin and phycocyanobilin from *Spirulina platensis*: protection against oxidative damage to DNA (vol 285, pg 262, 2001). Biochem. Biophys. Res. Commun.

[b12] Billi D, Friedmann EI, Hofer KG, Caiola MG, Ocampo-Friedmann R (2000). Ionizing-radiation resistance in the desiccation-tolerant cyanobacterium Chroococcidiopsis. Appl. Environ. Microbiol.

[b13] Bolstad BM, Irizarry RA, Astrand M, Speed TP (2003). A comparison of normalization methods for high density oligonucleotide array data based on variance and bias. Bioinformatics.

[b14] Briviba K, Klotz LO, Sies H (1997). Toxic and signaling effects of photochemically or chemically generated singlet oxygen in biological systems. Biol. Chem.

[b15] Campbell D, Hurry V, Clarke AK, Gustafsson P, Oquist G (1998). Chlorophyll fluorescence analysis of cyanobacterial photosynthesis and acclimation. Microbiol. Mol. Biol. Rev.

[b16] Castenholz R, Whitton B, Potts M, Garcia-Pichel F (2002). Cyanobacterial responses to UV-radiation. The ecology of Cyanobacteria.

[b17] Castruita M, Saito M, Schottel PC, Elmegreen LA, Myneni S, Stiefel EI (2006). Overexpression and characterization of an iron storage and DNA-binding DPS protein from Trichodesmium erythraeum. Appl. Environ. Microbiol.

[b18] Cogne G, Lehmann B, Dussap C-G, Gros J-B (2003). Uptake of macrominerals and trace elements by the cyanobacterium *Spirulina platensis**Arthrospira platensis* PCC 8005) under photoautotrophic conditions: culture medium optimization. Biotechnol. Bioeng.

[b19] Daly MJ (2009). A new perspective on radiation resistance based on *Deinococcus radiodurans*. Nat. Rev. Microbiol.

[b20] Daly MJ, Gaidamakova EK, Matrosova VY, Kiang JG, Fukumoto R, Lee D-Y (2010). Small-molecule antioxidant proteome-shields in *Deinococcus radiodurans*. PLoS One.

[b21] Dartnell LR, Storrie-Lombardi MC, Mullineaux CW, Ruban AV, Wright G, Griffiths AD (2011). Degradation of cyanobacterial biosignatures by ionizing radiation. Astrobiology.

[b22] Deschoenmaeker F, Facchini R, Leroy B, Badri H, Zhang CC, Wattiez R (2014). Proteomic and cellular views of *Arthrospira* sp. PCC 8005 adaptation to nitrogen depletion. Microbiology.

[b23] Dillon JC, Phuc AP, Dubacq JP (1995). Nutritional value of the alga Spirulina. World Rev. Nutr. Diet.

[b24] Dobakova M, Tichy M, Komenda J (2007). Role of the PsbI protein in photosystem II assembly and repair in the cyanobacterium *Synechocystis* sp. PCC 6803. Plant Physiol.

[b25] Domain F, Houot L, Chauvat F, Cassier-Chauvat C (2004). Function and regulation of the cyanobacterial genes lexA, recA and ruvB: LexA is critical to the survival of cells facing inorganic carbon starvation. Mol. Microbiol.

[b26] Fernandes AP, Fladvad M, Berndt C, Andrésen C, Lillig CH, Neubauer P (2005). A novel monothiol glutaredoxin (Grx4) from *Escherichia coli* can serve as a substrate for thioredoxin reductase. J. Biol. Chem.

[b27] Frewen B, MacCoss MJ (2002). Using BiblioSpec for creating and searching tandem MS peptide libraries. Current protocols in bioinformatics.

[b28] Fujisawa T, Narikawa R, Okamoto S, Ehira S, Yoshimura H, Suzuki I (2010). Genomic structure of an economically important Cyanobacterium, *Arthrospira**Spirulina*) platensis NIES-39. DNA Res.

[b29] Gao Y, Xiong W, Li XB, Gao CF, Zhang YL, Li H (2009). Identification of the proteomic changes in *Synechocystis* sp. PCC 6803 following prolonged UV-B irradiation. J. Exp. Bot.

[b30] Gill SS, Tuteja N (2010). Reactive oxygen species and antioxidant machinery in abiotic stress tolerance in crop plants. Plant Physiol. Biochem.

[b31] Gladyshev E, Meselson M (2008). Extreme resistance of bdelloid rotifers to ionizing radiation. Proc. Natl. Acad. Sci. USA.

[b32] Gygi SP, Rochon Y, Franza BR, Aebersold R (1999). Correlation between protein and mRNA abundance in yeast. Mol. Cell. Biol.

[b33] Hasnain SRAS (2006). Gamma irradiation: impact on chromate resistant cyanobacteria. Science Asia.

[b34] Havaux M, Guedeney G, Hagemann M, Yeremenko N, Matthijs HC, Jeanjean R (2005). The chlorophyll-binding protein IsiA is inducible by high light and protects the cyanobacterium *Synechocystis* PCC6803 from photooxidative stress. FEBS Lett.

[b35] Hendrickx L, De Wever H, Hermans V, Mastroleo F, Morin N, Wilmotte A (2006). Microbial ecology of the closed artificial ecosystem MELiSSA (Micro-Ecological Life Support System Alternative): reinventing and compartmentalizing the Earth's food and oxygen regeneration system for long-haul space exploration missions. Res. Microbiol.

[b36] Hongsthong A, Sirijuntarut M, Yutthanasirikul R, Senachak J, Kurdrid P, Cheevadhanarak S (2009). Subcellular proteomic characterization of the high-temperature stress response of the cyanobacterium *Spirulina platensis*. Proteome Sci.

[b37] Huang L, McCluskey MP, Ni H, LaRossa RA (2002). Global gene expression profiles of the cyanobacterium *Synechocystis* sp. strain PCC 6803 in response to irradiation with UV-B and white light. J. Bacteriol.

[b38] Ihalainen JA, D'Haene S, Yeremenko N, van Roon H, Arteni AA, Boekema EJ (2005). Aggregates of the chlorophyll-binding protein IsiA (CP43′) dissipate energy in Cyanobacteria. Biochemistry.

[b39] Irizarry RA, Hobbs B, Collin F, Beazer-Barclay YD, Antonellis KJ, Scherf U (2003). Exploration, normalization, and summaries of high density oligonucleotide array probe level data. Biostatistics.

[b40] Janssen PJ, Morin N, Mergeay M, Leroy B, Wattiez R, Vallaeys T (2010). Genome sequence of the edible cyanobacterium *Arthrospira* sp. PCC 8005. J. Bacteriol.

[b41] Johnson TR, Haynes JI, Wealand JL, Yarbrough LR, Hirschberg R (1988). Structure and regulation of genes encoding phycocyanin and allophycocyanin from *Anabaena variabilis* ATCC 29413. J. Bacteriol.

[b42] Jones PR (2014). Genetic instability in cyanobacteria – an elephant in the room?. Front. Bioeng. Biotechnol.

[b43] Karlin S, Mrazek J (2001). Predicted highly expressed and putative alien genes of *Deinococcus radiodurans* and implications for resistance to ionizing radiation damage. Proc. Natl. Acad. Sci. USA.

[b44] Kraus MP (1969). Resistance of blue-green algae to ^60^Co gamma radiation. Radiat. Bot.

[b45] Krisko A, Radman M (2010). Protein damage and death by radiation in *Escherichia coli* and *Deinococcus radiodurans*. Proc. Natl. Acad. Sci. USA.

[b46] Larson RA (1988). The antioxidants of higher plants. Phytochemistry.

[b47] Latifi A, Ruiz M, Zhang CC (2009). Oxidative stress in cyanobacteria. FEMS Microbiol. Rev.

[b48] Le Caer S (2011). Water radiolysis: influence of oxide surfaces on H-2 production under ionizing radiation. Water-Sui.

[b49] Lenhart JS, Schroeder JW, Walsh BW, Simmons LA (2012). DNA repair and genome maintenance in *Bacillus subtilis*. Microbiol. Mol. Biol. Rev.

[b50] Li H, Singh AK, McIntyre LM, Sherman LA (2004). Differential gene expression in response to hydrogen peroxide and the putative PerR regulon of *Synechocystis* sp. strain PCC 6803. J. Bacteriol.

[b51] Li S, Xu M, Su Z (2010). Computational analysis of LexA regulons in Cyanobacteria. BMC Genom.

[b52] Liu Y, Zhou J, Omelchenko MV, Beliaev AS, Venkateswaran A, Stair J (2003). Transcriptome dynamics of *Deinococcus radiodurans* recovering from ionizing radiation. Proc. Natl. Acad. Sci. USA.

[b53] Luan H, Meng N, Fu J, Chen X, Xu X, Feng Q (2014). Genome-wide transcriptome and antioxidant analyses on gamma-irradiated phases of *Deinococcus radiodurans* R1. PLoS ONE.

[b54] MacLean B, Tomazela DM, Shulman N, Chambers M, Finney GL, Frewen B (2010). Skyline: an open source document editor for creating and analyzing targeted proteomics experiments. Bioinformatics.

[b55] Mary I, Tu CJ, Grossman A, Vaulot D (2004). Effects of high light on transcripts of stress-associated genes for the cyanobacteria Synechocystis sp. PCC 6803 and Prochlorococcus MED4 and MIT9313. Microbiology.

[b56] Masojídek J, Vonshak A, Suggett DJ, Prášil O, Borowitzka MA, Torzillo G (2010). Chlorophyll fluorescence applications in microalgal mass cultures. Chlorophyll a fluorescence in aquatic sciences: methods and applications.

[b57] Matallana-Surget S, Derock J, Leroy B, Badri H, Deschoenmaeker F, Wattiez R (2014). Proteome-wide analysis and diel proteomic profiling of the Cyanobacterium *Arthrospira* platensis PCC 8005. PLoS ONE.

[b58] Mennecier S, Servant P, Coste G, Bailone A, Sommer S (2006). Mutagenesis via IS transposition in *Deinococcus radiodurans*. Mol. Microbiol.

[b59] Mishra Y, Chaurasia N, Rai LC (2009). AhpC (alkyl hydroperoxide reductase) from *Anabaena* sp. PCC 7120 protects *Escherichia coli* from multiple abiotic stresses. Biochem. Biophys. Res. Commun.

[b60] Mittler R, Vanderauwera S, Gollery M, Van Breusegem F (2004). Reactive oxygen gene network of plants. Trends Plant Sci.

[b61] Moon Y-J, Kim S-J, Park YM, Chung Y-H (2010). Sensing UV/blue: Pterin as a UV-A absorbing chromophore of cryptochrome. Plant Signal. Behav.

[b62] Mullineaux CW (2001). How do cyanobacteria sense and respond to light?. Mol. Microbiol.

[b63] Mullineaux CW, Tobin MJ, Jones GR (1997). Mobility of photosynthetic complexes in thylakoid membranes. Nature.

[b64] Narumi I, Satoh K, Kikuchi M, Funayama T, Yanagisawa T, Kobayashi Y (2001). The LexA protein from *Deinococcus radiodurans* is not involved in RecA induction following gamma irradiation. J. Bacteriol.

[b65] Nixon PJ, Barker M, Boehm M, de Vries R, Komenda J (2005). FtsH-mediated repair of the photosystem II complex in response to light stress. J. Exp. Bot.

[b66] Orillard E, Radicella JP, Marsin S (2011). Biochemical and cellular characterization of *Helicobacter pylori* RecA, a protein with high-level constitutive expression. J. Bacteriol.

[b67] Owttrim GW, Coleman JR (1989). Regulation of expression and nucleotide sequence of the *Anabaena variabilis* recA gene. J. Bacteriol.

[b68] Petit C, Sancar A (1999). Nucleotide excision repair: from *E. coli* to man. Biochimie.

[b69] Pinto F, Thapper A, Sontheim W, Lindblad P (2009). Analysis of current and alternative phenol based RNA extraction methodologies for cyanobacteria. BMC Mol. Biol.

[b70] Rai S, Singh S, Shrivastava A, Rai LC (2013). Salt and UV-B induced changes in *Anabaena* PCC 7120: physiological, proteomic and bioinformatic perspectives. Photosynth. Res.

[b71] Rajeev L, da Rocha UN, Klitgord N, Luning EG, Fortney J, Axen SD (2013). Dynamic cyanobacterial response to hydration and dehydration in a desert biological soil crust. ISME J.

[b72] Ramadan MF, Asker MMS, Ibrahim ZK (2008). Functional bioactive compounds and biological activities of *Spirulina platensis* lipids. Czech. J. Food Sci.

[b73] Robinson CK, Webb K, Kaur A, Jaruga P, Dizdaroglu M, Baliga NS (2011). A major role for nonenzymatic antioxidant processes in the radioresistance of *Halobacterium salinarum*. J. Bacteriol.

[b74] Schilling B, Rardin MJ, MacLean BX, Zawadzka AM, Frewen BE, Cusack MP (2012). Platform-independent and Label-free Quantitation of Proteomic Data Using MS1 Extracted Ion Chromatograms in Skyline APPLICATION TO PROTEIN ACETYLATION AND PHOSPHORYLATION. Mol. Cell Proteomics.

[b75] Servant P, Jolivet E, Bentchikou E, Mennecier S, Bailone A, Sommer S (2007). The ClpPX protease is required for radioresistance and regulates cell division after gamma-irradiation in *Deinococcus radiodurans*. Mol. Microbiol.

[b76] Shuryak I, Brenner D (2010). Effects of radiation quality on interactions between oxidative stress, protein and DNA damage in *Deinococcus radiodurans*. Radiat. Environ. Biophys.

[b77] Singh A, Sherman L (2007). Reflections on the function of IsiA, a cyanobacterial stress-inducible, Chl-binding protein. Photosynth. Res.

[b78] Singh H, Fernandes T, Apte S (2010a). Unusual radioresistance of nitrogen-fixing cultures of Anabaena strains. J. Biosci.

[b79] Singh SP, Hader DP, Sinha RP (2010b). Cyanobacteria and ultraviolet radiation (UVR) stress: mitigation strategies. Ageing Res. Rev.

[b80] Singh H, Anurag K, Apte S (2013). High radiation and desiccation tolerance of nitrogen-fixing cultures of the cyanobacterium *Anabaena* sp. strain PCC 7120 emanates from genome/proteome repair capabilities. Photosynth. Res.

[b81] Smyth GK (2004). Linear models and empirical bayes methods for assessing differential expression in microarray experiments. Stat. Appl. Genet. Mol. Biol.

[b82] Song J-Y, Cho HS, Cho J-I, Jeon J-S, Lagarias JC, Park Y-I (2011). Near-UV cyanobacteriochrome signaling system elicits negative phototaxis in the cyanobacterium Synechocystis sp. PCC 6803. Proc. Natl. Acad. Sci. USA.

[b83] Soule T, Gao Q, Stout V, Garcia-Pichel F (2013). The global response of *Nostoc punctiforme* ATCC 29133 to UVA stress, assessed in a temporal DNA microarray study. Photochem. Photobiol.

[b84] Tomoyasu T, Yuki T, Morimura S, Mori H, Yamanaka K, Niki H (1993). The *Escherichia coli* FtsH protein is a prokaryotic member of a protein family of putative ATPases involved in membrane functions, cell cycle control, and gene expression. J. Bacteriol.

[b85] Vass IZ, Kos PB, Sass L, Nagy CI, Vass I (2013). The ability of cyanobacterial cells to restore UV-B radiation induced damage to Photosystem II is influenced by photolyase dependent DNA repair. Photochem. Photobiol.

[b86] Vonshak A (1990). Recent advances in microalgal biotechnology. Biotechnol. Adv.

[b87] Vonshak A, Guy R, Guy M (1988). The response of the filamentous cyanobacterium *Spirulina platensis* to salt stress. Arch. Microbiol.

[b88] White O, Eisen JA, Heidelberg JF, Hickey EK, Peterson JD, Dodson RJ (1999). Genome sequence of the radioresistant bacterium *Deinococcus radiodurans* R1. Science.

[b89] Wilson GG, Murray NE (1991). Restriction and modification systems. Annu. Rev. Genet.

[b90] Woo HJ, Kang JY, Choi YK, Park YS (2002). Production of sepiapterin in *Escherichia coli* by coexpression of cyanobacterial GTP cyclohydrolase I and human 6-pyruvoyltetrahydropterin synthase. Appl. Environ. Microbiol.

[b91] Wu H, Gao K, Villafane VE, Watanabe T, Helbling EW (2005). Effects of solar UV radiation on morphology and photosynthesis of filamentous cyanobacterium *Arthrospira* platensis. Appl. Environ. Microbiol.

[b92] Yurchenko NN, Kovalenko LV, Zakharov IK (2011). Transposable elements: instability of genes and genomes. Russ. J. Genet. Appl. Res.

[b93] Zgur-Bertok D (2013). DNA damage repair and bacterial pathogens. PLoS Pathog.

