# Supplementary material for: Molecular investigation of the radiation resistance of edible cyanobacterium Arthrospira sp. PCC 8005
Source: Microbiologyopen. 2015 Feb 12;4(2):187–207. doi: 10.1002/mbo3.229 (PMC4398503; doi:10.1002/mbo3.229)
Supplement: Supplementary file 1 — Figure S1. RITA Facility of the Belgian reactor (BR2) at SCK•CEN. The cultures were irradiated in the dark inside the canister, submerged in the water, surrounded by four sources of 60Co providing gamma rays with energy of ca. 1.33, 1.17 Mev, with a dose rate 527 Gyh−1. The time required for irradiation was dependent of the total received dose indicated in the table added to the figure. For each irradiation dose, an equivalent culture was kept for the same time in dark as representative nonirradiated control. Figure S2. Transcriptomic expression profile for the 5854 coding DNA sequences (CDS, or “genes”) of Arthrospira sp. PCC 8005 after exposure to 3200 and 5000 Gy of gamma rays A: Represent all the differentially expressed genes (P < 0.05), (B) represent only the upregulated genes (P < 0.05), and FC > 2, and (C) represent only the downregulated genes (P < 0.05), and FC < 0.05 Table S1. Table presenting the specific growth rate for the cultures grown after irradiation for each time interval between two measurements. The last rows present respectively: the specific growth rate for each radiation dose established between two time points, was calculated with following formula . Data represent mean of three independent cultures (n = 3). An asterisk indicates a value for the irradiated sample which is significant (P < 0.05) different from the value of the nonirradiated control. Three asterisks indicate a value which is highly significant (P < 0.001). Table S2. Transcriptomic (microarray) results for genes known to be involved in photosensing, signaling, and motility. The fold change (FC) values listed are values for which p-value is P < 0.05, and are only considered biologically significant if FC > 2 or < 0.5. “NS” stands for not significant differentially expressed (P > 0.05). Table S3. Transcriptomic (microarray) results for genes known to be involved in secondary metabolite production. The fold change (FC) values listed are values for which p-value is P < 0.05, and a [file mbo30004-0187-sd1.docx]

Supplemental Figures

| Doses | Exposure time |
| --- | --- |
| IR 200 Gy | **21min47sec** |
| IR 800 Gy | **1h27min7sec** |
| IR 1600 Gy | **2h54min15sec** |
| IR 3200 Gy | **5h48min30sec** |
| IR 5000 Gy | **9h4min30sec** |
| IR 6400 Gy | **11h36min** |


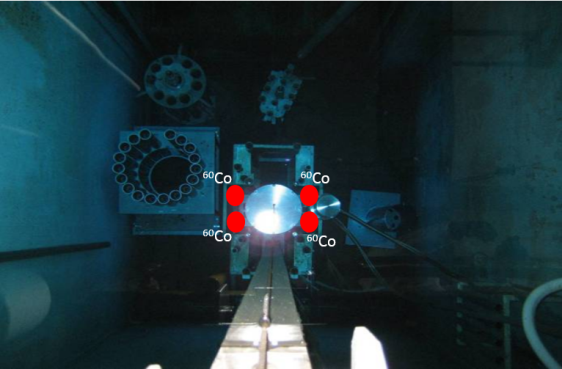


**Figure S1:** RITA Facilty of the Belgian reactor (BR2) at SCK•CEN. The cultures were irradiated in the dark inside the cansiter, submerged in the water, surrounded by 4 sources of ^60^Co providing gamma rays with energy of ca. 1.33 Mev and 1.17 Mev, with a dose rate 527 Gy h^-1^ . The time required for irradiation was dependent of the total received dose, as indicated in the table added to the figure. For each irradiation dose, an equivalent culture was kept for the same time in dark as representative non-irradiated control.

**Table S1**: Table presenting the specific growth rate for the cultures grown after irradiation for each time interval between 2 measurements. The specific growth rate for each radiation dose established between two time points, was calculated with following formula $\mu=\frac{\ln\left( OD750 at t2 \right)-\ln\left( OD750 at t1 \right)}{t2-t1}$. The last row presents the maximum growht rate obtained for each radiation dose. Data represent mean of three independent cultures (n= 3). An asterisk indicates a value for the irradiated sample which is significant (p<0.05) different from the value of the non-irradiated control. Three asterisk indicate a value which is highly significant (p<0.001).

| Time intervals (Days) | CTR  (n=3) | 200 Gy  (n=3) | 800 Gy  (n=3) | 1600 Gy  (n=3) | 3200 Gy  (n=3) | 5000 Gy  (n=3) | 6400 Gy  (n=3) |
| --- | --- | --- | --- | --- | --- | --- | --- |
| 1 -8 | **0,326** | 0,277 | 0,208 | 0,101 | 0 | 0 | 0 |
| 8-10 | 0,212 | **0,303** | **0,398** | **0,388** | 0 | 0 | 0 |
| 10-15 | 0,122 | 0,123 | 0,137 | 0,311 | **0,761** | **0,542** | 0 |
| 15-17 | -0,002 | 0,085 | 0,204 | 0,148 | 0,282 | 0,003 | 0 |
| 17-21 | 0,002 | 0,004 | -0,03 | -0,012 | 0,045 | 0,232 | 0,505 |
| 21-24 | -0,055 | -0,073 | -0,069 | -0,055 | -0,081 | 0,167 | 0,547 |
| 24-25 | -- | -- | -- | -- | -- | -- | **0,577** |
| Lag time  (µ = 0) (days) | 0 | 0 | 0 | 0 | 10* | 10 | 17 |
| Maximum growth rate µmax | **0,326**  **±0,135** | **0,303**  **±0,02** | **0,398**  ±0,096 | **0,388**  ±0,130 | **0,761***  ±0,012 | **0,542**  ±0,092 | **0,577**  ±0,272 |

**B : Up-regulated**

**3200 Gy**

**5000 Gy**


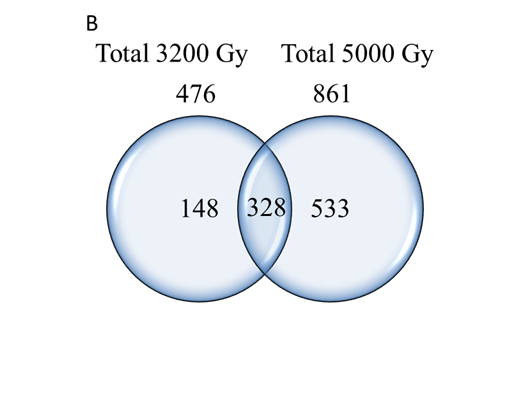


**A : Total genes**

**3200 Gy**

**5000 Gy**


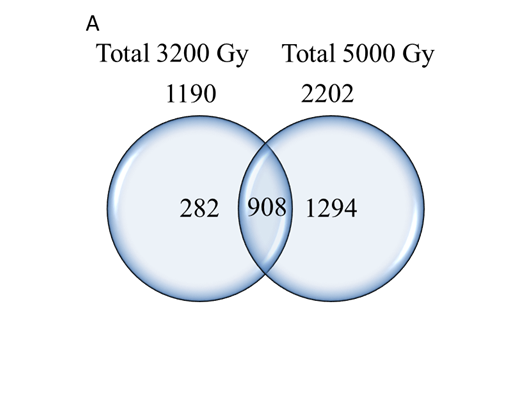


**3200 Gy**

**5000 Gy**

**C : Down-regulated**


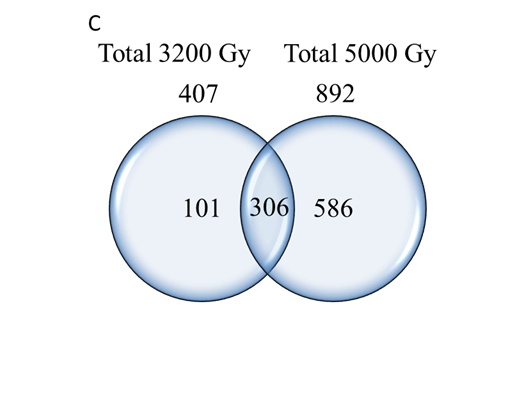


Figure S2: Transcriptomic expression profile for the 5854 coding DNA sequences (CDS, or “genes”) of *Arthrospira sp*. PCC 8005 after exposure to 3200 Gy and 5000 Gy of gamma rays. A: Representation of all the differentially expressed genes (p<0.05), B: Representation of only the upregulated genes (p<0.05), and FC>2, and C: Representation of only the down regulated genes (p<0.05), and FC<0.05.

Supplemental Tables

Table S2. Transcriptomic (microarray) results for genes known to be involved in photosensing, signalling and motility. The fold change (FC) values listed are values for which p-value is p<0.05, and are only considered biologically significant if FC > 2 or < 0.5. 'NS' stands for not significant differentially expressed (p>0.05).

| PHOTOSENSING & MOTILITY | Accession number | **Gene** | **Protein Function** | Fold change 3200 Gy | Fold change 5000 Gy |
| --- | --- | --- | --- | --- | --- |
| Chromophores | ARTHROv5_40253 | *ygcM* | 6-pyruvoyl tetrahydrobiopterin synthase (PTPS) | 1,39 | 2,10 |
|  | ARTHROv5_40925 | *folE* | [GTP cyclohydrolase I](http://en.wikipedia.org/wiki/GTP_cyclohydrolase_I) (GTPCH) | 7,31 | 12,54 |
|  | ARTHROv5_20034 | *fold* | Bifunctional protein | NS | 0,26 |
|  | ARTHROv5_40926 |  | Putative metallo-dependent phosphatase | 7,60 | 11,59 |
|  | ARTHROv5_10963 | *cry* | Cryptochrome-DASH protein | 2,22 | 3,36 |
|  | ARTHROv5_20097 |  | Putative diguanylate cyclase/phosphodiesterase (GGDEF & EAL domains) with Phytochrome (GAF) | 12,83 | 7,96 |
|  | ARTHROv5_20098 |  | Putative diguanylate cyclase (GGDEF domain) | NS | NS |
|  | ARTHROv5_20099 |  | Sensor protein | 2,58 | 2,70 |
|  | ARTHROv5_30350 |  | Signal transduction histidine kinase:Sensor with GAF domain | 1,91 | 2,35 |
|  | ARTHROv5_10439 |  | Conserved protein of unknown function | 3,43 | 7,79 |
|  | ARTHROv5_10440 |  | Response regulator receiver modulated PAS/PAC sensor(S) | 1,32 | 3,60 |
| Chemotaxis | ARTHROv5_11061 | *cheY1* | Putative response regulator receiver | 1,16 | 2,18 |
|  | ARTHROv5_11062 |  | Putative ABC transporter, ATP-binding protein | NS | 2,51 |
|  | ARTHROv5_11063 |  | Hypothetical protein | NS | NS |
|  | ARTHROv5_11064 | *cheY2* | Response regulator receiver | 1,72 | 3,01 |
|  | ARTHROv5_60796 | *cheY3* | Two-component response regulator | 1,35 | 9,01 |
|  | ARTHROv5_60571 | *cheC1* | CheC inhibitor of MCP methylation | 1,75 | 2,45 |
|  | ARTHROv5_60572 | *cheB1* | Fused chemotaxis regulator | 3,13 | 3,44 |
|  | ARTHROv5_60573 | *cheR1* | CheR Chemotaxis protein methyltransferase, MCP methyltransferase, Protein-glutamate O-methyltransferase | NS | NS |
|  | ARTHROv5_60574 |  | Putative PAS/PAC sensor protein | NS | NS |
|  | ARTHROv5_60575 |  | Methyl-accepting chemotaxis protein | NS | NS |
|  | ARTHROv5_60576 | *cheW1* | CheW protein, purine-binding chemotaxis protein, Chemotaxis signal transduction protein | NS | 1,9 |
|  | ARTHROv5_60577 | *cheA1* | CheA signal transduction histidine kinase | 1,64 | 2,05 |
|  | ARTHROv5_11949 | *cheB2* | Chemotaxis Response Regulator protein | 1,94 | 2,37 |
| Motility | ARTHROv5_60996 |  | Putative Pilin biogenesis protein | 0,86 | 0,32 |
|  | ARTHROv5_50098 |  | Putative leader peptidase (Prepilin peptidase) | 0,72 | 0,38 |
| Gas vacuoles | ARTHROv5_12032 | *gvpA* | Gas vesicle structural protein A | 0,40 | 0,25 |
|  | ARTHROv5_12033 | *gvpC1* | Gas vesicle structural protein | 0,18 | 0,16 |
|  | ARTHROv5_12034 |  | Conserved protein of unknown function | 0,24 | 0,22 |
|  | ARTHROv5_12037 | *gvpC2* | Gas vesicle structural protein | 0,23 | 0,18 |
|  | ARTHROv5_12038 |  | Hypothetical protein | 0,09 | 0,09 |
|  | ARTHROv5_12039 | *gvpN* | Gas vesicle protein | 0,06 | 0,08 |
|  | ARTHROv5_12040 | *gvpJ* | Gas vesicle synthesis protein | 0,23 | 0,32 |
|  | ARTHROv5_11240 | *gvpW* | Putative gas vesicle protein | 1,03 | 0,60 |

Table S3. Transcriptomic (microarray) results for genes known to be involved in secondary metabolite production. The fold change (FC) values listed are values for which p-value is p<0.05, and are only considered biologically significant if FC > 2 or < 0.5. 'NS' stands for not significant differentially expressed (p>0.05).

| METABOLITE biosynthesis | Accession number | Gene | | | | | Protein Function | Fold change 3200 Gy | Fold change 5000 Gy |  |  |
| --- | --- | --- | --- | --- | --- | --- | --- | --- | --- | --- | --- |
| Glycogen | ARTHROv5_41216 | *glgA1* | | | | | Glycogen synthase 1 | 0,30 | 0,22 |  |  |
|  | ARTHROv5_60979 | *glgA2* | | Glycogen synthase 2 | | | | 0,59 | 0,59 |  |  |
|  | ARTHROv5_20114 | *glgP* | | | Glycogen/starch/alpha-glucan phosphorylases | | | 0,36 | 0,23 |  |  |
|  | ARTHROv5_61087 | *glgX2* | | | Glycogen debranching enzyme | | | 0,85 | 0,24 |  |  |
|  | ARTHROv5_60834 | *glgB* | | | 1,4-alpha-glucan branching enzyme | | | NS | 0,56 |  |  |
| Lipid | ARTHROv5_10240 | *fabF1* | | | 3-oxoacyl-[acyl-carrier-protein] synthase 2 | | | 0,69 | 0,32 |  |  |
|  | ARTHROv5_30260 | *fabG1* | | | 3-oxoacyl-[acyl-carrier-protein] reductase | | | 0,58 | 0,24 |  |  |
|  | ARTHROv5_30826 | *fabI* | | | Enoyl-[acyl-carrier-protein] reductase [NADH] | | | 0,50 | 0,32 |  |  |
|  | ARTHROv5_41232 | *fabZ* | | | (3R)-hydroxymyristoyl-[acyl-carrier-protein] dehydratase | | | 0,28 | 0,19 |  |  |
|  | ARTHROv5_40656 | *desD* | | | Delta-6 fatty acid desaturase | | | 0,39 | 0,19 |  |  |
|  | ARTHROv5_60707 | *desA* | | | Delta-12 fatty acid desaturase | | | 0,59 | 0,31 |  |  |
| Glucosylglycerol (GG) | ARTHROv5_10080 | *stpA* | | | Glucosylglycerol 3-phosphatase | | | 0,73 | 0,23 |  |  |
|  | ARTHROv5_10816 | *suhB* | | | Inositol monophosphatase | | | 0,51 | 0,49 |  |  |
|  | ARTHROv5_30402 | *gpsA* | | | NAD+ dependent glycerol-3-phosphate dehydrogenase | | | 0,55 | 0,41 |  |  |
|  | ARTHROv5_30403 | *glpD* | | | sn-glycerol-3-phosphate dehydrogenase, aerobic, FAD/NAD(P)-binding | | | 0,43 | 0,36 |  |  |
|  | ARTHROv5_30404 | *ggpS* | | | Glucosylglycerol-phosphate synthase | | | 0,22 | 0,36 |  |  |
| Pattelamide A | ARTHROv5_40574 | *patB* | | | conserved hypothetical protein | | | 0,43 | 0,06 |  |  |
|  | ARTHROv5_40575 | *patC* | | | conserved hypothetical protein | | | 0,42 | 0,05 |  |  |
|  | ARTHROv5_40587 | *patF* | | | conserved hypothetical protein | | | 0,53 | 0,36 |  |  |
| Toxin/AntiToxin | ARTHROv5_12008 | | *mazF* | | | mRNA interferase | | 2,57 | 7,52 | |  |
|  | ARTHROv5_12009 | |  | | | Toxin/ antitoxin | | 1,26 | 5,72 | |  |
|  | ARTHROv5_40682 | |  | | | putative antitoxin of toxin-antitoxin system, family Axe | | NS | NS | |  |
|  | ARTHROv5_40683 | |  | | | putative toxin of toxin-antitoxin system, family Txe/YoeB | | 0,23 | 0,37 | |  |
|  | ARTHROv5_11210 | |  | | | putative antitoxin of toxin-antitoxin system, YefM-like | | NS | 8,02 | |  |
|  | ARTHROv5_11211 | |  | | | putative toxin of toxin-antitoxin system, YoeB-like | | NS | 6,03 | |  |
| Polyhydroxybutyrate (PHB ) | ARTHROv5_10499 | *phaE* | | | Poly(R)-hydroxyalkanoic acid synthase, class III, subunit | | | NS | NS |  |  |
|  | ARTHROv5_10500 | *phaC* | | | Poly(R)-hydroxyalkanoic acid synthase, class III, subunit | | | NS | 2,24 |  |  |
|  | ARTHROv5_60059 | *phbA* | | | acetyl-CoA acetyltransferase with thiolase domain (Acetoacetyl-CoA thiolase) | | | NS | 0,20 |  |  |
|  | ARTHROv5_10067 |  | | | 3-hydroxyisobutyrate dehydrogenase | | | 2,81 | 3,29 |  |  |
| Patatin | ARTHROv5_30480 |  | | | Patatin | | | NS | 0,50 |  |  |
|  | ARTHROv5_10493 |  | | | Putative patatin-like phospholipase | | | NS | NS |  |  |
|  | ARTHROv5_10494 |  | | | Hypothetical protein | | | NS | 2,03 |  |  |
|  | ARTHROv5_10495 |  | | | Putative patatin-like phospholipase | | | NS | 2,84 |  |  |
| Haemolysin | ARTHROv5_40115 |  | | | Haemolysin-type calcium-binding toxin (secreted) | | | 2,33 | 2,12 |  |  |
|  | ARTHROv5_40224 |  | | | Putative haemolysin-type calcium-binding toxin, RTX-like | | | NS | 0,38 |  |  |
|  | ARTHROv5_12129 | |  | | | Putative haemolysin A-like cytotoxin | | 2,04 | 2,08 | | |

Table S4. Transcriptomic (microarray) results for genes known to be involved in stress response and antioxidant defence. The fold change (FC) values listed are values for which p-value is p<0.05, and are only considered biologically significant if FC > 2 or < 0.5. 'NS' stands for not significant differentially expressed (p>0.05). 'ND' stands for not detected.

| ANTIOXIDANTS | Accession number | **Gene** | **Protein Function** | Fold change 3200 Gy | Fold change 5000 Gy |
| --- | --- | --- | --- | --- | --- |
| **Catalase (CAT)** | Gene absent | *kat* | Catalase | ND | ND |
| **Superoxide dismutase (SOD)** | ARTHROv5_50113 | *sodB* | Superoxide dismutase, Fe | 0,84 | 0,68 |
| Peroxide reductase | ARTHROv5_30341 |  | Putative peroxiredoxin | 2,20 | 1,66 |
|  | ARTHROv5_20231 | *ahp* | Putative alkyl hydroperoxide reductase, AhpC-like | 0,76 | 0,98 |
| **Glutathione (GSH)** | ARTHROv5_30647 | *gshB* | Glutathione synthase | 2,13 | 3,80 |
|  | ARTHROv5_60129 |  | Putative Lactoylglutathione lyase (Glyoxalase I) | 3,50 | 5,60 |
|  | ARTHROv5_60820 |  | Glutathionylspermidine synthase | 1,87 | 2,80 |
|  | ARTHROv5_11282 |  | Hydroxyacylglutathione hydrolase | 0,32 | 0,68 |
|  | ARTHROv3_430028 | *gor* | Glutathione oxidoreductase | 0,74 | 0,57 |
|  | ARTHROv5_60735 | *grx* | Monothiol glutaredoxin | NS | NS |
| Thioredoxin | ARTHROv5_30047 | *trxA1* | Thioredoxin-1 | 0,49 | 0,39 |
|  | ARTHROv5_30261 | *trxA2* | Thioredoxin-1 | 0,86 | 1,39 |
|  | ARTHROv5_11001 | *trxA4* | Thioredoxin-1 | 0,39 | 0,16 |
|  | ARTHROv5_41074 | *trxB* | Thioredoxin-disulfide reductase | 2,30 | 1,98 |
|  | ARTHROv5_10124 | *dbsA1* | Putative disulfide oxidoreductase (fragment) | 2,34 | 2,55 |
|  | ARTHROv5_10131 | *dbsA2* | Putative disulfide oxidoreductase | NS | 2,46 |
| **Fe-homeostasis** | ARTHROv5_30342 | *fur* | Transcriptional regulator, Fur family protein | 2,68 | 1,94 |
|  | ARTHROv5_11765 | *sufR* | Iron-sulphur cluster biosynthesis transcriptional regulator | NS | 2,12 |
|  | ARTHROv5_61180 | *isiA* | Iron stress-induced chlorophyll-binding protein (CP43') | 4,98 | 1,81 |
|  | ARTHROv5_40087 | *bcp1* | Bacterioferritin comigratory protein | 0,59 | 0,21 |
|  | ARTHROv5_10833 | *bcp4* | Bacterioferritin comigratory protein | 0,70 | 0,45 |
|  | ARTHROv5_60045 |  | Ferrous Iron(II) transporter | 0,43 | NS |
|  | ARTHROv5_60046 |  | Ferrous Iron(II) transporter, B domain protein | NS | NS |
|  | ARTHROv5_60047 | *feoA* | Putative FeoA family protein | NS | NS |
| **Stress response** | ARTHROv5_10153 | *dps* | DNA protection during starvation protein | NS | NS |
|  | ARTHROv5_11130 | *uspA* | Universal stress protein | 1,55 | 4,72 |

Table S5. Transcriptomic (microarray) results for genes known to be involved in proteinprotection. The fold change (FC) values listed are values for which p-value is p<0.05, and are only considered biologically significant if FC > 2 or < 0.5. 'NS' stands for not significant differentially expressed (p>0.05).

| PROTEIN DAMAGE CLEAN-UP | Accession number | **Gene** | **Protein Function** | Fold change 3200 Gy | Fold change 5000 Gy |
| --- | --- | --- | --- | --- | --- |
| HSP70-type | ARTHROv5_10362 | *dnaK1* | Chaperone protein, HSP70-type | 1,99 | 3,97 |
|  | ARTHROv5_11814 | *dnaK2* | Chaperone protein, HSP70-type | NS | NS |
|  | ARTHROv5_11998 | *dnaK3* | Chaperone protein, HSP70-type (fragment, part 2) | NS | NS |
|  | ARTHROv5_30014 | *dnaK4* | Chaperone protein, HSP70-type | NS | 4,66 |
|  | ARTHROv5_30685 | *dnaJ* | Chaperone protein,, HSP70-type | NS | 2,25 |
|  | ARTHROv5_30686 | *dnaK5* | Chaperone protein, HSP70-type | 2,20 | 3,49 |
|  | ARTHROv5_61127 | *cbpA* | Curved DNA-binding protein, DnaJ homologue | 0,51 | 5,35 |
| HSP60-type | ARTHROv5_30259 | *groL1* | Cpn60 chaperonin, large subunit of GroESL | 2,50 | 2,97 |
|  | ARTHROv5_61181 | *groL2* | Cpn60 chaperonin, large subunit of GroESL | 2,34 | 2,35 |
|  | ARTHROv5_61182 | *groS* | Cpn10 chaperonin GroES | 1,49 | 1,48 |
| HSP100-type | ARTHROv5_11700 | *clpS2* | ATP-dependent Clp protease adapter protein | 1,32 | 2,40 |
|  | ARTHROv5_60878 | *ftsH* | ATP-dependent zinc-metallo protease | 1,27 | 2,77 |
|  | ARTHROv5_61095 | *nblA2* | Phycobilisome degradation protein | NS | 2,17 |

Table S6. Transcriptomic (microarray) results for genes known to be involved in DNA damage repair. The fold change (FC) values listed are only values for which p-value is p<0.05, and are only considered biologically significant if FC > 2 or < 0.5. 'NS' stands for not significant differentially expressed (p>0.05), 'ND' stands for not detected, NER for nucleotide excision repair, MMR for MisMatch Repair, and RM for restriction modification system.

| DNA repair | Accession number | **Gene** | **Protein Function** | Fold change 3200 Gy | Fold change 5000 Gy |
| --- | --- | --- | --- | --- | --- |
|  | ARTHROv5_61151 |  | RadC-like gene | NS | NS |
| Regulation | Gene absent | *lexA* | SOS response transcriptional regulator (repressor) | ND | ND |
| dsDNA repair  Recombination | ARTHROv5_11364 | *recA* | DNA strand exchange and recombination protein, with protease and nuclease activity | NS | NS |
|  | ARTHROv5_20108 | *recJ* | DNA-specific exonuclease | 2,76 | 2,31 |
|  | ARTHROv5_41370 | *recQ* | Putative ATP-dependent DNA helicase, | 1,84 | 2,31 |
|  | ARTHROv5_40176 | *recG* | ATP-dependent DNA helicase | 2,20 | 2,93 |
|  | ARTHROv5_40244 | *holB* | DNA polymerase III, subunit delta prime | 2,09 | 2,45 |
|  | ARTHROv5_40734 | *gyrA* | DNA gyrase (type II topoisomerase), subunit A | 1,97 | 3,60 |
| **ssDNA- repair NER** | ARTHROv5_40732 | *uvrB* | Excinulease of nucleotide excision repair UvrABC system, subunit B, DNA damage recognition component | 3,59 | 4,35 |
|  | ARTHROv5_60258 | *uvrC* | Excinuclease of nucleotide excision repair UvrABC system, subunit C | 3,51 | 2,86 |
| **ssDNA- repair MMR** | ARTHROv5_10136 | *mutS* | DNA mismatch repair protein | 2,15 | 2,87 |
|  | ARTHROv5_40086 | *mutT* | hydrolase/pyrophosphatase, a NUDIX enzyme | 3,74 | 3,45 |
|  | ARTHROv5_61096 | *nudF* | ADP-ribose pyrophosphatase, a NUDIX enzyme | NS | 2,54 |
|  | ARTHROv5_41027 | *uvrD* | UvrD/REP DNA helicase (UvrD-RepA-PcrA like) | 2,17 | 5,42 |
| dsDNA repair Restriction | ARTHROv5_30623 | *hsdR1* | Type I site-specific deoxyribonuclease, HsdR family | 7,25 | 19,90 |
|  | ARTHROv5_30624 | *hsdR2* | Type I site-specific deoxyribonuclease, HsdR family | 13,55 | 11,12 |
|  | ARTHROv5_30625 | *hsdR3* | Type I site-specific deoxyribonuclease, HsdR family | 5,65 | 4,47 |
|  | ARTHROv5_60699 | *hsdM* | Type I restriction-modification system DNA methylase | 3,95 | 1,88 |
|  | ARTHROv5_60700 | *hsdS* | Type I restriction enzyme | 4,00 | 4,27 |
|  | ARTHROv5_50002 | *pvuIIM* | Modification methylase | 2,30 | 4,86 |
|  | ARTHROv5_50004 | *pvuIIR* | Type II restriction enzyme | 2,84 | 9,75 |

Table S7. Transcriptomic (microarray) results for genes known to be involved in genetic rearrangement, in specific genes coding for Transposases. The fold change (FC) values listed are values for which p-value is p<0.05, and are only considered biologically significant if FC > 2 or < 0.5. 'NS' stands for not significant differentially expressed (p>0.05).

| Transposases | Accession number |  | **Protein Function** | Fold change 3200 Gy | Fold change 5000 Gy |
| --- | --- | --- | --- | --- | --- |
|  | ARTHROv5_10410 |  | Transposase, IS630 family (fragment) | 1,37 | 2,53 |
|  | ARTHROv5_10411 |  | Transposase (fragment) | 1,31 | 4,75 |
|  | ARTHROv5_10512 |  | Transposase, IS630 family (fragment) | 1,37 | 6,48 |
|  | ARTHROv5_10564 |  | Transposase, IS630 family (fragment) | 1,31 | 4,56 |
|  | ARTHROv5_10570 |  | Transposase | 1,32 | 3,31 |
|  | ARTHROv5_10577 |  | Transposase | 1,35 | 2,78 |
|  | ARTHROv5_10641 |  | Transposase, IS630 family (fragment) | 1,20 | 3,60 |
|  | ARTHROv5_10881 |  | Transposase (fragment) | 1,66 | 5,66 |
|  | ARTHROv5_10882 |  | Transposase | 1,33 | 2,99 |
|  | ARTHROv5_11071 |  | Transposase, IS605 family, OrfB (fragment) | 0,85 | 2,48 |
|  | ARTHROv5_11514 |  | Transposase | 1,21 | 2,18 |
|  | ARTHROv5_11930 |  | Transposase | 1,49 | 2,32 |
|  | ARTHROv5_11972 |  | Transposase, IS605 family (fragment) | 0,65 | 0,40 |
|  | ARTHROv5_20017 |  | Transposase, IS605 family, OrfB (fragment) | 0,44 | 0,27 |
|  | ARTHROv5_30018 |  | Transposase (fragment) | 2,80 | 2,38 |
|  | ARTHROv5_30092 |  | Transposase, IS630 family (fragment) | 1,29 | 2,95 |
|  | ARTHROv5_30093 |  | Transposase, IS630 family (fragment) | 1,33 | 5,87 |
|  | ARTHROv5_30094 |  | Transposase, IS630 family (fragment) | 1,17 | 3,01 |
|  | ARTHROv5_30127 |  | Transposase, IS630 family (fragment) | 0,44 | 0,30 |
|  | ARTHROv5_30136 |  | Transposase, IS4 family (fragment) | 1,88 | 2,90 |
|  | ARTHROv5_30141 |  | Transposase | 2,64 | 2,02 |
|  | ARTHROv5_40367 |  | Transposase, IS630 family (fragment) | 1,32 | 2,88 |
|  | ARTHROv5_40434 |  | Transposase | 1,26 | 2,72 |
|  | ARTHROv5_40469 |  | Transposase (fragment) | 1,12 | 3,92 |
|  | ARTHROv5_40511 |  | Transposase (fragment) | 1,39 | 2,25 |
|  | ARTHROv5_40799 |  | Transposase (fragment) | 3,42 | 4,00 |
|  | ARTHROv5_40800 |  | Transposase, IS630 family (fragment) | 1,76 | 2,78 |
|  | ARTHROv5_40819 |  | Transposase | 1,22 | 2,08 |
|  | ARTHROv5_41186 |  | Transposase, IS630 family (fragment) | 1,31 | 4,30 |
|  | ARTHROv5_41194 |  | Transposase (fragment) | 1,26 | 2,45 |
|  | ARTHROv5_41214 |  | Transposase (fragment) | 1,45 | 6,76 |
|  | ARTHROv5_41266 |  | Transposase, IS630 family (fragment) | 1,36 | 4,35 |
|  | ARTHROv5_41267 |  | Transposase, IS630 family (fragment) | 1,42 | 3,75 |
|  | ARTHROv5_50016 |  | Transposase (fragment) | 3,43 | 5,25 |
|  | ARTHROv5_50128 |  | Transposase, IS605 family, OrfB (fragment) | 0,91 | 2,28 |
|  | ARTHROv5_60487 |  | Transposase | 1,24 | 3,65 |
|  | ARTHROv5_60945 |  | Transposase | 1,20 | 2,40 |

Table S8. Transcriptomic (microarray) results for genes known to be involved in genetic rearrangement, in specific genes from phage-like (Fax) Elements. In total there are 7 Fax clusters in the genome. The fold change (FC) values listed are values for which p-value is p<0.05, and are only considered biologically significant if FC > 2 or < 0.5. 'NS' stands for not significant differentially expressed (p>0.05).

| FAX Elements | Accession number |  | **Protein Function** | Fold change 3200 Gy | Fold change 5000 Gy |
| --- | --- | --- | --- | --- | --- |
| FAX1 | ARTHROv5_10112 |  | Putative phage tail sheath protein, Gp18-like | NS | 0,25 |
|  | ARTHROv5_10113 |  | Conserved Hypothetical protein | NS | 0,18 |
|  | ARTHROv5_10114 |  | Putative phage tail region protein | NS | 0,26 |
|  | ARTHROv5_10115 |  | Putative phage tail region protein | NS | 0,29 |
|  | ARTHROv5_10116 |  | Conserved Hypothetical protein | 0,46 | 0,33 |
|  | ARTHROv5_10117 |  | Conserved Hypothetical protein | NS | 0,38 |
|  | ARTHROv5_10118 |  | Conserved Hypothetical protein | NS | 0,41 |
|  | ARTHROv5_10119 |  | Conserved Hypothetical protein | NS | NS |
|  | ARTHROv5_10120 |  | Conserved Hypothetical protein | NS | 0,46 |
| FAX2 | ARTHROv5_10166 |  | Conserved Hypothetical protein | NS | NS |
|  | ARTHROv5_10167 | *faxA2* | Unknown phage of the genus *Arthrospira*, protein A | 1,58 | 1,59 |
|  | ARTHROv5_10168 | *faxB2* | Unknown phage of the genus *Arthrospira*, protein B | 2,17 | 1,71 |
|  | ARTHROv5_10169 |  | Conserved Hypothetical protein | 5,94 | NS |
|  | ARTHROv5_10170 |  | Conserved Hypothetical protein | 6,18 | 3,65 |
|  | ARTHROv5_10171 | *faxE2* | Unknown phage of the genus *Arthrospira*, protein E | 4,06 | 1,32 |
|  | ARTHROv5_10172 |  | Conserved Hypothetical protein | NS | NS |
|  | ARTHROv5_10173 |  | Conserved Hypothetical protein | NS | NS |
|  | ARTHROv5_10175 |  | Conserved Hypothetical protein | NS | NS |
|  | ARTHROv5_10176 |  | Conserved Hypothetical protein | NS | NS |
|  | ARTHROv5_10177 | *faxJ2* | Unknown phage of the genus *Arthrospira*, protein J | 0,70 | 1,04 |
| FAX3 | ARTHROv5_10892 | *faxP3* | Unknown phage of the genus *Arthrospira*, protein P | 1,41 | 6,57 |
|  | ARTHROv5_10893 |  | Conserved Hypothetical protein | 4,08 | NS |
|  | ARTHROv5_10894 |  | Conserved Hypothetical protein | NS | NS |
|  | ARTHROv5_10895 |  | Conserved Hypothetical protein | 2,28 | 3,10 |
|  | ARTHROv5_10897 |  | Conserved Hypothetical protein | 8,69 | 5,63 |
|  | ARTHROv5_10898 |  | Conserved Hypothetical protein | 5,21 | 5,36 |
|  | ARTHROv5_10899 |  | Conserved Hypothetical protein | 4,64 | 4,22 |
|  | ARTHROv5_10900 | *faxJ3* | Unknown phage of the genus *Arthrospira*, protein J | 1,16 | 1,74 |
| FAX4 |  |  |  |  |  |
|  | ARTHROv5_20060 | *faxG4* | Protein of fax element | 2,94 | 0,87 |
|  | ARTHROv5_20061 |  | Conserved Hypothetical protein | NS | NS |
|  | ARTHROv5_20062 |  | Conserved Hypothetical protein | NS | NS |
|  | ARTHROv5_20064 | *faxJ4* | Unknown phage of the genus *Arthrospira*, protein J | 0,80 | 1,80 |
|  | ARTHROv5_20065 |  | Conserved Hypothetical protein | 8,11 | NS |
|  | ARTHROv5_20066 |  | Conserved Hypothetical protein | 6,49 | 7,38 |
|  | ARTHROv5_20067 |  | Conserved Hypothetical protein | 10,42 | 10,44 |
|  | ARTHROv5_20070 |  | Conserved Hypothetical protein | 2,35 | 2,28 |
|  | ARTHROv5_20071 |  | Conserved Hypothetical protein | 5,91 | 10,79 |
|  | ARTHROv5_20073 |  | Conserved Hypothetical protein | NS | 3,80 |
| FAX5 | ARTHROv5_30540 |  | Conserved Hypothetical protein | NS | NS |
|  | ARTHROv5_30541 | *faxA5* | Unknown phage of the genus *Arthrospira*, protein A | 1,45 | 1,55 |
|  | ARTHROv5_30544 |  | Conserved Hypothetical protein | NS | NS |
|  | ARTHROv5_30545 | *faxE5* | Unknown phage of the genus *Arthrospira*, protein E | 7,31 | 1,06 |
|  | ARTHROv5_30546 |  | Conserved Hypothetical protein | NS | NS |
|  | ARTHROv5_30549 |  | Conserved Hypothetical protein | NS | NS |
|  | ARTHROv5_30551 | *faxJ5* | Unknown phage of the genus *Arthrospira*, protein J | 0,79 | 1,10 |
|  | ARTHROv5_30552 |  | Conserved Hypothetical protein | 5,20 | 3,19 |
|  | ARTHROv5_30553 | *faxK5f3* | unknown phage of the genus *Arthrospira*, protein K (fragment) | 10,49 | 4,11 |
|  | ARTHROv5_30554 |  | Conserved Hypothetical protein | 8,32 | 5,03 |
|  | ARTHROv5_30555 |  | Conserved Hypothetical protein | NS | 5,36 |
|  | ARTHROv5_30556 |  | Conserved Hypothetical protein | 4,93 | 4,73 |
|  | ARTHROv5_30557 |  | Conserved Hypothetical protein | 5,10 | 7,00 |
|  | ARTHROv5_30558 |  | Conserved Hypothetical protein | 7,60 | 5,83 |
|  | ARTHROv5_30563 |  | Conserved Hypothetical protein | 5,45 | 6,99 |
|  | ARTHROv5_30564 |  | Conserved Hypothetical protein | 5,84 | 8,42 |
|  | ARTHROv5_30565 | *faxP5* | Unknown phage of the genus *Arthrospira*, protein P | 1,31 | 7,64 |
|  | ARTHROv5_30566 |  | Conserved Hypothetical protein | NS | 4,35 |
| FAX6 | ARTHROv5_30729 |  | Conserved Hypothetical protein | NS | 2,10 |
|  | ARTHROv5_30730 | *faxP6* | Unknown phage of the genus *Arthrospira*, protein P | 1,53 | 4,53 |
|  | ARTHROv5_30731 | *faxO6f* | Unknown phage of the genus *Arthrospira*, protein O (fragment) | 4,12 | 7,19 |
|  | ARTHROv5_30732 |  | Conserved Hypothetical protein | 3,15 | 6,95 |
|  | ARTHROv5_30734 | *faxM6f1* | Unknown phage of the genus *Arthrospira*, protein M (fragment) | 3,71 | 3,44 |
|  | ARTHROv5_30735 |  | Conserved Hypothetical protein | 5,06 | NS |
|  | ARTHROv5_30737 |  | Conserved Hypothetical protein | 6,57 | 6,63 |
|  | ARTHROv5_30738 |  | Conserved Hypothetical protein | 6,10 | 6,15 |
|  | ARTHROv5_30740 |  | Conserved Hypothetical protein | 12,77 | 4,50 |
|  | ARTHROv5_30741 |  | Conserved Hypothetical protein | 5,09 | NS |
|  | ARTHROv5_30749 |  | Conserved Hypothetical protein | 7,07 | NS |
|  | ARTHROv5_30751 | *faxB6* | Unknown phage of the genus *Arthrospira*, protein B | 2,67 | 2,90 |
|  | ARTHROv5_30752 | *faxA6* | Unknown phage of the genus *Arthrospira*, protein A | 2,49 | 3,54 |
|  | ARTHROv5_30753 |  | Conserved Hypothetical protein | NS | NS |
| FAX7 | ARTHROv5_40343 | *faxE7* | Unknown phage of the genus *Arthrospira*, protein E | 4,62 | 1,74 |
|  | ARTHROv5_40344 |  | Conserved Hypothetical protein | NS | NS |
|  | ARTHROv5_40345 | *faxG7* | Unknown phage of the genus *Arthrospira*, protein G | 4,76 | NS |
|  | ARTHROv5_40353 | *faxK7f2* | Unknown phage of the genus *Arthrospira*, protein K (fragment) | 5,41 | 6,89 |

Table S9. Transcriptomic (microarray) results for genes known to be involved in genetic rearrangement, in specific genes from CRISPR elements. In total there are 14 CRISPR elements in the genome. The fold change (FC) values listed are values for which p-value is p<0.05, and are only considered biologically significant if FC > 2 or < 0.5. 'NS' stands for not significant differentially expressed (p>0.05).

| CRISPRs | Accession number |  | **Protein Function** | Fold change 3200 Gy | Fold change 5000 Gy |
| --- | --- | --- | --- | --- | --- |
| **CRISPR 1** | ARTHROv5_40676 | *cas2* | CRISPR-associated endoribonuclease Cas2 | 6,76 | 3,34 |
|  | ARTHROv5_40678 | *cas1* | CRISPR-associated endonuclease Cas1 | 3,69 | 2,24 |
|  | ARTHROv5_40688 |  | CRISPR-associated RAMP protein | 2,12 | 2,90 |
|  | ARTHROv5_40690 | *csm3* | CRISPR-associated RAMP protein, Csm3 family | 1,63 | 3,50 |
|  | ARTHROv5_40694 | *csm2* | CRISPR-associated RAMP protein, Crm2 family | 1,83 | 2,71 |
|  | ARTHROv5_40716 | *csm5* | CRISPR-associated RAMP protein, Csm5 family | 1,44 | 2,28 |
|  | ARTHROv5_40717 | *csm4* | CRISPR-associated RAMP protein, Csm4 family protein | 1,56 | 2,65 |
|  | ARTHROv5_40718 | *csm3* | CRISPR-associated RAMP protein, Csm3 family | 1,29 | 3,98 |

Table S10. Transcriptomic (microarray) results for genes coding for conserved hypothetical proteins of unknown function. The fold change (FC) values listed are values for which p-value is p<0.05, and are only considered biologically significant if FC > 2 or < 0.5. 'NS' stands for not significant differentially expressed (p>0.05).

| Conserved hypothetical proteins | Accession number | Gene | Protein Function | Fold change 3200 Gy | Fold change 5000 Gy |
| --- | --- | --- | --- | --- | --- |
|  | ARTHROv5_10002 |  | Conserved protein of unknown function | 1,13 | 2,40 |
|  | ARTHROv5_10012 |  | Conserved hypothetical protein | 0,90 | 0,41 |
|  | ARTHROv5_10037 |  | Conserved protein of unknown function | 1,12 | 2,34 |
|  | ARTHROv5_10048 |  | Conserved hypothetical protein | 2,66 | 3,96 |
|  | ARTHROv5_10050 |  | Conserved hypothetical protein | 1,90 | 4,78 |
|  | ARTHROv5_10055 |  | Conserved hypothetical protein | 1,70 | 2,67 |
|  | ARTHROv5_10066 |  | Conserved hypothetical protein | 1,88 | 2,04 |
|  | ARTHROv5_10068 |  | Conserved hypothetical protein (membrane) | 2,13 | 4,54 |
|  | ARTHROv5_10069 |  | Conserved hypothetical protein (secreted) | 2,19 | 5,99 |
|  | ARTHROv5_10070 |  | Conserved membrane protein of unknown function | 1,53 | 3,28 |
|  | ARTHROv5_10075 |  | Conserved hypothetical protein | 2,13 | 2,62 |
|  | ARTHROv5_10076 |  | Conserved hypothetical protein | 1,94 | 2,14 |
|  | ARTHROv5_10088 |  | Conserved exported protein of unknown function | 0,56 | 0,40 |
|  | ARTHROv5_10089 |  | Conserved hypothetical protein | 0,29 | 0,26 |
|  | ARTHROv5_10090 |  | Conserved hypothetical protein | 0,28 | 0,13 |
|  | ARTHROv5_10102 |  | Conserved hypothetical protein | 2,20 | 3,93 |
|  | ARTHROv5_10107 |  | Conserved hypothetical protein | 3,30 | 13,57 |
|  | ARTHROv5_10113 |  | Conserved hypothetical protein | 0,26 | 0,19 |
|  | ARTHROv5_10116 |  | Conserved hypothetical protein | 0,46 | 0,33 |
|  | ARTHROv5_10117 |  | Conserved hypothetical protein | 0,52 | 0,39 |
|  | ARTHROv5_10122 |  | Conserved hypothetical protein | 0,35 | 0,28 |
|  | ARTHROv5_10134 |  | Conserved protein of unknown function | 1,89 | 2,94 |
|  | ARTHROv5_10145 |  | Conserved protein of unknown function | 3,68 | 2,63 |
|  | ARTHROv5_10179 |  | Conserved protein of unknown function | 5,73 | 5,42 |
|  | ARTHROv5_10180 |  | Conserved protein of unknown function | 6,49 | 6,33 |
|  | ARTHROv5_10185 |  | Conserved protein of unknown function | 3,81 | 7,17 |
|  | ARTHROv5_10186 |  | Conserved protein of unknown function | 1,54 | 5,63 |
|  | ARTHROv5_10187 |  | Conserved protein of unknown function | 1,20 | 8,04 |
|  | ARTHROv5_10199 |  | Conserved hypothetical protein | 0,74 | 0,41 |
|  | ARTHROv5_10200 |  | Conserved hypothetical protein | 0,41 | 0,26 |
|  | ARTHROv5_10201 |  | Conserved hypothetical protein | 0,45 | 0,31 |
|  | ARTHROv5_10204 |  | Conserved protein of unknown function | 0,17 | 0,18 |
|  | ARTHROv5_10208 |  | Conserved hypothetical protein | 0,49 | 0,22 |
|  | ARTHROv5_10209 |  | Conserved hypothetical protein (secreted) | 0,34 | 0,18 |
|  | ARTHROv5_10212 |  | Conserved hypothetical protein | 1,40 | 4,00 |
|  | ARTHROv5_10213 |  | Conserved hypothetical protein (secreted) | 0,58 | 0,45 |
|  | ARTHROv5_10246 |  | Conserved hypothetical protein | 0,23 | 0,16 |
|  | ARTHROv5_10252 |  | Conserved hypothetical protein | 0,70 | 0,37 |
|  | ARTHROv5_10256 |  | Conserved hypothetical protein (membrane) | 3,98 | 5,78 |
|  | ARTHROv5_10284 |  | Conserved protein of unknown function | 1,61 | 2,09 |
|  | ARTHROv5_10286 |  | Conserved protein of unknown function | 3,87 | 2,14 |
|  | ARTHROv5_10288 |  | Conserved hypothetical protein | 1,15 | 2,32 |
|  | ARTHROv5_10290 |  | Conserved hypothetical protein | 1,95 | 2,04 |
|  | ARTHROv5_10292 |  | Conserved hypothetical protein | 2,39 | 3,06 |
|  | ARTHROv5_10295 |  | Conserved hypothetical protein | 0,21 | 0,25 |
|  | ARTHROv5_10299 |  | Conserved hypothetical protein | 0,17 | 0,26 |
|  | ARTHROv5_10300 |  | Conserved protein of unknown function | 0,53 | 0,33 |
|  | ARTHROv5_10308 |  | Conserved hypothetical protein | 0,23 | 0,39 |
|  | ARTHROv5_10338 |  | Conserved exported protein of unknown function | 0,09 | 0,10 |
|  | ARTHROv5_10342 |  | Conserved protein of unknown function | 1,74 | 2,87 |
|  | ARTHROv5_10343 |  | Conserved protein of unknown function | 1,25 | 3,83 |
|  | ARTHROv5_10351 |  | Conserved protein of unknown function | 1,05 | 7,46 |
|  | ARTHROv5_10358 |  | Conserved protein of unknown function | 1,83 | 3,84 |
|  | ARTHROv5_10361 |  | Conserved protein of unknown function | 1,47 | 3,40 |
|  | ARTHROv5_10371 |  | Conserved protein of unknown function | 0,99 | 3,54 |
|  | ARTHROv5_10373 |  | Conserved protein of unknown function | 2,22 | 2,63 |
|  | ARTHROv5_10374 |  | Conserved protein of unknown function | 1,43 | 2,58 |
|  | ARTHROv5_10390 |  | Conserved protein of unknown function | 0,39 | 0,38 |
|  | ARTHROv5_10391 |  | Conserved protein of unknown function | 0,74 | 0,37 |
|  | ARTHROv5_10397 |  | Conserved exported protein of unknown function | 0,66 | 0,33 |
|  | ARTHROv5_10417 |  | Conserved protein of unknown function | 3,82 | 5,81 |
|  | ARTHROv5_10418 |  | Conserved protein of unknown function | 3,69 | 5,46 |
|  | ARTHROv5_10419 |  | Conserved protein of unknown function | 1,95 | 2,77 |
|  | ARTHROv5_10420 |  | Conserved protein of unknown function | 1,73 | 2,18 |
|  | ARTHROv5_10427 |  | Conserved protein of unknown function | 0,81 | 0,41 |
|  | ARTHROv5_10428 |  | Conserved protein of unknown function | 0,57 | 0,41 |
|  | ARTHROv5_10429 |  | Conserved protein of unknown function | 0,49 | 0,34 |
|  | ARTHROv5_10431 |  | Conserved membrane protein of unknown function | 0,50 | 0,17 |
|  | ARTHROv5_10434 |  | Conserved protein of unknown function | 0,29 | 0,24 |
|  | ARTHROv5_10438 |  | Conserved protein of unknown function | 1,21 | 2,40 |
|  | ARTHROv5_10439 |  | Conserved protein of unknown function | 3,44 | 7,79 |
|  | ARTHROv5_10451 |  | Conserved hypothetical protein | 2,60 | 3,08 |
|  | ARTHROv5_10464 |  | Conserved membrane protein of unknown function | 1,42 | 2,74 |
|  | ARTHROv5_10465 |  | Conserved protein of unknown function | 2,68 | 2,76 |
|  | **ARTHROv5_10467** | ***arhF*** | **Conserved hypothetical protein** | **5,10** | **5,75** |
|  | **ARTHROv5_10468** | ***arhE*** | **Conserved hypothetical protein** | **9,58** | **11,99** |
|  | **ARTHROv5_10469** | ***arhD*** | **Conserved hypothetical protein** | **5,44** | **9,46** |
|  | **ARTHROv5_10470** | ***arhC*** | **Conserved hypothetical protein** | **3,14** | **14,62** |
|  | **ARTHROv5_10471** | ***arhB*** | **Conserved hypothetical protein** | **NS** | **22,36** |
|  | ARTHROv5_10474 |  | Conserved protein of unknown function | 4,00 | 4,69 |
|  | ARTHROv5_10480 |  | Conserved hypothetical protein | 0,64 | 0,39 |
|  | ARTHROv5_10482 |  | Conserved hypothetical protein (secreted) | 0,56 | 0,27 |
|  | ARTHROv5_10501 |  | Conserved hypothetical protein | 2,65 | 2,00 |
|  | ARTHROv5_10585 |  | Conserved hypothetical protein | 1,97 | 2,37 |
|  | ARTHROv5_10591 |  | Conserved hypothetical protein (fragment part2) | 1,25 | 3,07 |
|  | ARTHROv5_10602 |  | Conserved hypothetical protein | 0,49 | 0,41 |
|  | ARTHROv5_10660 |  | Conserved protein of unknown function | 2,59 | 4,42 |
|  | ARTHROv5_10669 |  | Conserved hypothetical protein (fragment part 2) | 0,50 | 0,40 |
|  | ARTHROv5_10677 |  | Conserved hypothetical protein | 0,81 | 0,45 |
|  | ARTHROv5_10684 |  | Conserved hypothetical protein | 0,42 | 0,38 |
|  | ARTHROv5_10692 |  | Conserved protein of unknown function | 0,37 | 0,30 |
|  | ARTHROv5_10699 |  | Conserved hypothetical protein | 0,47 | 0,37 |
|  | ARTHROv5_10721 |  | Conserved hypothetical protein (membrane) | 3,24 | 2,45 |
|  | ARTHROv5_10743 |  | Conserved hypothetical protein | 4,64 | 10,15 |
|  | ARTHROv5_10764 |  | Conserved hypothetical protein | 3,20 | 2,57 |
|  | ARTHROv5_10765 |  | Conserved hypothetical protein | 1,37 | 3,17 |
|  | ARTHROv5_10778 |  | Conserved protein of unknown function | 0,80 | 0,31 |
|  | ARTHROv5_10782 |  | Conserved exported protein of unknown function | 0,26 | 0,22 |
|  | ARTHROv5_10794 |  | Conserved hypothetical protein | 2,15 | 2,49 |
|  | ARTHROv5_10802 |  | Conserved hypothetical protein | 0,91 | 2,01 |
|  | ARTHROv5_10834 |  | Conserved hypothetical protein | 0,83 | 0,46 |
|  | ARTHROv5_10841 |  | Conserved hypothetical protein | 0,43 | 0,23 |
|  | ARTHROv5_10842 |  | Conserved hypothetical protein | 0,37 | 0,21 |
|  | ARTHROv5_10843 |  | Conserved hypothetical protein, CofD related | 0,65 | 0,28 |
|  | ARTHROv5_10861 |  | Conserved hypothetical protein (membrane) | 1,52 | 0,23 |
|  | ARTHROv5_10926 |  | Conserved protein of unknown function | 1,03 | 5,22 |
|  | ARTHROv5_10927 |  | Conserved protein of unknown function | 1,94 | 3,88 |
|  | ARTHROv5_10928 |  | Conserved hypothetical protein | 2,09 | 3,06 |
|  | ARTHROv5_10929 |  | Conserved hypothetical protein | 2,35 | 3,99 |
|  | ARTHROv5_10930 |  | Conserved hypothetical protein | 2,26 | 3,49 |
|  | ARTHROv5_10931 |  | Conserved hypothetical protein | 1,90 | 2,97 |
|  | ARTHROv5_10933 |  | Conserved protein of unknown function | 7,49 | 6,42 |
|  | ARTHROv5_10937 |  | Conserved hypothetical protein (fragment) | 1,89 | 3,97 |
|  | ARTHROv5_10938 |  | Conserved hypothetical protein | 1,53 | 2,31 |
|  | ARTHROv5_10940 |  | Conserved hypothetical protein | 1,38 | 2,11 |
|  | ARTHROv5_10950 |  | Conserved hypothetical protein (membrane) | 3,04 | 3,69 |
|  | ARTHROv5_10980 |  | Conserved protein of unknown function | 1,36 | 2,39 |
|  | ARTHROv5_10983 |  | Conserved hypothetical protein | 0,55 | 0,28 |
|  | ARTHROv5_10995 |  | Conserved hypothetical protein (secreted) | 0,81 | 0,45 |
|  | ARTHROv5_11000 |  | Conserved hypothetical protein, NnrU-like | 0,77 | 0,20 |
|  | ARTHROv5_11002 |  | Conserved hypothetical protein | 0,63 | 0,32 |
|  | ARTHROv5_11013 |  | Conserved hypothetical protein | 0,74 | 0,38 |
|  | ARTHROv5_11024 |  | Conserved protein of unknown function | 1,37 | 8,59 |
|  | ARTHROv5_11026 |  | Conserved hypothetical protein (secreted) | 2,40 | 3,45 |
|  | ARTHROv5_11066 |  | Conserved protein of unknown function | 6,04 | 3,79 |
|  | ARTHROv5_11076 |  | Conserved hypothetical protein | 0,81 | 0,34 |
|  | ARTHROv5_11088 |  | Conserved protein of unknown function | 3,19 | 3,77 |
|  | ARTHROv5_11089 |  | Conserved protein of unknown function | 3,95 | 7,44 |
|  | ARTHROv5_11093 |  | Conserved protein of unknown function | 0,65 | 0,34 |
|  | ARTHROv5_11104 |  | Conserved hypothetical protein | 2,17 | 4,68 |
|  | ARTHROv5_11121 |  | Conserved hypothetical protein (membrane) | 0,46 | 0,21 |
|  | ARTHROv5_11123 |  | Conserved hypothetical protein | 0,43 | 0,23 |
|  | ARTHROv5_11190 |  | Conserved hypothetical protein | 2,19 | 3,00 |
|  | ARTHROv5_11195 |  | Conserved hypothetical protein (fragment) | 1,05 | 0,47 |
|  | ARTHROv5_11205 |  | Conserved protein of unknown function | 1,93 | 2,20 |
|  | ARTHROv5_11207 |  | Conserved protein of unknown function | 5,48 | 2,78 |
|  | ARTHROv5_11208 |  | Conserved hypothetical protein (fragment) | 2,96 | 2,13 |
|  | ARTHROv5_11209 |  | Conserved hypothetical protein | 2,29 | 2,84 |
|  | ARTHROv5_11215 |  | Conserved protein of unknown function | 2,30 | 2,39 |
|  | ARTHROv5_11230 |  | Conserved hypothetical protein | 0,90 | 2,59 |
|  | ARTHROv5_11233 |  | Conserved hypothetical protein | 0,76 | 2,49 |
|  | ARTHROv5_11234 |  | Conserved hypothetical protein | 1,30 | 2,43 |
|  | ARTHROv5_11250 |  | Conserved protein of unknown function | 0,45 | 0,37 |
|  | ARTHROv5_11251 |  | Conserved protein of unknown function | 0,49 | 0,37 |
|  | ARTHROv5_11283 |  | Conserved hypothetical protein | 0,64 | 0,26 |
|  | ARTHROv5_11315 |  | Conserved hypothetical protein (secreted) | 0,16 | 0,15 |
|  | ARTHROv5_11349 |  | Conserved protein of unknown function | 2,01 | 2,22 |
|  | ARTHROv5_11374 |  | Conserved protein of unknown function | 1,00 | 2,77 |
|  | ARTHROv5_11390 |  | Conserved hypothetical protein | 1,03 | 0,24 |
|  | ARTHROv5_11404 |  | Conserved protein of unknown function | 1,45 | 2,42 |
|  | ARTHROv5_11492 |  | Conserved hypothetical protein | 1,06 | 6,49 |
|  | ARTHROv5_11510 |  | Conserved hypothetical protein | 0,70 | 0,37 |
|  | ARTHROv5_11511 |  | Conserved hypothetical protein (membrane) | 0,61 | 0,26 |
|  | ARTHROv5_11562 |  | Conserved hypothetical protein | 1,46 | 2,46 |
|  | ARTHROv5_11564 |  | Conserved hypothetical protein | 0,41 | 0,44 |
|  | ARTHROv5_11574 |  | Conserved protein of unknown function | 1,43 | 2,27 |
|  | ARTHROv5_11581 |  | Conserved protein of unknown function | 1,30 | 2,00 |
|  | ARTHROv5_11588 |  | Conserved protein of unknown function | 1,45 | 2,61 |
|  | ARTHROv5_11599 |  | Conserved protein of unknown function | 0,90 | 2,25 |
|  | ARTHROv5_11600 |  | Conserved protein of unknown function | 0,81 | 2,36 |
|  | ARTHROv5_11610 |  | Conserved hypothetical protein | 0,76 | 0,38 |
|  | ARTHROv5_11614 |  | Conserved hypothetical protein | 0,42 | 0,21 |
|  | ARTHROv5_11621 |  | Conserved hypothetical protein | 0,66 | 0,28 |
|  | ARTHROv5_11635 |  | Conserved hypothetical protein (secreted) | 0,59 | 0,39 |
|  | ARTHROv5_11653 |  | Conserved hypothetical protein | 0,56 | 0,34 |
|  | ARTHROv5_11662 |  | Conserved hypothetical protein | 0,73 | 0,45 |
|  | ARTHROv5_11664 |  | Conserved hypothetical protein | 1,78 | 2,15 |
|  | ARTHROv5_11679 |  | Conserved hypothetical protein (membrane) | 1,02 | 0,45 |
|  | ARTHROv5_11681 |  | Conserved hypothetical protein | 0,44 | 0,27 |
|  | ARTHROv5_11695 |  | Conserved hypothetical protein | 2,84 | 2,53 |
|  | ARTHROv5_11706 |  | Conserved hypothetical protein | 0,67 | 0,43 |
|  | ARTHROv5_11732 |  | Conserved protein of unknown function | 0,50 | 0,39 |
|  | ARTHROv5_11734 |  | Conserved membrane protein of unknown function | 3,43 | 2,73 |
|  | ARTHROv5_11742 |  | Conserved hypothetical protein | 3,80 | 3,86 |
|  | ARTHROv5_11754 |  | Conserved hypothetical protein | 1,34 | 2,45 |
|  | ARTHROv5_11755 |  | Conserved hypothetical protein | 2,29 | 3,31 |
|  | ARTHROv5_11807 |  | Conserved hypothetical protein | 0,07 | 0,19 |
|  | ARTHROv5_11858 |  | Conserved hypothetical protein | 1,69 | 3,15 |
|  | ARTHROv5_11879 |  | Conserved hypothetical protein | 0,09 | 0,08 |
|  | ARTHROv5_11882 |  | Conserved protein of unknown function | 0,30 | 0,10 |
|  | ARTHROv5_11892 |  | Conserved hypothetical protein | 0,59 | 0,32 |
|  | ARTHROv5_11904 |  | Conserved hypothetical protein | 0,46 | 0,39 |
|  | ARTHROv5_11913 |  | Conserved hypothetical protein | 0,29 | 0,39 |
|  | ARTHROv5_11939 |  | Conserved hypothetical protein (membrane) | 1,04 | 0,33 |
|  | ARTHROv5_11940 |  | Conserved hypothetical protein (membrane) | 1,04 | 0,39 |
|  | ARTHROv5_11941 |  | Conserved hypothetical protein (membrane) | 0,62 | 0,47 |
|  | ARTHROv5_11945 |  | Conserved hypothetical protein | 0,71 | 4,30 |
|  | ARTHROv5_11957 |  | Conserved hypothetical protein | 0,51 | 0,32 |
|  | ARTHROv5_12006 |  | Conserved protein of unknown function | 1,64 | 2,66 |
|  | ARTHROv5_12009 |  | Conserved protein of unknown function | 1,26 | 5,72 |
|  | ARTHROv5_12018 |  | Conserved hypothetical protein | 0,38 | 0,37 |
|  | ARTHROv5_12026 |  | Conserved hypothetical protein | 0,51 | 0,43 |
|  | ARTHROv5_12034 |  | Conserved protein of unknown function | 0,25 | 0,23 |
|  | ARTHROv5_12051 |  | Conserved membrane protein of unknown function | 1,33 | 0,33 |
|  | ARTHROv5_12064 |  | Conserved hypothetical protein (membrane) | 0,32 | 0,38 |
|  | ARTHROv5_12065 |  | Conserved exported protein of unknown function | 0,20 | 0,16 |
|  | ARTHROv5_12066 |  | Conserved hypothetical protein (fragment) | 0,12 | 0,13 |
|  | ARTHROv5_12100 |  | Conserved protein of unknown function | 1,15 | 2,80 |
|  | ARTHROv5_12102 |  | Conserved protein of unknown function | 0,94 | 0,38 |
|  | ARTHROv5_12115 |  | Conserved protein of unknown function | 1,35 | 2,28 |
|  | ARTHROv5_12137 |  | Conserved hypothetical protein | 0,41 | 0,34 |
|  | ARTHROv5_20001 |  | Conserved hypothetical protein (membrane) | 0,85 | 0,28 |
|  | ARTHROv5_20029 |  | Conserved hypothetical protein | 0,42 | 0,15 |
|  | ARTHROv5_20030 |  | Conserved protein of unknown function | 0,58 | 0,25 |
|  | ARTHROv5_20031 |  | Conserved hypothetical protein | 0,81 | 0,46 |
|  | ARTHROv5_20035 |  | Conserved hypothetical protein | 0,37 | 0,27 |
|  | ARTHROv5_20042 |  | Conserved protein of unknown function | 2,73 | 2,65 |
|  | ARTHROv5_20066 |  | Conserved protein of unknown function | 6,50 | 7,39 |
|  | ARTHROv5_20073 |  | Conserved protein of unknown function | 1,31 | 3,81 |
|  | ARTHROv5_20085 |  | Conserved hypothetical protein | 2,74 | 3,41 |
|  | ARTHROv5_20100 |  | Conserved hypothetical protein | 5,15 | 6,84 |
|  | ARTHROv5_20102 |  | Conserved protein of unknown function | 0,06 | 0,09 |
|  | ARTHROv5_20106 |  | Conserved protein of unknown function | 2,31 | 2,13 |
|  | ARTHROv5_20107 |  | Conserved protein of unknown function | 2,25 | 2,11 |
|  | ARTHROv5_20109 |  | Conserved protein of unknown function | 1,22 | 2,15 |
|  | ARTHROv5_20110 |  | Conserved protein of unknown function | 1,50 | 5,93 |
|  | ARTHROv5_20119 |  | Conserved hypothetical protein | 0,89 | 0,47 |
|  | ARTHROv5_20132 |  | Conserved membrane protein of unknown function | 0,86 | 0,49 |
|  | ARTHROv5_20133 |  | Conserved protein of unknown function | 0,72 | 0,42 |
|  | ARTHROv5_20134 |  | Conserved protein of unknown function | 0,70 | 0,34 |
|  | ARTHROv5_20135 |  | Conserved protein of unknown function | 0,94 | 0,43 |
|  | ARTHROv5_20137 |  | Conserved protein of unknown function | 0,38 | 0,30 |
|  | ARTHROv5_20143 |  | Conserved protein of unknown function | 0,70 | 0,41 |
|  | ARTHROv5_20161 |  | Conserved protein of unknown function | 0,92 | 2,20 |
|  | ARTHROv5_20171 |  | Conserved hypothetical protein | 0,54 | 0,32 |
|  | ARTHROv5_20188 |  | Conserved exported protein of unknown function | 2,80 | 2,44 |
|  | ARTHROv5_20196 |  | Conserved hypothetical protein | 0,15 | 0,14 |
|  | ARTHROv5_20216 |  | Conserved hypothetical protein | 0,28 | 0,40 |
|  | ARTHROv5_20222 |  | Conserved protein of unknown function | 1,50 | 2,25 |
|  | ARTHROv5_20235 |  | Conserved protein of unknown function | 0,20 | 0,40 |
|  | ARTHROv5_20236 |  | Conserved protein of unknown function | 0,35 | 0,27 |
|  | ARTHROv5_20238 |  | Conserved hypothetical protein | 0,42 | 0,30 |
|  | ARTHROv5_20244 |  | Conserved hypothetical protein | 0,52 | 0,34 |
|  | ARTHROv5_20251 |  | Conserved protein of unknown function | 1,93 | 2,62 |
|  | ARTHROv5_30011 |  | Conserved hypothetical protein (secreted) | 1,26 | 2,18 |
|  | ARTHROv5_30015 |  | Conserved hypothetical protein | 0,99 | 5,48 |
|  | ARTHROv5_30019 |  | Conserved hypothetical protein | 5,58 | 6,74 |
|  | ARTHROv5_30027 |  | Conserved hypothetical protein | 0,69 | 0,20 |
|  | ARTHROv5_30045 |  | Conserved hypothetical protein | 0,54 | 0,29 |
|  | ARTHROv5_30055 |  | Conserved hypothetical protein | 0,38 | 0,33 |
|  | ARTHROv5_30063 |  | Conserved hypothetical protein | 1,08 | 2,37 |
|  | ARTHROv5_30072 |  | Conserved hypothetical protein | 2,66 | 2,93 |
|  | ARTHROv5_30075 |  | Conserved protein of unknown function | 0,27 | 0,20 |
|  | ARTHROv5_30116 |  | Conserved hypothetical protein | 0,34 | 0,44 |
|  | ARTHROv5_30180 |  | Conserved hypothetical protein | 0,70 | 0,39 |
|  | ARTHROv5_30181 |  | Conserved hypothetical protein | 0,54 | 0,24 |
|  | ARTHROv5_30201 |  | Conserved protein of unknown function | 1,33 | 0,17 |
|  | ARTHROv5_30204 |  | Conserved hypothetical protein | 0,19 | 0,13 |
|  | ARTHROv5_30208 |  | Conserved membrane protein of unknown function | 0,58 | 0,38 |
|  | ARTHROv5_30230 |  | Conserved protein of unknown function | 1,55 | 2,99 |
|  | ARTHROv5_30234 |  | Conserved hypothetical protein | 0,52 | 0,30 |
|  | ARTHROv5_30246 |  | Conserved hypothetical protein | 0,62 | 0,45 |
|  | ARTHROv5_30251 |  | Conserved hypothetical protein | 0,47 | 0,24 |
|  | ARTHROv5_30332 |  | Conserved hypothetical protein | 0,79 | 0,46 |
|  | ARTHROv5_30388 |  | Conserved hypothetical protein | 1,00 | 0,28 |
|  | ARTHROv5_30405 |  | Conserved protein of unknown function | 0,32 | 0,32 |
|  | ARTHROv5_30406 |  | Conserved hypothetical protein | 0,55 | 0,48 |
|  | ARTHROv5_30449 |  | Conserved hypothetical protein (fragment) | 3,08 | 6,21 |
|  | ARTHROv5_30489 |  | Conserved hypothetical protein | 0,63 | 0,49 |
|  | ARTHROv5_30512 |  | Conserved hypothetical protein | 2,80 | 2,19 |
|  | ARTHROv5_30538 |  | Conserved protein of unknown function | 0,54 | 0,32 |
|  | ARTHROv5_30552 |  | Conserved protein of unknown function | 5,21 | 3,19 |
|  | ARTHROv5_30554 |  | Conserved protein of unknown function | 8,33 | 5,03 |
|  | ARTHROv5_30555 |  | Conserved protein of unknown function | 3,62 | 5,37 |
|  | ARTHROv5_30556 |  | Conserved protein of unknown function | 4,94 | 4,74 |
|  | ARTHROv5_30558 |  | Conserved protein of unknown function | 7,60 | 5,83 |
|  | ARTHROv5_30564 |  | Conserved protein of unknown function | 5,84 | 8,43 |
|  | ARTHROv5_30566 |  | Conserved protein of unknown function | 1,50 | 4,36 |
|  | ARTHROv5_30571 |  | Conserved hypothetical protein (exported ) | 2,32 | 2,47 |
|  | ARTHROv5_30576 |  | Conserved protein of unknown function | 0,79 | 0,48 |
|  | ARTHROv5_30582 |  | Conserved hypothetical protein | 0,61 | 0,36 |
|  | ARTHROv5_30585 |  | Conserved protein of unknown function | 2,96 | 2,75 |
|  | ARTHROv5_30588 |  | Conserved hypothetical protein | 1,94 | 2,70 |
|  | ARTHROv5_30589 |  | Conserved hypothetical protein | 1,85 | 2,66 |
|  | ARTHROv5_30595 |  | Conserved protein of unknown function | 3,86 | 3,85 |
|  | ARTHROv5_30612 |  | Conserved protein of unknown function | 0,76 | 0,36 |
|  | ARTHROv5_30621 |  | Conserved hypothetical protein | 3,39 | 28,21 |
|  | ARTHROv5_30622 |  | Conserved hypothetical protein (fragment) | 4,63 | 38,06 |
|  | ARTHROv5_30631 |  | Conserved hypothetical protein | 1,84 | 2,04 |
|  | ARTHROv5_30632 |  | Conserved hypothetical protein | 0,67 | 0,29 |
|  | ARTHROv5_30635 |  | Conserved hypothetical protein | 0,59 | 0,42 |
|  | ARTHROv5_30649 |  | Conserved hypothetical protein (bifunctional) | 4,25 | 17,29 |
|  | ARTHROv5_30655 |  | Conserved protein of unknown function | 0,51 | 0,29 |
|  | ARTHROv5_30674 |  | Conserved protein of unknown function | 0,35 | 0,27 |
|  | ARTHROv5_30702 |  | Conserved protein of unknown function | 2,90 | 2,63 |
|  | ARTHROv5_30713 |  | Conserved hypothetical protein | 0,59 | 0,41 |
|  | ARTHROv5_30737 |  | Conserved protein of unknown function | 6,58 | 6,64 |
|  | ARTHROv5_30738 |  | Conserved exported protein of unknown function | 6,11 | 6,15 |
|  | ARTHROv5_30762 |  | Conserved protein of unknown function | 0,70 | 0,41 |
|  | ARTHROv5_30763 |  | Conserved hypothetical protein | 0,54 | 0,36 |
|  | ARTHROv5_30768 |  | Conserved hypothetical protein | 0,73 | 0,37 |
|  | ARTHROv5_30781 |  | Conserved hypothetical protein | 1,67 | 4,67 |
|  | ARTHROv5_30782 |  | Conserved hypothetical protein (fragment) | 1,64 | 4,85 |
|  | ARTHROv5_30784 |  | Conserved hypothetical protein (fragment) | 1,76 | 5,55 |
|  | ARTHROv5_30785 |  | Conserved hypothetical protein | 3,25 | 4,90 |
|  | ARTHROv5_30786 |  | Conserved hypothetical protein (fragment) | 1,78 | 6,13 |
|  | ARTHROv5_30824 |  | Conserved hypothetical protein | 1,69 | 5,32 |
|  | ARTHROv5_30836 |  | Conserved protein of unknown function | 4,32 | 25,56 |
|  | ARTHROv5_30856 |  | Conserved hypothetical protein | 3,82 | 7,45 |
|  | ARTHROv5_30858 |  | Conserved protein of unknown function | 2,99 | 14,78 |
|  | ARTHROv5_30859 |  | Conserved hypothetical protein | 2,23 | 16,62 |
|  | ARTHROv5_30860 |  | Conserved protein of unknown function | 1,96 | 9,23 |
|  | ARTHROv5_30861 |  | Conserved protein of unknown function | 2,42 | 3,87 |
|  | ARTHROv5_40002 |  | Conserved hypothetical protein | 0,60 | 0,46 |
|  | ARTHROv5_40029 |  | Conserved hypothetical protein | 0,66 | 0,45 |
|  | ARTHROv5_40038 |  | Conserved hypothetical protein | 2,78 | 4,55 |
|  | ARTHROv5_40039 |  | Conserved hypothetical protein | 2,25 | 4,10 |
|  | ARTHROv5_40043 |  | Conserved hypothetical protein | 1,82 | 5,42 |
|  | ARTHROv5_40073 |  | Conserved exported protein of unknown function | 0,87 | 0,42 |
|  | ARTHROv5_40111 |  | Conserved protein of unknown function | 1,15 | 5,06 |
|  | ARTHROv5_40118 |  | Conserved hypothetical protein (membrane) | 1,13 | 2,52 |
|  | ARTHROv5_40136 |  | Conserved protein of unknown function | 0,63 | 0,41 |
|  | ARTHROv5_40154 |  | Conserved hypothetical protein | 0,66 | 0,33 |
|  | ARTHROv5_40156 |  | Conserved hypothetical protein | 0,72 | 0,41 |
|  | ARTHROv5_40159 |  | Conserved hypothetical protein | 0,76 | 0,26 |
|  | ARTHROv5_40179 |  | Conserved hypothetical protein | 0,77 | 0,48 |
|  | ARTHROv5_40189 |  | Conserved hypothetical protein (secreted) | 0,66 | 0,31 |
|  | ARTHROv5_40216 |  | Conserved hypothetical protein | 1,65 | 2,09 |
|  | ARTHROv5_40226 |  | Conserved hypothetical protein | 0,94 | 0,44 |
|  | ARTHROv5_40242 |  | Conserved hypothetical protein | 0,33 | 0,26 |
|  | ARTHROv5_40245 |  | Conserved hypothetical protein | 1,79 | 10,4 |
|  | ARTHROv5_40246 |  | Conserved hypothetical protein | 1,08 | 10,9 |
|  | ARTHROv5_40247 |  | Conserved hypothetical protein | 0,81 | 9,49 |
|  | ARTHROv5_40248 |  | Conserved hypothetical protein | 0,96 | 10,0 |
|  | ARTHROv5_40249 |  | putative [Myosin heavy-chain] kinase | NS | 3,44 |
|  | ARTHROv5_40250 |  | Conserved hypothetical protein | 4,69 | 9,01 |
|  | ARTHROv5_40251 |  | Conserved hypothetical protein | 2,21 | 4,12 |
|  | ARTHROv5_40256 |  | Conserved protein of unknown function | 1,89 | 2,26 |
|  | ARTHROv5_40285 |  | Conserved exported protein of unknown function | 0,59 | 0,37 |
|  | ARTHROv5_40289 |  | Conserved protein of unknown function | 0,48 | 0,24 |
|  | ARTHROv5_40306 |  | Conserved hypothetical protein | 1,22 | 5,67 |
|  | ARTHROv5_40308 |  | Conserved hypothetical protein (fragment) | 1,35 | 3,11 |
|  | ARTHROv5_40333 |  | Conserved membrane protein of unknown function | 0,31 | 0,18 |
|  | ARTHROv5_40334 |  | Conserved protein of unknown function | 0,16 | 0,15 |
|  | ARTHROv5_40339 |  | Conserved protein of unknown function | 2,17 | 3,91 |
|  | ARTHROv5_40364 |  | Conserved hypothetical protein | 0,69 | 0,31 |
|  | ARTHROv5_40384 |  | Conserved hypothetical protein (secreted) | 2,20 | 2,63 |
|  | ARTHROv5_40424 |  | Conserved hypothetical protein | 3,41 | 4,74 |
|  | ARTHROv5_40427 |  | Conserved hypothetical protein | 1,74 | 2,98 |
|  | ARTHROv5_40430 |  | Conserved hypothetical protein (membrane) | 0,42 | 0,42 |
|  | ARTHROv5_40431 |  | Conserved hypothetical protein (membrane) | 0,37 | 0,30 |
|  | ARTHROv5_40447 |  | Conserved hypothetical protein (membrane) | 2,39 | 3,96 |
|  | ARTHROv5_40448 |  | Conserved hypothetical protein | 2,81 | 3,10 |
|  | ARTHROv5_40455 |  | Conserved hypothetical protein | 0,10 | 0,17 |
|  | ARTHROv5_40465 |  | Conserved hypothetical protein | 1,86 | 4,03 |
|  | ARTHROv5_40477 |  | Conserved hypothetical protein (fragment) | 0,21 | 0,23 |
|  | ARTHROv5_40480 |  | Conserved hypothetical protein | 1,62 | 2,12 |
|  | ARTHROv5_40526 |  | Conserved hypothetical protein | 1,81 | 2,71 |
|  | ARTHROv5_40534 |  | Conserved hypothetical protein | 0,24 | 0,31 |
|  | ARTHROv5_40543 |  | Conserved protein of unknown function | 0,97 | 0,36 |
|  | ARTHROv5_40544 |  | Conserved hypothetical protein | 0,88 | 6,35 |
|  | ARTHROv5_40546 |  | Conserved hypothetical protein | 1,42 | 2,88 |
|  | ARTHROv5_40547 |  | Conserved hypothetical protein (membrane) | 3,13 | 2,44 |
|  | ARTHROv5_40550 |  | Conserved protein of unknown function | 1,57 | 3,71 |
|  | ARTHROv5_40553 |  | Conserved protein of unknown function | 0,81 | 0,41 |
|  | ARTHROv5_40574 |  | Conserved hypothetical protein, PatB-like | 0,43 | 0,06 |
|  | ARTHROv5_40575 |  | Conserved hypothetical protein, PatC-like | 0,42 | 0,05 |
|  | ARTHROv5_40576 |  | Conserved hypothetical protein | 0,53 | 0,10 |
|  | ARTHROv5_40577 |  | Conserved protein of unknown function | 0,74 | 0,10 |
|  | ARTHROv5_40578 |  | Conserved protein of unknown function | 0,63 | 0,12 |
|  | ARTHROv5_40579 |  | Conserved protein of unknown function | 0,84 | 0,08 |
|  | ARTHROv5_40584 |  | Conserved protein of unknown function | 0,67 | 0,37 |
|  | ARTHROv5_40585 |  | Conserved protein of unknown function | 0,71 | 0,43 |
|  | ARTHROv5_40586 |  | Conserved protein of unknown function | 0,95 | 0,43 |
|  | ARTHROv5_40587 |  | Conserved hypothetical protein | 0,53 | 0,37 |
|  | ARTHROv5_40588 |  | Conserved protein of unknown function | 0,68 | 0,38 |
|  | ARTHROv5_40589 |  | Conserved protein of unknown function | 0,73 | 0,39 |
|  | ARTHROv5_40595 |  | Conserved hypothetical protein | 2,03 | 2,80 |
|  | ARTHROv5_40596 |  | Conserved hypothetical protein | 2,50 | 2,97 |
|  | ARTHROv5_40598 |  | Conserved hypothetical protein | 1,55 | 4,13 |
|  | ARTHROv5_40604 |  | Conserved hypothetical protein | 2,61 | 4,62 |
|  | ARTHROv5_40605 |  | Conserved hypothetical protein | 1,71 | 2,49 |
|  | ARTHROv5_40614 |  | Conserved hypothetical protein | 1,22 | 3,66 |
|  | ARTHROv5_40615 |  | Conserved hypothetical protein | 1,41 | 11,19 |
|  | ARTHROv5_40623 |  | Conserved hypothetical protein | 1,52 | 2,02 |
|  | ARTHROv5_40637 |  | Conserved hypothetical protein | 2,05 | 2,98 |
|  | ARTHROv5_40638 |  | Conserved hypothetical protein | 1,30 | 4,60 |
|  | ARTHROv5_40650 |  | Conserved hypothetical protein | 0,75 | 3,31 |
|  | ARTHROv5_40651 |  | Conserved hypothetical protein | 2,16 | 2,78 |
|  | ARTHROv5_40654 |  | Conserved hypothetical protein | 2,64 | 2,29 |
|  | ARTHROv5_40655 |  | Conserved hypothetical protein | 4,52 | 3,19 |
|  | ARTHROv5_40666 |  | Conserved hypothetical protein | 1,51 | 3,82 |
|  | ARTHROv5_40667 |  | Conserved protein of unknown function | 2,23 | 3,10 |
|  | ARTHROv5_40671 |  | Conserved protein of unknown function | 0,85 | 0,21 |
|  | ARTHROv5_40684 |  | Conserved protein of unknown function | 2,68 | 4,64 |
|  | ARTHROv5_40685 |  | Conserved hypothetical protein | 1,70 | 7,09 |
|  | ARTHROv5_40686 |  | Conserved protein of unknown function | 2,65 | 3,73 |
|  | ARTHROv5_40687 |  | Conserved hypothetical protein | 2,75 | 2,68 |
|  | ARTHROv5_40689 |  | Conserved protein of unknown function | 1,43 | 3,33 |
|  | ARTHROv5_40700 |  | Conserved hypothetical protein | 1,43 | 5,02 |
|  | ARTHROv5_40702 |  | Conserved hypothetical protein | 1,81 | 4,72 |
|  | ARTHROv5_40705 |  | Conserved hypothetical protein | 2,05 | 3,96 |
|  | ARTHROv5_40719 |  | Conserved hypothetical protein | 1,09 | 7,21 |
|  | ARTHROv5_40731 |  | Conserved protein of unknown function | 1,94 | 2,70 |
|  | ARTHROv5_40735 |  | Conserved hypothetical protein | 1,40 | 2,15 |
|  | ARTHROv5_40736 |  | Conserved hypothetical protein | 0,85 | 2,14 |
|  | ARTHROv5_40745 |  | Conserved hypothetical protein | 1,98 | 5,24 |
|  | ARTHROv5_40787 |  | Conserved hypothetical protein | 0,22 | 0,19 |
|  | ARTHROv5_40788 |  | Conserved hypothetical protein | 0,90 | 0,45 |
|  | ARTHROv5_40811 |  | Conserved protein of unknown function | 3,48 | 6,09 |
|  | ARTHROv5_40934 |  | Conserved protein of unknown function | 0,63 | 0,31 |
|  | ARTHROv5_40936 |  | Conserved protein of unknown function | 0,63 | 0,19 |
|  | ARTHROv5_40952 |  | Conserved exported protein of unknown function | 0,26 | 0,15 |
|  | ARTHROv5_40957 |  | Conserved protein of unknown function | 1,81 | 3,92 |
|  | ARTHROv5_40958 |  | Conserved protein of unknown function | 3,06 | 3,34 |
|  | ARTHROv5_40977 |  | Conserved hypothetical protein | 1,42 | 2,00 |
|  | ARTHROv5_40982 |  | Conserved hypothetical protein | 3,45 | 4,61 |
|  | ARTHROv5_41037 |  | Conserved hypothetical protein (secreted) | 0,65 | 0,32 |
|  | ARTHROv5_41044 |  | Conserved hypothetical protein | 1,34 | 5,22 |
|  | ARTHROv5_41045 |  | Conserved hypothetical protein | 2,10 | 2,76 |
|  | ARTHROv5_41051 |  | Conserved hypothetical protein | 1,57 | 2,64 |
|  | ARTHROv5_41056 |  | Conserved hypothetical protein (exported) | 2,33 | 3,99 |
|  | ARTHROv5_41064 |  | Conserved protein of unknown function | 0,49 | 0,30 |
|  | ARTHROv5_41081 |  | Conserved hypothetical protein | 3,81 | 6,03 |
|  | ARTHROv5_41094 |  | Conserved hypothetical protein | 1,27 | 3,48 |
|  | ARTHROv5_41097 |  | Conserved protein of unknown function | 3,02 | 6,81 |
|  | ARTHROv5_41098 |  | Conserved protein of unknown function | 2,91 | 6,10 |
|  | ARTHROv5_41115 |  | Conserved protein of unknown function | 3,53 | 3,10 |
|  | ARTHROv5_41129 |  | Conserved protein of unknown function | 5,80 | 4,17 |
|  | ARTHROv5_41132 |  | Conserved protein of unknown function | 1,36 | 3,10 |
|  | ARTHROv5_41138 |  | Conserved protein of unknown function | 0,45 | 0,25 |
|  | ARTHROv5_41144 |  | Conserved hypothetical protein | 0,33 | 0,37 |
|  | ARTHROv5_41154 |  | Conserved exported protein of unknown function | 0,14 | 0,28 |
|  | ARTHROv5_41176 |  | Conserved protein of unknown function | 0,11 | 0,23 |
|  | ARTHROv5_41220 |  | Conserved protein of unknown function | 0,35 | 0,44 |
|  | ARTHROv5_41237 |  | Conserved hypothetical protein (fragment part 2) | 3,45 | 6,29 |
|  | ARTHROv5_41238 |  | Conserved hypothetical protein (fragment part 3) | 3,16 | 4,23 |
|  | ARTHROv5_41281 |  | Conserved hypothetical protein (fragment) | 0,79 | 0,32 |
|  | ARTHROv5_41282 |  | Conserved hypothetical protein (fragment) | 0,61 | 0,15 |
|  | ARTHROv5_41330 |  | Conserved hypothetical protein | 0,31 | 0,44 |
|  | ARTHROv5_41337 |  | Conserved hypothetical protein | 0,54 | 0,36 |
|  | ARTHROv5_41366 |  | Conserved hypothetical protein | 0,97 | 0,23 |
|  | ARTHROv5_41371 |  | Conserved hypothetical protein | 1,01 | 4,67 |
|  | ARTHROv5_41372 |  | Conserved hypothetical protein | 2,05 | 4,20 |
|  | ARTHROv5_41395 |  | Conserved protein of unknown function | 0,28 | 0,25 |
|  | ARTHROv5_41410 |  | Conserved hypothetical protein | 0,52 | 0,43 |
|  | ARTHROv5_41429 |  | Conserved hypothetical protein (membrane) | 2,23 | 3,20 |
|  | ARTHROv5_41432 |  | Conserved hypothetical protein | 1,25 | 2,78 |
|  | ARTHROv5_41439 |  | Conserved protein of unknown function | 1,40 | 2,71 |
|  | ARTHROv5_41441 |  | Conserved exported protein of unknown function | 3,06 | 5,43 |
|  | ARTHROv5_50005 |  | Conserved hypothetical protein | 2,39 | 11,19 |
|  | ARTHROv5_50019 |  | Conserved protein of unknown function | 1,36 | 2,14 |
|  | ARTHROv5_50047 |  | Conserved hypothetical protein | 3,54 | 4,68 |
|  | ARTHROv5_50048 |  | Conserved hypothetical protein (fragment) | 3,46 | 4,00 |
|  | ARTHROv5_50049 |  | Conserved hypothetical protein (fragment) | 4,12 | 9,63 |
|  | ARTHROv5_50050 |  | Conserved hypothetical protein (fragment) | 3,03 | 5,52 |
|  | ARTHROv5_50051 |  | Conserved hypothetical protein (fragment) | 3,36 | 4,08 |
|  | ARTHROv5_50059 |  | Conserved hypothetical protein | 1,24 | 2,76 |
|  | ARTHROv5_50076 |  | Conserved hypothetical protein (secreted) | 1,10 | 0,39 |
|  | ARTHROv5_50112 |  | Conserved hypothetical protein (secreted) | 0,92 | 0,39 |
|  | ARTHROv5_50120 |  | Conserved protein of unknown function | 0,57 | 0,45 |
|  | ARTHROv5_50122 |  | Conserved hypothetical protein | 0,55 | 0,43 |
|  | ARTHROv5_50137 |  | Conserved protein of unknown function | 1,62 | 2,05 |
|  | ARTHROv5_50145 |  | Conserved hypothetical protein | 0,52 | 0,39 |
|  | ARTHROv5_50147 |  | Conserved hypothetical protein | 0,88 | 0,43 |
|  | ARTHROv5_50175 |  | Conserved hypothetical protein | 3,63 | 9,50 |
|  | ARTHROv5_50182 |  | Conserved hypothetical protein | 1,56 | 2,20 |
|  | ARTHROv5_50183 |  | Conserved hypothetical protein (fragment) | 1,34 | 2,26 |
|  | ARTHROv5_50185 |  | Conserved hypothetical protein (fragment) | 2,13 | 3,69 |
|  | ARTHROv5_50186 |  | Conserved hypothetical protein | 2,14 | 5,09 |
|  | ARTHROv5_50187 |  | Conserved hypothetical protein | 1,54 | 4,44 |
|  | ARTHROv5_50199 |  | Conserved hypothetical protein | 1,05 | 2,87 |
|  | ARTHROv5_50247 |  | Conserved protein of unknown function | 0,38 | 0,38 |
|  | ARTHROv5_50273 |  | Conserved hypothetical protein | 0,47 | 0,36 |
|  | ARTHROv5_50292 |  | Conserved hypothetical protein | 2,10 | 2,78 |
|  | ARTHROv5_50296 |  | Conserved hypothetical protein (fragment) | 3,74 | 3,13 |
|  | ARTHROv5_50300 |  | Conserved protein of unknown function | 6,58 | 5,93 |
|  | ARTHROv5_50301 |  | Conserved protein of unknown function | 1,93 | 9,66 |
|  | ARTHROv5_50313 |  | Conserved protein of unknown function | 2,73 | 3,45 |
|  | ARTHROv5_50314 |  | Conserved protein of unknown function | 3,00 | 3,76 |
|  | ARTHROv5_50315 |  | Conserved protein of unknown function | 2,95 | 4,78 |
|  | ARTHROv5_50326 |  | Conserved protein of unknown function | 0,81 | 0,43 |
|  | ARTHROv5_50330 |  | Conserved membrane protein of unknown function | 0,51 | 0,44 |
|  | ARTHROv5_50341 |  | Conserved protein of unknown function | 0,80 | 0,44 |
|  | ARTHROv5_50345 |  | Conserved protein of unknown function | 1,43 | 3,23 |
|  | ARTHROv5_50346 |  | Conserved exported protein of unknown function | 1,08 | 3,17 |
|  | ARTHROv5_50348 |  | Conserved membrane protein of unknown function | 0,68 | 0,35 |
|  | ARTHROv5_60009 |  | Conserved protein of unknown function | 0,76 | 0,38 |
|  | ARTHROv5_60032 |  | Conserved exported protein of unknown function | 0,47 | 0,23 |
|  | ARTHROv5_60039 |  | Conserved protein of unknown function | 0,63 | 0,41 |
|  | ARTHROv5_60052 |  | Conserved protein of unknown function | 1,06 | 2,04 |
|  | ARTHROv5_60060 |  | Conserved protein of unknown function | 1,57 | 0,43 |
|  | ARTHROv5_60066 |  | Conserved hypothetical protein | 0,42 | 0,38 |
|  | ARTHROv5_60068 |  | Conserved hypothetical protein | 0,90 | 2,40 |
|  | ARTHROv5_60070 |  | Conserved hypothetical protein | 0,44 | 0,25 |
|  | ARTHROv5_60103 |  | Conserved hypothetical protein | 0,35 | 0,21 |
|  | ARTHROv5_60116 |  | Conserved protein of unknown function | 0,76 | 0,22 |
|  | ARTHROv5_60128 |  | Conserved hypothetical protein | 3,39 | 10,28 |
|  | ARTHROv5_60177 |  | Conserved hypothetical protein (membrane) | 0,04 | 0,04 |
|  | ARTHROv5_60195 |  | Conserved protein of unknown function | 1,44 | 2,55 |
|  | ARTHROv5_60200 |  | Conserved hypothetical protein | 1,71 | 2,49 |
|  | ARTHROv5_60201 |  | Conserved hypothetical protein | 1,26 | 2,37 |
|  | ARTHROv5_60207 |  | Conserved hypothetical protein | 0,60 | 0,36 |
|  | ARTHROv5_60260 |  | Conserved protein of unknown function | 2,23 | 2,47 |
|  | ARTHROv5_60261 |  | Conserved protein of unknown function | 2,71 | 4,93 |
|  | ARTHROv5_60263 |  | Conserved protein of unknown function | 3,50 | 2,07 |
|  | ARTHROv5_60270 |  | Conserved membrane protein of unknown function | 3,92 | 5,54 |
|  | ARTHROv5_60273 |  | Conserved exported protein of unknown function | 1,30 | 2,27 |
|  | ARTHROv5_60290 |  | Conserved hypothetical protein | 0,76 | 0,36 |
|  | ARTHROv5_60305 |  | Conserved protein of unknown function | 0,40 | 0,29 |
|  | ARTHROv5_60319 |  | Conserved hypothetical protein | 2,45 | 2,81 |
|  | ARTHROv5_60328 |  | Conserved protein of unknown function | 1,12 | 0,46 |
|  | ARTHROv5_60329 |  | Conserved protein of unknown function | 4,20 | 3,23 |
|  | ARTHROv5_60330 |  | Conserved protein of unknown function | 4,47 | 3,90 |
|  | ARTHROv5_60331 |  | Conserved protein of unknown function | 2,14 | 2,04 |
|  | ARTHROv5_60346 |  | Conserved protein of unknown function | 4,09 | 2,19 |
|  | ARTHROv5_60347 |  | Conserved protein of unknown function | 8,50 | 4,07 |
|  | ARTHROv5_60348 |  | Conserved protein of unknown function | 5,36 | 4,67 |
|  | ARTHROv5_60367 |  | Conserved protein of unknown function | 3,39 | 4,45 |
|  | ARTHROv5_60377 |  | Conserved protein of unknown function | 0,83 | 2,83 |
|  | ARTHROv5_60379 |  | Conserved protein of unknown function | 1,91 | 2,80 |
|  | ARTHROv5_60381 |  | Conserved protein of unknown function | 0,96 | 4,49 |
|  | ARTHROv5_60390 |  | Conserved protein of unknown function | 0,62 | 0,29 |
|  | ARTHROv5_60406 |  | Conserved protein of unknown function | 1,62 | 2,30 |
|  | ARTHROv5_60408 |  | Conserved protein of unknown function | 1,47 | 2,04 |
|  | ARTHROv5_60411 |  | Conserved protein of unknown function | 0,84 | 0,37 |
|  | ARTHROv5_60418 |  | Conserved protein of unknown function | 0,52 | 0,47 |
|  | ARTHROv5_60460 |  | Conserved protein of unknown function | 1,21 | 4,85 |
|  | ARTHROv5_60504 |  | Conserved protein of unknown function | 3,09 | 4,78 |
|  | ARTHROv5_60507 |  | Conserved protein of unknown function | 3,61 | 3,22 |
|  | ARTHROv5_60508 |  | Conserved protein of unknown function | 6,94 | 8,08 |
|  | ARTHROv5_60509 |  | Conserved protein of unknown function | 6,84 | 7,57 |
|  | ARTHROv5_60510 |  | Conserved protein of unknown function | 4,59 | 8,05 |
|  | ARTHROv5_60511 |  | Conserved protein of unknown function | 2,73 | 4,83 |
|  | ARTHROv5_60512 |  | Conserved protein of unknown function | 1,34 | 3,34 |
|  | ARTHROv5_60514 |  | Conserved protein of unknown function | 0,63 | 18,28 |
|  | ARTHROv5_60515 |  | Conserved protein of unknown function | 1,49 | 10,60 |
|  | ARTHROv5_60516 |  | Conserved protein of unknown function | 2,47 | 6,74 |
|  | ARTHROv5_60517 |  | Conserved protein of unknown function | 1,35 | 6,37 |
|  | ARTHROv5_60518 |  | Conserved protein of unknown function | 2,36 | 5,36 |
|  | ARTHROv5_60519 |  | Conserved protein of unknown function | 2,07 | 3,15 |
|  | ARTHROv5_60520 |  | Conserved protein of unknown function | 1,45 | 4,16 |
|  | ARTHROv5_60521 |  | Conserved protein of unknown function | 1,58 | 7,45 |
|  | ARTHROv5_60546 |  | Conserved hypothetical protein | 0,64 | 0,28 |
|  | ARTHROv5_60614 |  | Conserved protein of unknown function | 0,62 | 0,46 |
|  | ARTHROv5_60616 |  | Conserved protein of unknown function | 2,06 | 2,74 |
|  | ARTHROv5_60621 |  | Conserved protein of unknown function | 1,68 | 2,68 |
|  | ARTHROv5_60623 |  | Conserved protein of unknown function | 2,89 | 3,03 |
|  | ARTHROv5_60628 |  | Conserved protein of unknown function | 1,55 | 10,05 |
|  | ARTHROv5_60631 |  | Conserved protein of unknown function | 0,34 | 0,40 |
|  | ARTHROv5_60647 |  | Conserved protein of unknown function | 1,60 | 2,09 |
|  | ARTHROv5_60648 |  | Conserved protein of unknown function | 1,49 | 2,27 |
|  | ARTHROv5_60652 |  | Conserved protein of unknown function | 0,55 | 0,44 |
|  | ARTHROv5_60688 |  | Conserved protein of unknown function | 2,99 | 2,35 |
|  | ARTHROv5_60689 |  | Conserved exported protein of unknown function | 3,67 | 3,09 |
|  | ARTHROv5_60697 |  | Conserved exported protein of unknown function | 1,53 | 3,05 |
|  | ARTHROv5_60708 |  | Conserved protein of unknown function | 0,22 | 0,10 |
|  | ARTHROv5_60717 |  | Conserved protein of unknown function | 0,41 | 0,14 |
|  | ARTHROv5_60739 |  | Conserved hypothetical protein | 0,66 | 0,39 |
|  | ARTHROv5_60741 |  | Conserved hypothetical protein | 0,49 | 0,47 |
|  | ARTHROv5_60755 |  | Conserved hypothetical protein | 5,86 | 10,81 |
|  | ARTHROv5_60766 |  | Conserved protein of unknown function | 3,57 | 5,22 |
|  | ARTHROv5_60767 |  | Conserved hypothetical protein (fragment) | 2,39 | 6,82 |
|  | ARTHROv5_60768 |  | Conserved hypothetical protein | 2,85 | 8,99 |
|  | ARTHROv5_60769 |  | Conserved hypothetical protein | 2,26 | 4,71 |
|  | ARTHROv5_60770 |  | Conserved hypothetical protein | 1,25 | 2,64 |
|  | ARTHROv5_60771 |  | Conserved hypothetical protein (fragment) | 4,67 | 3,70 |
|  | ARTHROv5_60772 |  | Conserved hypothetical protein | 4,99 | 4,23 |
|  | ARTHROv5_60773 |  | Conserved hypothetical protein | 6,07 | 7,21 |
|  | ARTHROv5_60774 |  | Conserved hypothetical protein | 1,99 | 3,30 |
|  | ARTHROv5_60775 |  | Conserved protein of unknown function | 5,80 | 5,44 |
|  | ARTHROv5_60776 |  | Conserved hypothetical protein | 0,95 | 3,31 |
|  | ARTHROv5_60778 |  | Conserved hypothetical protein (fragment) | 1,85 | 4,81 |
|  | ARTHROv5_60779 |  | Conserved hypothetical protein | 1,89 | 9,32 |
|  | ARTHROv5_60780 |  | Conserved hypothetical protein | 1,75 | 15,73 |
|  | ARTHROv5_60781 |  | Conserved hypothetical protein | 4,21 | 19,69 |
|  | ARTHROv5_60786 |  | Conserved hypothetical protein | 2,68 | 3,69 |
|  | ARTHROv5_60788 |  | Conserved hypothetical protein | 1,00 | 4,43 |
|  | ARTHROv5_60818 |  | Conserved hypothetical protein | 2,13 | 14,71 |
|  | ARTHROv5_60819 |  | Conserved hypothetical protein | 3,63 | 8,69 |
|  | ARTHROv5_60823 |  | Conserved hypothetical protein | 0,43 | 0,35 |
|  | ARTHROv5_60824 |  | Conserved hypothetical protein | 0,29 | 0,16 |
|  | ARTHROv5_60831 |  | Conserved protein of unknown function | 0,46 | 0,30 |
|  | ARTHROv5_60838 |  | Conserved hypothetical protein | 1,53 | 2,03 |
|  | ARTHROv5_60840 |  | Conserved hypothetical protein | 1,35 | 6,50 |
|  | ARTHROv5_60841 |  | Conserved hypothetical protein (fragment) | 4,38 | 2,83 |
|  | ARTHROv5_60845 |  | Conserved hypothetical protein | 8,97 | 14,03 |
|  | ARTHROv5_60846 |  | Conserved hypothetical protein | 8,05 | 10,21 |
|  | ARTHROv5_60847 |  | Conserved hypothetical protein(fragment) | 1,45 | 4,30 |
|  | ARTHROv5_60848 |  | Conserved hypothetical protein (fragment) | 11,31 | 18,79 |
|  | ARTHROv5_60849 |  | Conserved hypothetical protein (fragment) | 14,92 | 23,44 |
|  | ARTHROv5_60850 |  | Conserved protein of unknown function | 2,72 | 4,93 |
|  | ARTHROv5_60855 |  | Conserved protein of unknown function | 3,49 | 5,24 |
|  | ARTHROv5_60859 |  | Conserved hypothetical protein | 3,93 | 3,20 |
|  | ARTHROv5_60879 |  | Conserved protein of unknown function | 1,27 | 3,02 |
|  | ARTHROv5_60890 |  | Conserved hypothetical protein | 4,03 | 2,99 |
|  | ARTHROv5_60894 |  | Conserved protein of unknown function | 1,40 | 2,19 |
|  | ARTHROv5_60901 |  | Conserved hypothetical protein | 0,48 | 0,41 |
|  | ARTHROv5_60915 |  | Conserved hypothetical protein | 1,40 | 2,12 |
|  | ARTHROv5_60925 |  | Conserved hypothetical protein | 1,38 | 0,45 |
|  | ARTHROv5_60928 |  | Conserved hypothetical protein | 0,83 | 0,49 |
|  | ARTHROv5_60944 |  | Conserved membrane protein of unknown function | 1,01 | 0,19 |
|  | ARTHROv5_60946 |  | Conserved protein of unknown function | 3,77 | 4,76 |
|  | ARTHROv5_60990 |  | Conserved hypothetical protein | 1,52 | 2,41 |
|  | ARTHROv5_60997 |  | Conserved protein of unknown function | 0,11 | 0,11 |
|  | ARTHROv5_60998 |  | Conserved hypothetical protein (secreted) | 0,31 | 0,18 |
|  | ARTHROv5_61015 |  | Conserved hypothetical protein (membrane) | 0,64 | 0,43 |
|  | ARTHROv5_61021 |  | Conserved hypothetical protein | 2,52 | 3,39 |
|  | ARTHROv5_61024 |  | Conserved protein of unknown function | 1,95 | 2,84 |
|  | ARTHROv5_61038 |  | Conserved hypothetical protein | 2,05 | 4,02 |
|  | ARTHROv5_61042 |  | Conserved hypothetical protein | 2,77 | 5,54 |
|  | ARTHROv5_61043 |  | Conserved hypothetical protein | 2,25 | 3,81 |
|  | ARTHROv5_61046 |  | Conserved hypothetical protein | 3,16 | 5,12 |
|  | ARTHROv5_61049 |  | Conserved protein of unknown function | 2,36 | 6,26 |
|  | ARTHROv5_61050 |  | Conserved protein of unknown function | 5,07 | 5,55 |
|  | ARTHROv5_61051 |  | Conserved hypothetical protein (fragment) | 2,32 | 4,10 |
|  | ARTHROv5_61067 |  | Conserved hypothetical protein | 1,23 | 3,40 |
|  | ARTHROv5_61071 |  | Conserved hypothetical protein | 0,50 | 0,49 |
|  | ARTHROv5_61073 |  | Conserved hypothetical protein (membrane) | 0,33 | 0,17 |
|  | ARTHROv5_61111 |  | Conserved protein of unknown function | 0,47 | 0,43 |
|  | ARTHROv5_61138 |  | Conserved hypothetical protein | 2,99 | 9,05 |
|  | ARTHROv5_61167 |  | Conserved hypothetical protein (membrane) | 0,70 | 0,49 |
|  | ARTHROv5_61170 |  | Conserved hypothetical protein | 0,34 | 0,23 |
|  | ARTHROv5_61196 |  | Conserved hypothetical protein | 0,84 | 0,19 |
|  | ARTHROv5_61202 |  | Conserved protein of unknown function | 2,55 | 3,14 |
|  | ARTHROv5_61205 |  | Conserved exported protein of unknown function | 0,30 | 0,29 |
|  | ARTHROv5_61207 |  | Conserved hypothetical protein (membrane) | 0,70 | 0,19 |
|  | ARTHROv5_61208 |  | Conserved hypothetical protein (fragment) | 0,77 | 0,29 |
|  | ARTHROv5_61217 |  | Conserved protein of unknown function | 0,53 | 0,26 |

Table S11. Proteomics results for 3200 Gy, listed according to gene number. The fold change (FC) values listed are values for which p-value is p<0.05, and are only considered biologically significant if FC > 1.25 or < 0.8. 'NS' stands for not significant differentially expressed (p>0.05).

| Proteomics  3200 Gy | Accession number | **Gene** | **Protein Function** | Fold change 3200 Gy | Peptides coverage |
| --- | --- | --- | --- | --- | --- |
|  | ARTHROv5_10213 |  | Conserved hypothetical protein (secrete) | 0,73 | 4 |
|  | **ARTHROv5_10468** | ***arhE*** | **Conserved hypothetical protein** | **7,12** | **4** |
|  | **ARTHROv5_10470** | ***arhC*** | **Conserved hypothetical protein** | **1,40** | **10** |
|  | **ARTHROv5_10471** | ***arhB*** | **Conserved hypothetical protein** | **1,75** | **9** |
|  | ARTHROv5_10600 |  | Hypothetical protein | 0,67 | 3 |
|  | ARTHROv5_10939 |  | Putative bacterioferritin | 1,44 | 2 |
|  | ARTHROv5_10941 |  | Hypothetical protein (secreted) | 0,44 | 2 |
|  | ARTHROv5_10970 |  | Conserved hypothetical protein | 2,04 | 1 |
|  | ARTHROv5_10983 |  | Conserved hypothetical protein | 1,30 | 3 |
|  | ARTHROv5_11338 |  | Peptidase C14 caspase catalytic subunit p20 | 0,60 | 4 |
|  | ARTHROv5_11504 |  | Conserved hypothetical protein | 0,78 | 2 |
|  | ARTHROv5_11981 |  | Putative membrane-associated zinc metallopeptidase, M50 family | 1,77 | 4 |
|  | ARTHROv5_30042 |  | Putative extracellular nuclease (fragment) | 0,56 | 12 |
|  | ARTHROv5_30080 | *psaD* | Photosystem I reaction center subunit II | 1,45 | 10 |
|  | ARTHROv5_30816 |  | Conserved hypothetical protein | 0,70 | 6 |
|  | ARTHROv5_30849 |  | Type II protein secretion system protein | 0,44 | 2 |
|  | ARTHROv5_40132 | *purM* | Phosphoribosylaminoimidazole synthetase | 1,64 | 1 |
|  | ARTHROv5_40888 |  | Putative serine protease inhibitor family protein | 0,56 | 1 |
|  | ARTHROv5_41069 | *ilvB* | Acetolactate synthase large subunit | 0,73 | 4 |
|  | ARTHROv5_41076 |  | Putative hydrolase | 1,84 | 12 |
|  | ARTHROv5_41179 |  | Conserved hypothetical protein (membrane). | 1,41 | 3 |
|  | ARTHROv5_41229 |  | Conserved hypothetical protein | 0,55 | 1 |
|  | ARTHROv5_41296 | *hcp* | Hydroxylamine reductase, hybrid-cluster [4Fe-2S-2O] protein in anaerobic terminal reductases | 0,69 | 1 |
|  | ARTHROv5_60030 |  | Conserved exported protein of unknown function | 0,67 | 3 |
|  | ARTHROv5_60064 |  | Na-Ca exchanger/integrin-beta4 | 0,73 | 12 |
|  | ARTHROv5_60608 |  | Conserved exported protein of unknown function | 0,45 | 6 |
|  | ARTHROv5_60622 | *nifU* | Nitrogen-fixing protein | 0,73 | 2 |
|  | ARTHROv5_60737 |  | Conserved hypothetical protein | 0,55 | 1 |
|  | ARTHROv5_60836 |  | D-alanyl-D-alanine carboxypeptidase/D-alanyl-D-alanine-endopeptidas | 0,69 | 7 |
|  | ARTHROv5_61026 | *thiC* | Thiamine biosynthesis protein | 1,33 | 4 |
|  | ARTHROv5_61150 |  | putative haemolysin-type calcium-binding toxin, RTX-like | 2,13 | 8 |

Table S12. Proteomics results for 5000 Gy, listed according to gene number. The fold change (FC) values listed are values for which p-value is p<0.05, and are only considered biologically significant if FC > 1.25 or < 0.8. 'NS' stands for not significant differentially expressed (p>0.05).

| Proteomics 5000 gy | Accession number | **Gene** | **Protein Function** | Fold change 5000 Gy | Peptides coverage |
| --- | --- | --- | --- | --- | --- |
|  | ARTHROv5_10064 | *leuC* | 3-isopropylmalate dehydratase large subunit | 1,37 | 5 |
|  | ARTHROv5_10148 | *rpsA1* | 30S ribosomal protein S1 | 0,77 | 10 |
|  | ARTHROv5_10208 |  | Conserved hypothetical protein | 0,47 | 2 |
|  | ARTHROv5_10269 | *pntAA* | Pyridine nucleotide transhydrogenase, alpha subunit, soluble domain | 0,78 | 4 |
|  | **ARTHROv5_10468** | ***arhE*** | **Conserved hypothetical protein** | **4,30** | **3** |
|  | ARTHROv5_10752 | *dapB* | Dihydrodipicolinate reductase | 1,81 | 3 |
|  | ARTHROv5_10984 | *psaA* | Photosystem I P700 chlorophyll a apoprotein A1 (PsaA) | 1,71 | 9 |
|  | ARTHROv5_11019 |  | Hypothetical protein | 0,61 | 8 |
|  | ARTHROv5_11549 | *ssb* | Single-stranded DNA-binding protein | 0,74 | 5 |
|  | ARTHROv5_11629 |  | Putative glycosyl transferase, family 2 | 0,36 | 1 |
|  | ARTHROv5_11670 | *gmd* | GDP-D-mannose dehydratase, NAD(P)-binding | 0,62 | 5 |
|  | ARTHROv5_11794 |  | Putative phytoene dehydrogenase / carotene isomerase | 1,52 | 3 |
|  | ARTHROv5_11902 |  | Conserved hypothetical protein | 1,88 | 1 |
|  | ARTHROv5_11966 |  | Macrophage migration inhibitory factor family protein | 1,43 | 1 |
|  | ARTHROv5_11987 |  | Peptidase C11 clostripain | 0,14 | 1 |
|  | ARTHROv5_11991 |  | Putative Peptidyl-prolyl cis-trans isomerase, cyclophilin family | 0,54 | 3 |
|  | ARTHROv5_11993 | *psbD1* | Photosystem II reaction center D2 protein Q(A) | 1,64 | 8 |
|  | ARTHROv5_30042 |  | Putative extracellular nuclease (fragment) | 0,41 | 10 |
|  | ARTHROv5_30080 | *psaD* | Photosystem I reaction center subunit II | 2,07 | 4 |
|  | ARTHROv5_30191 | *pheT* | Phenylalanine tRNA synthetase, beta subunit | 0,65 | 2 |
|  | ARTHROv5_30386 |  | Conserved hypothetical protein (secreted) | 0,73 | 6 |
|  | ARTHROv5_30727 | *hisF* | Imidazole glycerol phosphate synthase subunit | 0,66 | 1 |
|  | ARTHROv5_30849 |  | Type II protein secretion system protein | 0,27 | 1 |
|  | ARTHROv5_40785 |  | Conserved hypothetical protein | 0,73 | 5 |
|  | ARTHROv5_41102 | *purM* | Putative short-chain dehydrogenase/reductase | 1,61 | 2 |
|  | ARTHROv5_41150 |  | Putative Methylenetetrahydrofolate reductase [NAD(P)H] | 0,67 | 1 |
|  | ARTHROv5_50101 |  | Putative structural maintenance of chromosomes (SMC) protein | 0,60 | 2 |
|  | ARTHROv5_50156 |  | Putative Haemolysin-type calcium-binding toxin, RTX-like | 0,43 | 3 |
|  | ARTHROv5_50171 | *pgl* | 6-phosphogluconolactonase (6PGL) | 0,78 | 6 |
|  | ARTHROv5_60256 | *hup3* | Histone-like bacterial DNA-binding protein, HU-like | 0,75 | 3 |
|  | ARTHROv5_60301 |  | Conserved protein of unknown function | 0,43 | 1 |
|  | ARTHROv5_60318 |  | Putative acireductone dioxygenase | 0,68 | 1 |
|  | ARTHROv5_60608 |  | Conserved exported protein of unknown function | 0,61 | 4 |
|  | ARTHROv5_60992 | *ndk* | Nucleoside diphosphate kinase | 1,31 | 6 |
|  | ARTHROv5_60999 |  | Conserved hypothetical protein | 0,63 | 1 |
|  | ARTHROv5_61122 |  | Conserved hypothetical protein | 0,58 | 1 |
